# RNA Editome in Rhesus Macaque Shaped by Purifying Selection

**DOI:** 10.1371/journal.pgen.1004274

**Published:** 2014-04-10

**Authors:** Jia-Yu Chen, Zhiyu Peng, Rongli Zhang, Xin-Zhuang Yang, Bertrand Chin-Ming Tan, Huaying Fang, Chu-Jun Liu, Mingming Shi, Zhi-Qiang Ye, Yong E. Zhang, Minghua Deng, Xiuqin Zhang, Chuan-Yun Li

**Affiliations:** 1 Beijing Key Laboratory of Cardiometabolic Molecular Medicine, Institute of Molecular Medicine, Peking University, Beijing, China; 2 BGI-Guangzhou, Guangzhou, China; 3 BGI-Shenzhen, Shenzhen, China; 4 Department of Biomedical Sciences and Graduate Institute of Biomedical Sciences, College of Medicine, Chang Gung University, Tao-Yuan, Taiwan; 5 School of Mathematical Sciences and Center for Quantitative Biology, Peking University, Beijing, China; 6 Lab of Computational Chemistry and Drug Design, Key Laboratory of Chemical Genomics, Peking University Shenzhen Graduate School, Shenzhen, China; 7 Key Laboratory of Zoological Systematics and Evolution, Institute of Zoology, Chinese Academy of Sciences, Beijing, China; Stanford University, United States of America

## Abstract

Understanding of the RNA editing process has been broadened considerably by the next generation sequencing technology; however, several issues regarding this regulatory step remain unresolved – the strategies to accurately delineate the editome, the mechanism by which its profile is maintained, and its evolutionary and functional relevance. Here we report an accurate and quantitative profile of the RNA editome for rhesus macaque, a close relative of human. By combining genome and transcriptome sequencing of multiple tissues from the same animal, we identified 31,250 editing sites, of which 99.8% are A-to-G transitions. We verified 96.6% of editing sites in coding regions and 97.5% of randomly selected sites in non-coding regions, as well as the corresponding levels of editing by multiple independent means, demonstrating the feasibility of our experimental paradigm. Several lines of evidence supported the notion that the adenosine deamination is associated with the macaque editome – A-to-G editing sites were flanked by sequences with the attributes of *ADAR* substrates, and both the sequence context and the expression profile of *ADARs* are relevant factors in determining the quantitative variance of RNA editing across different sites and tissue types. In support of the functional relevance of some of these editing sites, substitution valley of decreased divergence was detected around the editing site, suggesting the evolutionary constraint in maintaining some of these editing substrates with their double-stranded structure. These findings thus complement the “continuous probing” model that postulates tinkering-based origination of a small proportion of functional editing sites. In conclusion, the macaque editome reported here highlights RNA editing as a widespread functional regulation in primate evolution, and provides an informative framework for further understanding RNA editing in human.

## Author Summary

RNA editing is a co-transcriptional process that introduces differences between RNA and its corresponding DNA sequence. Currently, the next generation sequencing have allowed study of the editome in a comprehensive and efficient manner. However, fundamental issues involving accurate mapping of the editome as well as its regulation and functional outcome remain unresolved. To further unveil the underlying mechanisms from the evolutionary perspective, we report here the editome profile in rhesus macaque, one of our closest evolutionary relatives. We identified a list of 31,250 RNA-editing sites and deciphered an accurate and informative editome across multiple tissues and animals. We found that the adenosine deamination is associated with the macaque editome, in that both the sequence context and the expression profile of *ADARs* are relevant factors in determining the quantitative variance of RNA editing across different sites and tissue types. Importantly, some of these RNA-editing events represent functional regulation, rather than neutral signals, as suggested by substitution valley of decreased divergence detected around the editing sites, an indication of selective constraint in maintaining some of these editing substrates with their double-stranded structure. The macaque editome thus provides an informative evolutionary context for an in-depth understanding of RNA editing regulation.

## Introduction

Since its discovery in 1986 [1], an increasing number of genes have been found to be subject to RNA editing, a co-transcriptional process that alters hereditary information by introducing differences between RNA and its corresponding DNA sequence [2]. The investigation of such regulation accelerated dramatically after the development of next generation sequencing (NGS) technology, which facilitates the genome-wide determination of DNA and RNA sequences at relatively low cost [3]–[5]. Several early NGS-based editome studies in human [4]–[7] have challenged the traditional view of human genetics, since RNA editing might be a source of variations inaccessible to previous genetic studies.

Although the identification of RNA-editing sites by discerning sequence discrepancies between RNA and DNA derived from the same specimen seems to be a straightforward approach, it is error-prone when the RNA/DNA sequences are compiled by short reads generated from NGS technology [5]. As is being extensively discussed [5], [8]–[12], widespread RNA-editing sites detected in a recent study might be largely a result of technical errors. It thus remains technically challenging to accurately identify human editing sites using NGS data [5], [8]–[12]. In addition, given the difficulties of obtaining specimens of different tissues from the same human individual as well as accurately quantifying the levels of editing using merely NGS data, the mechanisms by which the editome is maintained and regulated remain unclear. Recent studies with contrasting findings are thus in line with the notion that RNA editome may be governed by complex regulation, despite the fact that a large proportion of non-canonical editing types were identified due to technical errors [5]. First, large cross-tissue variations of the RNA editome were detected [4], while the tissue-biased RNA editome could not be directly explained by the expression and activity of known enzymes catalyzing adenosine deamination [13], [14]. Second, genome-wide editome analysis in human also suggested large intra-population variations [3], [7], whereas one study on candidate genes demonstrated otherwise [15]. Third, as only sporadic functional RNA-editing sites have been reported, it remains controversial whether the editing events detected by NGS represent functional regulation rather than neutral signals [16]. The “continuous probing” model postulated that most of the editing sites are neutral with low editing levels, acting as a selection pool for a few functional editing sites [17], further challenged the functional significance of those widespread RNA-editing sites detected by NGS.

Overall, NGS technology has helped open the Pandora's box of the editome and so has raised more questions than it answers. Key issues, including experimental and computational strategies to accurately identify the editome, the mechanism by which its profile is maintained, and its functional significance, are presently not well addressed [18]. Cross-species comparisons with our close evolutionary relatives would provide a framework to clarify these issues. Therefore, we set out to study the editome in rhesus macaque, with the aim of complementing several recent reports on human editome [4]–[7]. The macaque editome we report here provides an important evolutionary context to understand RNA-editing regulation in human, emphasizing RNA editing as a form of widespread functional regulation shaped by purifying selection.

## Results

### Genome-wide identification of RNA-editing sites in rhesus macaque

**Figure 1:**
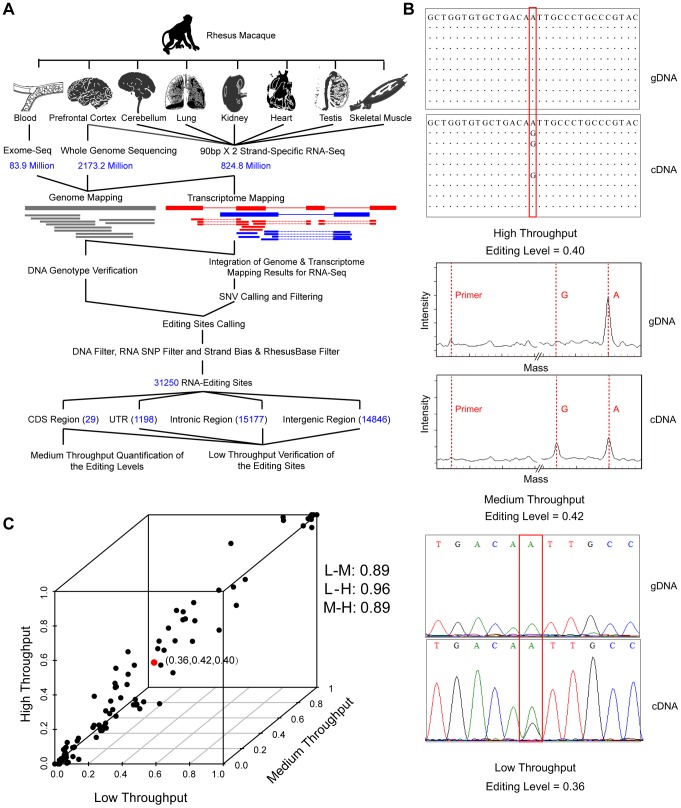
Genome-wide identification and verification of RNA editome in one rhesus macaque. (**A**) Overview of the experimental design – genome-wide identification, and medium- or low-throughput verification of RNA-editing sites. (**B**) An example showing the genotyping results for the genomic DNA (gDNA) and cDNA (cDNA) of one verified RNA-editing site (chr11:5028364, *KCNA1*). The levels of RNA editing were estimated from high-throughput, medium-throughput and low-scale data on the basis of read number, signal intensity contrast and peak height ratio between the edited and wild-type alleles, respectively. The primer peak and the genotype peak on mass spectrum are indicated by dotted lines in red. (**C**) Comparison of the levels of RNA editing estimated by high-throughput (H), medium-throughput (M) and low-scale (L) platforms. The example in (**B**) is highlighted in red. Pearson correlation coefficients between different platforms are shown on the right.

We performed a genome-wide study to identify RNA-editing sites in rhesus macaque (**Figure 1A**). Considering the error-prone gene structures in this species, strand-specific poly(A)-positive RNA-Seq was performed (**Materials and Methods**). A total of 824.8 million 90-bp paired-end reads were obtained for seven macaque tissues (prefrontal cortex, cerebellum, muscle, kidney, heart, testis and lung) derived from the same animal, and mapped to the macaque genome with high quality (**Table 1**, **Figure S1**). As a reference, genomic DNA derived from prefrontal cortex of the same animal was sequenced; a total of 1,763.3 million 90-bp paired-end reads were uniquely mapped to the macaque genome, with 92.2% of the genomic regions successfully sequenced and 91.6% covered by at least ten DNA reads (**Table 1**, **Figure S2**). Such deep coverage of the genome and transcriptome in multiple tissues of the same animal provided an ideal dataset to profile the RNA editome in rhesus macaque (**Table 1**, **Figures 1A** **, S1 & S2**).

**Table 1:** Statistics of deep sequencing for one rhesus macaque.

Tissue Type	Total Reads	Length	Q20	GC Content	Uniquely-Aligned Reads			
					Genome Mapping		Transcriptome Mapping	
**Strand-specific mRNA-Seq**								
**Testis**	100.7M	90 bp×2	97.1%	48.8%	67.6M	67.1%	38.7M	38.4%
**Frontal Cortex**	142.1M	90 bp×2	97.0%	47.7%	95.2M	67.0%	60.8M	42.8%
**Muscle**	120.0M	90 bp×2	96.9%	49.8%	75.7M	63.1%	53.0M	44.2%
**Cerebellum**	129.0M	90 bp×2	96.4%	47.5%	92.5M	71.7%	42.6M	33.0%
**Lung**	113.6M	90 bp×2	96.8%	48.9%	84.1M	74.0%	35.0M	30.8%
**Heart**	123.7M	90 bp×2	97.1%	46.6%	72.9M	58.9%	72.2M	58.4%
**Kidney**	95.7M	90 bp×2	97.3%	48.0%	60.0M	62.7%	45.1M	47.1%
**Whole Genome Sequencing**								
**Frontal Cortex**	2173.2M	90 bp×2	94.3%	41.7%	1763.3M	81.1%	-	-
**Whole Exome Sequencing**								
**Blood**	83.9M	90 bp×2	95.9%	49.1%	75.2M	89.6%	-	-

Stringent computational pipelines were then developed to place the DNA reads and cDNA reads to the macaque genome, especially for the definition of uniquely-mapped cDNA reads (**Materials and Methods**). Briefly, one cDNA read was considered to be uniquely mapped only if it had no second-best hit or the second-best hit included at least three additional sequence alignment mismatches, when considering both the genome and the transcriptome mapping models. The technical issues raised recently [8]–[10], such as systematic sequencing errors as well as pseudogene- or paralog-related misalignments in short-read processing, were thus adequately addressed. Based on uniquely-mapped reads, candidate RNA-editing sites were identified by distinguishing sequence discrepancies between DNA and cDNA. This initial list was further subjected to stringent inclusion criteria to control for false-positives (**Figure 1A**). Briefly, following previous large-scale studies in human [3], [6], [7], [12], a standard computational pipeline with multiple filters was introduced to eliminate false-positives due to amplification bias, sequencing errors and mapping errors. To account for the error-prone gene structures in rhesus macaque, we introduced one additional filter to remove editing sites located in previously mis-annotated transcripts, on the basis of in-house revised transcript structures [19], [20] (**Figures 1A** **&** **2B** **;** **Materials and Methods**). Particularly, considering the less stringent requirement for accurately calling the widespread editing sites in *Alu* regions compared with those in non-*Alu* regions [21], we introduced more relaxed criteria for identifying editing sites in *Alu* regions (**Materials and Methods**). Overall, 31,250 macaque editing sites were identified, with 29 in coding regions, 1,198 in untranslated regions, 15,177 in intronic regions and 14,846 in intergenic regions (**Figure 1A** **and Table S1;** **Materials and Methods**). Similar to the reports in human [21], the vast majority (30,699 of 31,250) of these sites are located in *Alu* repeat elements.

**Figure 2:**
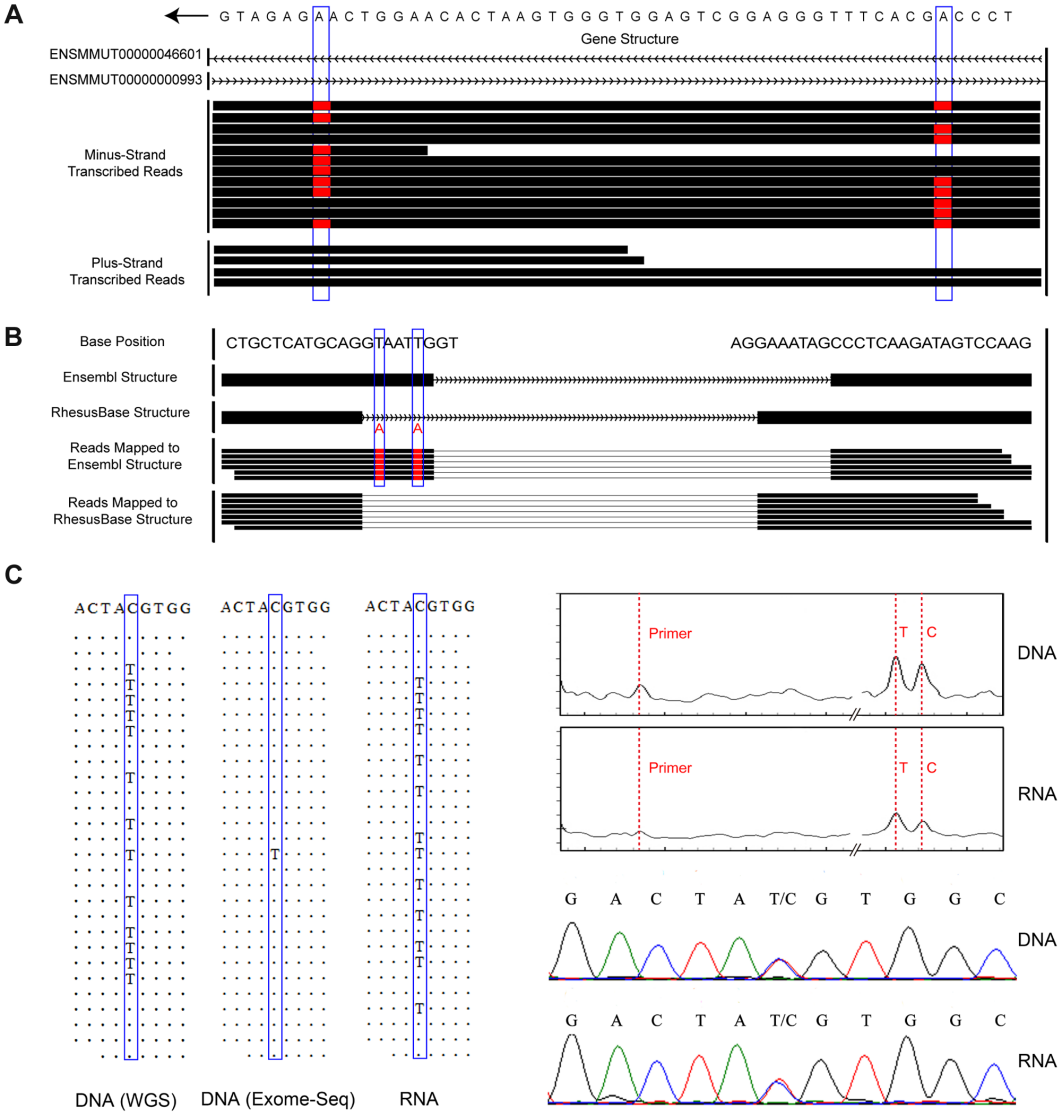
Experimental and computational strategies for accurate editome identification in rhesus macaque. Potential false-positives in the RNA editing calling workflow were minimized by a more thorough design in our pipeline strategy. (**A**) Two discrepancies between RNA and genomic-DNA sequences (highlighted by blue boxes) were located in a *cis*-natural antisense region where both DNA strands could be transcribed. Strand-specific RNA-Seq clearly distinguished the sequence reads transcribed from the two strands and correctly assigned this site as A-to-G editing, as no discrepancy was detected in the plus-strand transcribed gene. (**B**) Based on the macaque gene structures defined in-house (**RhesusBase Structure**), one of the exon-intron boundaries of *ENSMMUT00000021567* was incorrectly defined by a previous annotation (**Ensembl Structure**). Two T-to-A DNA-RNA discrepancies highlighted by blue boxes would be incorrectly identified as T-to-A RNA editing with the RNA-Seq reads being aligned to the mis-annotated transcript structure. (**C**) The genotype of the site highlighted in the blue boxes was incorrectly recognized as homozygous in DNA and heterozygous in RNA, since only 1 out of 28 sequence reads supported the mutant allele T in DNA, leading to incorrect assignment of a C-to-T editing event. Both Sequenom mass array and Sanger sequencing validations excluded such false-positives, which may arise due to low sequencing coverage and biased allele capture efficiency in the exome-Seq assay.

### An accurate, quantitative and representative catalog of RNA-editing sites across rhesus macaque transcriptome

We next set out to confirm that these sites represent *bona fide* RNA-editing events rather than technical artifacts. Twenty-eight of all 29 editing sites (96.6%) in coding regions (**Figure S3,** **Tables 2** **& S2**), as well as 77 of the 79 randomly selected sites (97.5%) in untranslated, intronic and intergenic regions (**Figure S4, Table S2**) were experimentally verified by PCR amplification and Sanger sequencing of both DNA (median length of the PCR products, 449 bp) and the corresponding cDNA (median, 449 bp). The validation rates for both coding and non-coding regions suggested that most of the sites identified in this genome-wide study should be verifiable.

**Table 2:** 28 verified editing sites in the macaque coding regions.

Position	Form	Host Gene	Recoding Type	Function of the Host Gene
**1:138950859**	A→G	COPA	I→V non-synonymous change	nucleoside-triphosphatase regulator, ion binding, protein binding
**6:129544096**	A→G	ARIH2	K→K synonymous change	ion binding, nucleic acid binding, protein binding
**6:38781096**	A→G	RICTOR	R→G non-synonymous change	protein binding
**7:89331048**	A→G	NOVA1	S→G non-synonymous change, protein stability [51]	nucleic acid binding, protein binding
**X:150275345**	A→G	GABRA3	I→M non-synonymous change, trafficking [52]	ion binding, protein binding, substrate-specific & transmembrane transporter, neurotransmitter binding, signal transducer
**10:26945910**	A→G	BLCAP	Y→C non-synonymous change, cancer biomarker [53]	-
**10:26945919**	A→G	BLCAP	Q→R non-synonymous change, cancer biomarker [53]	-
**10:26945949**	A→G	BLCAP	K→R non-synonymous change, cancer biomarker [53]	-
**11:5028364**	A→G	KCNA1	affinity for blocking particle, kinetics of channel inactivation [34]	ion binding, protein binding, substrate-specific & transmembrane transporter
**12:73754116**	A→G	UNC80	S→G non-synonymous change	-
**17:24407704**	A→G	COG3	I→V non-synonymous change	protein binding, substrate-specific transporter
**5:72269698**	A→G	IGFBP7	K→R non-synonymous change, proteolytic cleavage [54], physiological properties [23]	protein binding
**6:153684514**	A→G	CYFIP2	K→E non-synonymous change, biomarker for ALS [55]	protein binding
**X:152404069**	A→G	FLNA	Q→R non-synonymous change, physiological properties [23]	protein binding, signal transducer
**11:47207718**	A→G	ASIC1	T→A non-synonymous change	-
**7:54257914**	A→G	NEIL1	K→R non-synonymous change, nucleotide removal efficiency [36]	ion binding, nucleic acid binding, protein binding, hydrolase
**7:54257915**	A→G	NEIL1	K→K synonymous change, nucleotide removal efficiency [36]	ion binding, nucleic acid binding, protein binding, hydrolase
**5:149561914**	A→G	GRIA2	Q→R non-synonymous change, Ca-permeability [56], channel trafficking [57], receptor assembly [58]	protein binding, substrate-specific & transmembrane transporter, signal transducer
**5:149561918**	A→G	GRIA2	Q→Q synonymous change	protein binding, substrate-specific & transmembrane transporter, signal transducer
**1:134852231**	A→G	SMG5	R→G non-synonymous change	protein binding
**3:13010093**	A→G	SON	L→L synonymous change	nucleic acid binding, protein binding
**7:43601461**	A→G	PDCD7	Q→R non-synonymous change	-
**2:78236903**	A→G	FLNB	M→V non-synonymous change	protein binding
**4:33833717**	A→G	GRM4	Q→R non-synonymous change	metabotropic glutamate, GABA-B-like receptor, signal transducer
**4: 44072645**	A→G	TMEM63B	Q→R non-synonymous change	-
**8:9841193**	A→G	XKR6	R→G non-synonymous change	-
**5:149585132**	A→G	GRIA2	R→G non-synonymous change	protein binding, substrate-specific & transmembrane transporter, signal transducer
**X:148223918**	C→T	NOL11	T→I non-synonymous change	-

In addition to the traditional approach of PCR amplification and Sanger sequencing, we also performed a medium-throughput study to quantify the levels of coding region-associated RNA editing in the seven tissues using a mass array-based genotyping platform (**Materials and Methods**). The levels of RNA editing were then estimated and compared between the high-throughput, medium-throughput and low-scale assays (**Figure 1B**). Strikingly, the levels of RNA editing estimated by high-throughput technology were in close agreement with those by the other two independent platforms, particularly for sites with ≥10 supporting reads (the Pearson correlation coefficients were 0.89, 0.96 and 0.89; **Figure 1C**). This adequacy of the NGS data in estimation of RNA-editing levels thus indicated that quantitative characterization of the RNA editomes, particularly among tissues, individuals, and species, may be based on integrating in-house RNA-Seq data with public transcriptome data (**Figure S5**).

As stringent cutoffs for the sequencing depth of genome were instituted to distinguish RNA editing from systematic sequencing errors, allele-specific expression and duplication-related polymorphisms, we evaluated whether such a rigorous approach may have hampered the site-calling sensitivity in this study. Focusing on coding regions, we increased the coverage of genomic DNA sequences to 115-fold through an established whole-exome capturing and sequencing strategy [3] (**Table 1**). A total of 83.9 million DNA reads were then obtained and mapped to the macaque genome, with 96.9% of the coding regions being sequenced with high coverage (**Figure S2**). However, only six additional RNA-editing sites were identified using this targeted genomic reference, but were subsequently discarded by Sanger validation. These false-positives might have arisen largely due to biased capture efficiency in the exome sequencing assay favoring the wild-type allele (**Figure 2C**). Actually, even considering cross-species differences, the majority (13 out of 14) of those well-characterized human RNA editing sites as summarized by *Li et al* [4] were included in the initial list (**Table S3**), suggesting the high calling sensitivity of editing sites in coding region.

However, considering the coding regions are less repetitive and well-annotated than other genomic regions, it is not straightforward to generalize the high calling sensitivity in coding region to other genomic regions. Notably, the overall number of macaque editing sites we identified is lower than that in human, in which 84,750 editing sites were identified from poly(A)-positive RNA-Seq data (**Supplementary Table 1** in Reference [7], [21]). Although multiple factors, such as the differences in experimental design and the inherent difference in genetic makeup, may contribute to the human-macaque difference [7], [21] (**Discussion**), it is likely that false-negatives in RNA-editing detection could still result from our stringent criteria (**Materials and Methods** **,** **Discussion**). Nonetheless, such rigorous approach is necessary for controlling false-positives, especially considering the poor genome annotations and error-prone gene structures in rhesus macaque [19], [20], [22]. Importantly, despite the notion that certain degrees of false-negatives exist, this dataset may still represent a representative list of macaque editing sites for further interrogation of some global attributes of the RNA editome.

### Association of *ADARs*-mediated reactions with the macaque editome

Having established the feasibility of our experimental design and the authenticity of the macaque editing dataset, we next aimed to characterize the relevant molecular factors underlying the macaque RNA editome. To this end, several global attributes of the editome were first identified as follows.

**Figure 3:**
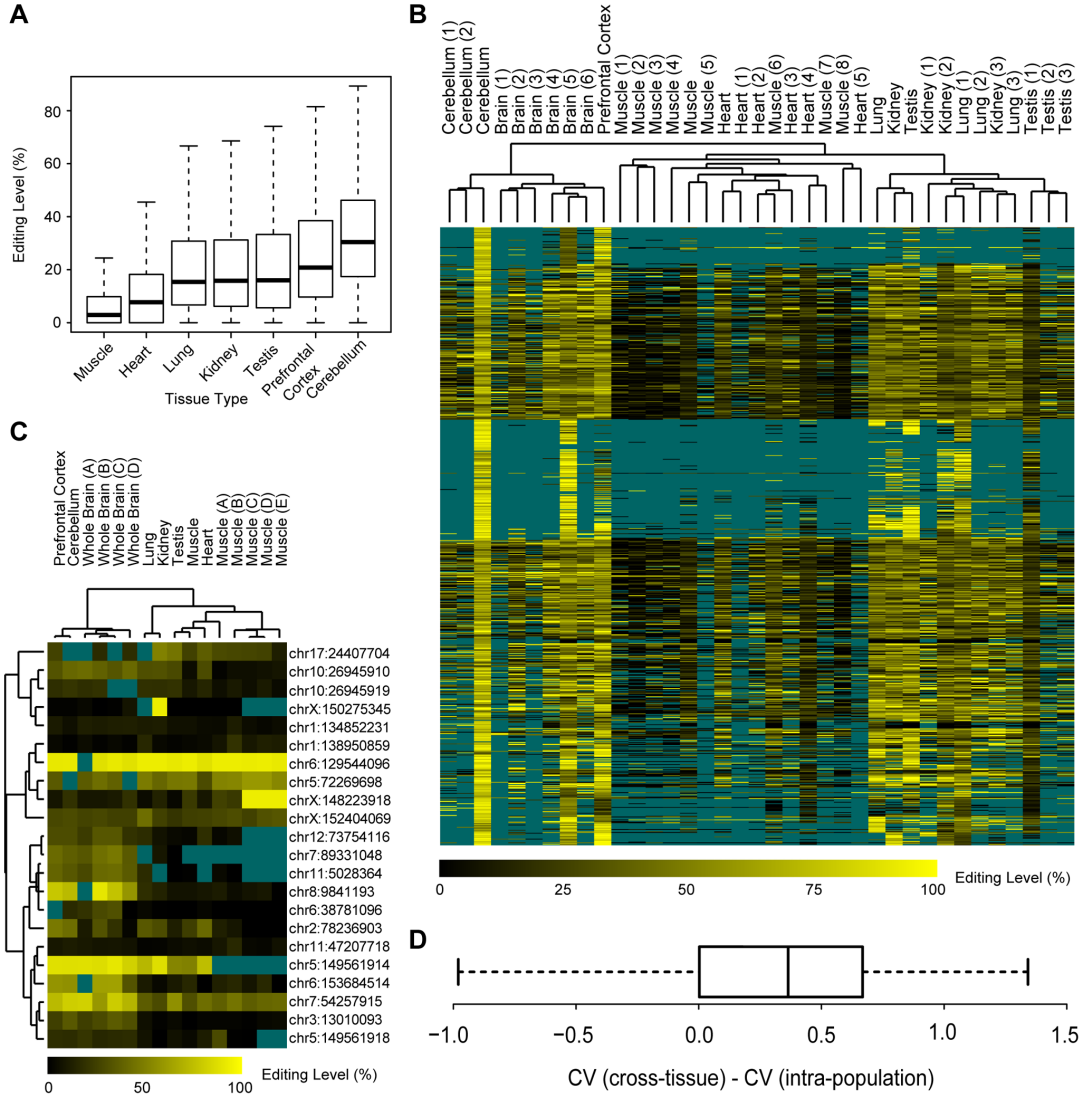
Characteristics of the rhesus macaque editome. (**A**) For editing sites in each type of tissue, the distribution of the levels of RNA editing was shown in boxplot. (**B**) Hierarchical clustering of editing levels of all editing sites across multiple macaque tissues and animals. Editing levels were estimated on the basis of RNA-Seq data in this study (Testis, Lung, Kidney, Heart, Muscle, Prefrontal cortex) and other public RNA-Seq data [Brain (1–6), Cerebellum (1–2), Muscle (1–8), Heart (1–5), Kidney (1–3), Lung (1–3), Testis (1–3)], with missing data shown in dark cyan. (**C**) Hierarchical clustering of editing levels is shown for selected RNA editing sites located in coding regions. Editing levels were estimated on the basis of mass array-based genotyping in seven macaque tissues derived from the same macaque (Testis, Lung, Kidney, Heart, Muscle, Cerebellum, Prefrontal Cortex), as well as five muscle and four brain samples obtained from different macaque animals [Muscles (A–E), Whole Brains (A–D)], with missing data shown in dark cyan. (**D**) The distribution of pair-wise comparison of intra-population and cross-tissue coefficient of variance (CV) values is shown in boxplot.

First, contrary to the previous study reporting all twelve possible forms for RNA-editing sites in human with a large proportion of transversions (43%) [5], we found that nucleotide transitions accounted for 99.9% of the editing sites in the macaque editome. Furthermore, 99.8% of the identified changes converted A to G, which is presumably a consequence of *ADAR*-mediated enzymatic reactions (**Figure S6**). We noted that the fraction of A-to-G transitions increased when more stringent filters were incorporated, from 65.6% in the initial list to 99.8% in the final list (**Figure S6**), suggesting that most of the nucleotide changes of the transversion type may have been due to technical artifacts [5], [8]–[10], rather than unknown mechanisms as proposed previously [5]. Second, the identified sites exhibited considerable variance in editing levels, with the median level ranging from 2.9% in muscle to 30.4% in cerebellum (**Figure 3A**), indicating a differential regulation profile similar to that reported in human [4]. Third, tissue profiling also revealed higher levels of RNA editing in the brain than in other tissues (**Figure 3A**), affirming a layer of regulation underlying the complex brain development in primates [23]–[25].

In addition, when comparing editing levels across tissues and individuals by integrating the in-house RNA-Seq data with public macaque transcriptome data, we further found smaller intra-population variations of the editing levels in comparison with cross-tissue variations as revealed by hierarchical clustering analyses (**Materials and Methods** **;** **Figure 3B** **, Table S4**). As the editing levels were estimated according to RNA-Seq data where the estimation might be less accurate for sites with lower sequencing coverage, we further used a mass array-based genotyping platform to quantify the levels of editing in coding regions of RNA from the seven original macaque tissues and nine additional samples (**Materials and Methods**). The mass array data further verified the intra-population conservation of the macaque editome (**Figure 3C**). Besides these qualitative clustering analyses, we further measured the coefficients of variation (CV) of editing levels across different animals, as well as across different tissues from the same animal (**Materials and Methods**). As expected, for most editing sites (93.4%), the intra-population standard deviation of editing levels was smaller compared to the average editing level (**Figure S7A**). In addition, the variability across macaque animals is significantly lower than that across tissues, as indicated by the pair-wise CV comparisons (*Wilcoxon one-tail test*, *p-value* = 4.2e-6; **Figure 3D**). Our findings therefore demonstrated that, similar to other fundamental gene regulation mechanisms [26], [27], there may be a regulatory commonality of RNA editing within populations, in accordance with a previous study on candidate genes [15].

**Figure 4:**
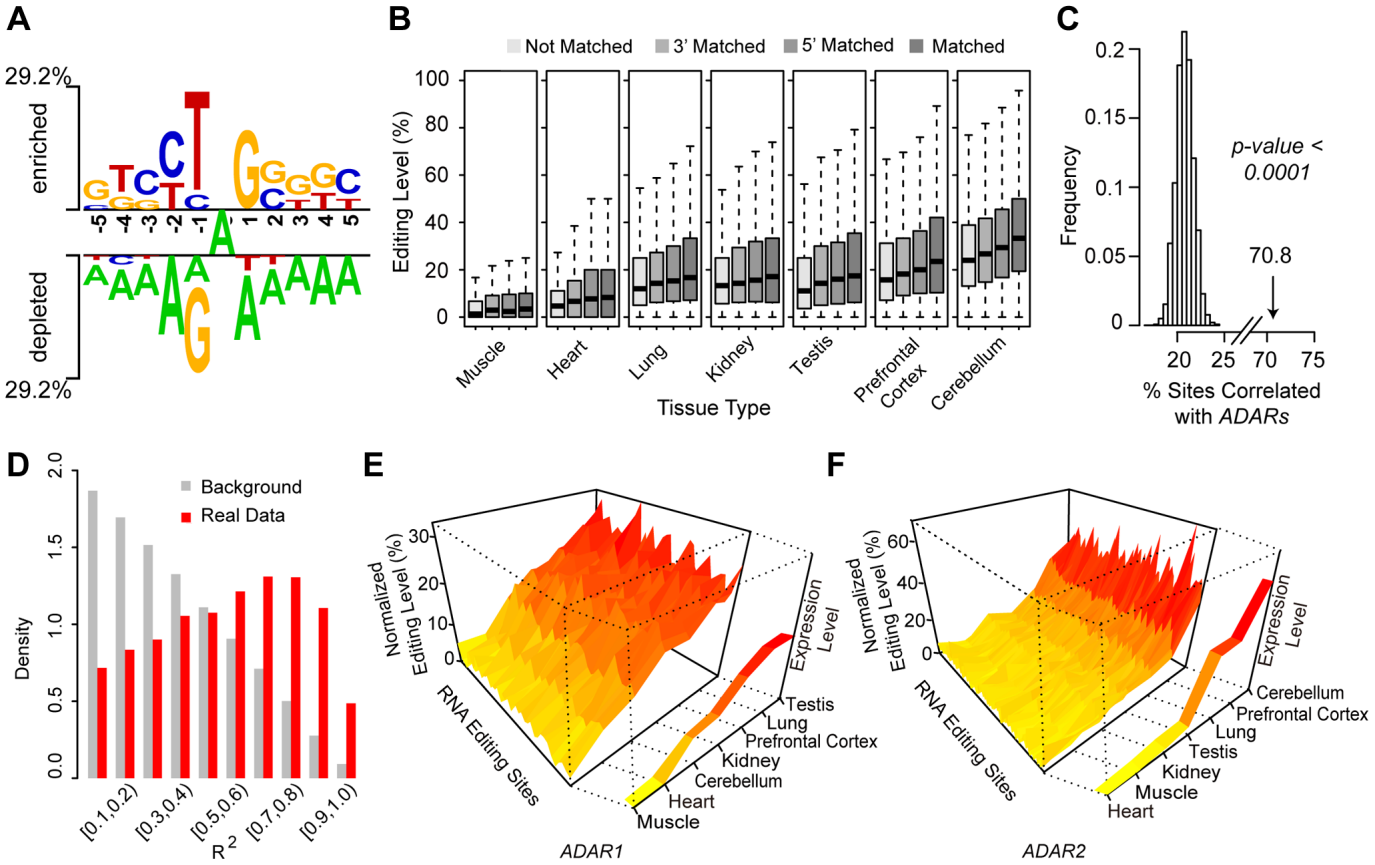
*ADARs*-mediated enzymatic reactions is associated with the macaque editome. (**A**) The enriched (above the top line) and depleted (below the bottom line) nucleotides nearby the focal editing sites are displayed in Two-Sample Logo, with the level of preference/depletion shown in height proportional to the scale. (**B**) The editing sites were divided into four categories on the basis of the local sequence context nearby the editing site, as described in **Materials and Methods**. For each category, levels of RNA editing are shown in boxplots according to the tissue types. (**C**) Distribution of the percentages of editing sites showing tissue distribution of editing levels positively correlated with the expression of *ADARs* (Spearman's rank correlation coefficient at ≥0.5), for 10,000 permutation datasets neglecting tissue relationships for the tissue expression profile. The percentage for the real data was indicated by the arrow with *Monte Carlo p-value*. (**D**) Distributions of *R^2^* values in models assuming association of editing level with *ADARs* expression are shown as the **Real Data**, as well as the **Background**, which correspond to randomly shuffled profiles. (**E, F**) The tissue expression profiles of *ADAR1* or *ADAR2* were ordered based on RNA expression levels, and normalized editing levels of A-to-G sites were aligned accordingly. These A-to-G editing sites showed similar trends in the distribution of editing levels along the ordered tissue expression profile of *ADAR1* (**E**) or *ADAR2* (**F**).

We next investigated the relevant molecular factors underlying the macaque editome, and subsequently made the following observations. First, the sequence context of the overwhelmingly-represented A-to-G editing sites verified the known attributes of *ADAR* substrates, in that the nucleotide 5′ to the editing site significantly disfavored G, while the 3′ nucleotide favored G [28] (**Figure 4A**). In any given tissue, it seems that the local sequence context flanking the editing sites is a relevant factor for the global editing levels – sites with a matched *ADAR* recognition motif usually showed significantly higher editing levels than those with a partially-matched or non-matched recognition motif (*Wilcoxon rank test*, *p-values* shown in **Table S5**, **Figure 4B**). Particularly, 5′ nucleotide seemed to be more determinative as sites with 5′ matched motif usually showed significantly higher editing levels than those with 3′ matched motif only, a finding consistent with previous reports [29], [30] (**Figure 4B** **, Table S5**). However, quantitative analyses with a Triplet model as previously described [29] revealed that only a small proportion of the site-to-site variance could be explained by the nearby sequence motif (**Materials and Methods**). We suspect that some confounding factors, such as substrate-specific variations and quantitative accuracy of editing level by RNA-Seq, might partially contribute to the low prediction power: when investigating one RNA substrate harboring 15 editing sites with the editing levels estimated according to the Sanger sequencing data, where these confounding issues were controlled, 52.4% site-to-site variances could be explained by sequence motif (**Figure S4**), a proportion comparable to a previous study using fixed RNA substrate and peak-based editing level estimation [29].

Especially, we noted a quantitative correspondence of the tissue-biased profile of the RNA editome to the tissue expression profile of *ADARs*, although previous studies in rodents did not detect a significant correlation [13], [14]. First, on the basis of a test for Spearman's rank correlation, 70.8% of the A-to-G macaque editing sites showed a tissue distribution of editing levels positively correlated with the expression of *ADARs* (Spearman's rank correlation coefficient ≥0.5), such an observation represents a statistically significant excess of editing sites with positive correlations (*Monte Carlo p-value*<0.0001; **Figure 4C**; **Materials and Methods**). Second, to further provide a quantitative estimate, we performed linear regression analysis to illustrate the association of *ADAR* expression profiles with the editing levels (**Table S6**; **Materials and Methods**). To this end, the *R^2^* was used as a quantitative indicator for the proportion of the variance of editing level that may be explained by *ADAR* expression profile (**Materials and Methods**). Compared with the distribution of *R^2^* values on randomly shuffled profiles neglecting tissue relationships for the tissue expression profile, the detected distribution of the correlations between the cross-tissue variance of editing levels and *ADARs* expression could hardly be explained by random permutations (*Monte Carlo p-value*<0.0001; **Figure 4D**), indicating that *ADAR* expression levels are indeed a relevant factor in determining global editing levels (**Figure 4D**). In addition, according to the regression analyses, we further found that 209 of these sites (10.7%) were significantly correlated with *ADAR1* only, 567 sites (29.0%) with *ADAR2*, and 31 sites (1.6%) with both *ADARs* (**Table S6**; **Materials and Methods**). For these sites, the distributions of editing levels across seven tissues were shown, which were closely commensurate with the tissue expression profiles of *ADARs* (**Figure 4E & F**). After multiple testing corrections, 381 sites (19.5%) still showed significant positive correlation in tissue distribution between RNA editing level and the expression of *ADARs* (**Table S6;** **Materials and Methods**).

Overall, our qualitative and quantitative data demonstrated that the intra-population variability of editing levels is significantly lower than that across tissues, and that both the *ADAR* expression profile and the local sequence context are relevant factors in determining global editing levels. Furthermore, these findings are consistent for sites located in different genomic regions, such as *Alu vs* non-*Alu* regions (**Figures S8 & S9**).

### Evidence of purifying selection on the editome landscape

**Figure 5:**
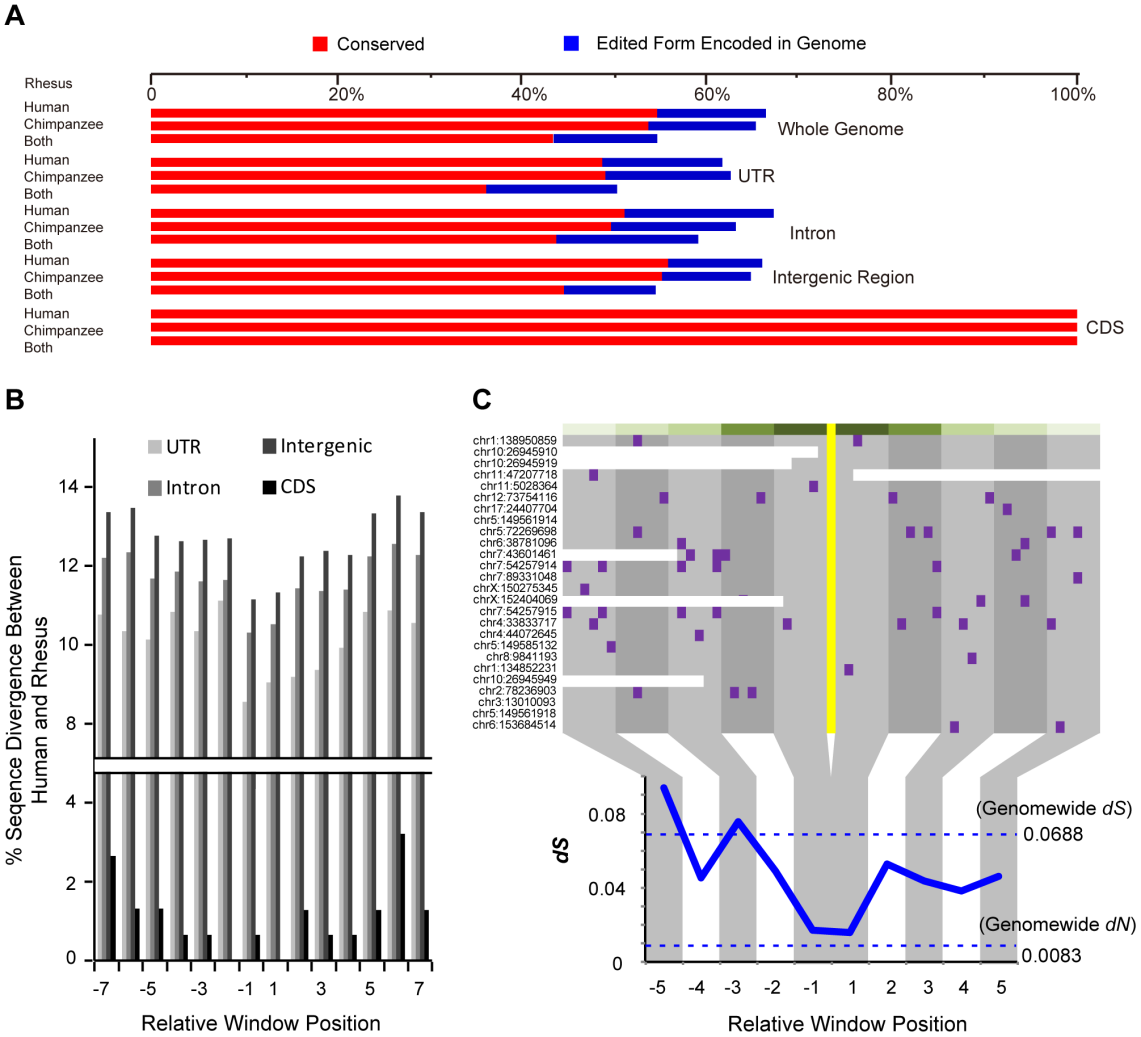
Contribution of purifying selection to the RNA editome in primates. (**A**) The percentages of macaque editing sites with corresponding editing sites in human and/or chimpanzee (red bars), or genomically encoded in the two species (blue bars), are shown for the total editome (top), or for editing sites in different genomic regions (bottom). (**B**) The genomic sequences nearby the macaque editing sites were compiled according to the distances to the editing sites. For each 6-nucleotide window, the proportion of divergent sites between human and rhesus macaque are shown for different genomic categories. (**C**) Distribution of human-macaque synonymous divergent sites nearby the A-to-G editing sites. The codons with RNA-editing sites are highlighted in yellow and each synonymous divergent site in purple. The distribution of synonymous divergence (*dS*) values near the RNA-editing site, calculated using a 6-codon window, is shown in the lower panel, with the genome-wide *dN* and *dS* between human and rhesus macaque indicated by the dotted line.

With the spectrum of macaque editing sites, we next performed a comparative analysis to examine whether the editing sites we identified in rhesus macaque could also be detected in human and chimpanzee orthologous regions. To this end, we integrated public available RNA-Seq data in human or chimpanzee to trace the orthologous regions of macaque editing sites (**Table S4**). For the 1,111 macaque editing sites with homology in both of these transcriptomes with adequate cDNA coverage (**Materials and Methods**), 599 (or 53.9%) and 590 sites (or 53.1%) could also be detected in human or chimpanzee, respectively, with 476 sites (or 42.8%) detectable in all three species (**Figure 5A**). Such extent of overlap was significantly higher than the background, which was calculated using the adjacent non-edited sites to indicate the degree of RNA-Seq sequencing errors (*Chi-square test*, *p-value*<2.2e-16, **Figures 5A** **& S9**). Some macaque editing sites (138 sites, 12.4%) were found with the edited forms encoded in human or chimpanzee genome (**Figure 5A**), an observation in line with previous studies on several human candidate genes [31], [32]. Compared with other genomic regions, RNA-editing events in coding regions showed a particularly higher degree of parallels across the three species, in that, all of these macaque editing sites could also be detected in human and chimpanzee homologous regions (**Figure 5A**).

On the basis of these clues, we further tested whether the cross-species similarities in RNA editome were maintained by purifying selection due to the functional implications of these regulations, or simply due to the relatively lower sequence divergences among these closely related primate species. As shown above, the local sequence context of the editing site was important in RNA-editing regulation, as it may be implicated in the formation of a suitable *ADAR* substrate structure [28]. Therefore, primate-specific purifying selection nearby the editing site would presumably be an evidence for the functional relevance of the RNA-editing events. When examining the distribution of diverged sites between human and rhesus macaque, we discovered substitution valley of decreased divergence flanking the editing sites, as compared with the more distal regions as background (**Figures 5B** **& S11;** **Materials and Methods**). As a control, for macaque editing sites encoded in both human and chimpanzee with other types of nucleotides, no decreased divergence was observed nearby the focal editing sites in sequence comparison between human and chimpanzee (**Figure S11C**). Further analysis revealed little effect of expression levels of the host genes on the signatures of substitution valley of decreased divergence (**Figure S11E, F & G;** **Materials and Methods**). Overall, the divergence rates dropped by 15.2%, 12.3% and 13.0% for RNA-editing sites located in untranslated, intronic and intergenic regions, respectively, in contrast to 74.0% in the coding regions. The stronger selective constraint detected on coding regions recapitulates the particularly higher degree of parallels of editome in coding regions, as compared with other genomic regions (**Figure 5**). Due to limited number of editing sites in coding regions, we performed *Monte Carlo* simulation with random sampling of coding regions across the macaque genome to test whether the detected divergence rate drop was an effect of sampling bias on limited observations. The result revealed that such a possibility is rare (*Monte Carlo p-value* = 0.005).

For RNA-editing sites in coding regions, we also examined the distribution of synonymous divergent sites between human and rhesus macaque surrounding the editing sites. Although synonymous sites have been considered to be largely neutral [33], we noted that their presence near the editing sites was actually more selectively constrained than distant synonymous sites [23], [34] (**Figure 5C**). The synonymous substitution rate nearby the editing site was nearly equivalent to the genome-wide substitution rate of nonsynonymous sites that is under strong purifying selection (**Figure 5C** **;** **Materials and Methods**). We further noted that the genes regulated by these recoding RNA-editing events were significantly enriched in the functional category of biological binding [35] (*Hypergeometric test, p-value* = 1.7e-3). Among these sites, three were located in orthologous proteins in human with solved crystal structures – the nucleotide/codon re-assignment by RNA editing reportedly regulate the activity of the voltage-gated potassium channel [34] or the efficiency of DNA glycosylases in the removal of damaged nucleotides [36]. These RNA-editing events shaped by purifying selection may thus represent a form of functional regulation that underlies processes associated with protein, ion and nucleic acid binding.

Taken together, the dampened divergence rate or synonymous substitution rate around the editing sites reflects the evolutionary necessity of retaining some of these editing substrates with their double-stranded structure (**Figure 5**). However, in contrast to the sites in coding regions, it is possible that a smaller proportion of functional RNA editing sites exist in these non-coding genomic regions, considering the weaker selective constraints detected (**Figure 5A & B**). These findings thus complement the “continuous probing” model postulating a tinkering-based origination of functional editing sites [17], [37] (**Discussion**).

## Discussion

### Macaque editome identification: Experimental and computational strategies

Despite extensive efforts and discussion, accurate definition of the human editome by using NGS data alone remains technically challenging [5], [8]–[12]. The widespread human editing sites detected in a recent study might have been largely a result of technical artifacts, such as systematic sequencing errors and flaws in the subsequent computational analyses [5], [8]–[12]. Other studies introduced more stringent pipelines to control for high false-positive rate, such as by sequencing only target regions with putative RNA-editing sites [4], by removing RNA-editing sites on repetitive genomic regions [3], or by rejecting sites corresponding to genomic polymorphisms [12]. These approaches have significantly improved the accuracy of RNA editing site calling, but additional barriers still exist for unbiased and definite identification of editome on the genome-wide scale [18]. Particularly for species with poor genome annotations and error-prone gene structures such as the rhesus macaque [19], [22], successful editome detection is hampered by difficulties in accurately mapping RNA-Seq reads and discerning discrepancies.

In this study, beyond the filters established previously to remove potentially erroneous editing sites with i) low read coverage [6], [7], ii) poor base-calling quality or multiple types of variation [6], [7], iii) strand-biased cDNA read distributions [7], and iv) location in repeat genomic regions [3], [7], [12], we installed additional experimental designs and analytical measures with advantage in eliminating false-positives in our pipeline. First, all NGS assays were performed in macaque tissues derived from the same animal, which effectively excluded individual differences in the genome and transcriptome (**Materials and Methods**). Second, strand-specific RNA-Seq technique significantly controlled for potentially ambiguous calls due to the widespread *cis*-natural anti-sense expression [18], [38] (**Figure 2A**). Third, long paired-end reads were generated in our deep sequencing analysis, ensuring accurate mapping with sufficient sequencing depth on >18,000 mRNAs [39] and 96.9% of macaque genomic regions. Fourth, a more stringent read mapping strategy was applied to facilitate the definition of uniquely-mapped reads (**Materials and Methods**), which efficiently diminished false mapping due to processed pseudogenes [3]. In addition, considering the error-prone gene structure annotations for rhesus macaque [19], [20], we further introduced inclusion criteria to remove editing sites located in previously mis-annotated macaque transcripts. Taken together, as we demonstrated above, these efforts ensured the identification of an accurate and quantitative catalog of RNA-editing sites.

However, the stringent criteria we used to control for the false-positives would cause some false-negatives in RNA-editing detection, although the calling sensitivity in coding region was proved to be good (**Figures 1** **&** **2**). To evaluate the degrees of false-negatives of our stringent computational pipelines, we applied the identical pipeline and inclusion criteria on human poly(A)-positive RNA-Seq data reported previously to identify human editing sites [7], [21]. Compared with the original poly(A)-positive RNA-Seq study reporting 84,750 human editing sites (see **Supplementary Table 1** in Reference [7], [21]), 20,065 editing sites were identified by our pipeline, with A-to-G transitions accounted for 94.3% of the identified editing sites. Considering the total sequencing depth of this human study is much lower than that of our study in rhesus macaque, we slightly modified our inclusion criteria for RNA-editing sites by decreasing from five to two the minimum RNA-Seq reads required to support the variant form (while keeping all other parameters used in sequence alignment and single-nucleotide variation calling), and consequently identified 80,375 editing sites, a number comparable with the original report in human (84,750 sites; **Materials and Methods**) [21]. It is obvious that more macaque editing sites would be expected, especially when increasing the sequencing depth of the transcriptome. However, with the experimental efforts in minimizing the effects of computational detection sensitivity in our study, such as the significantly elevated transcriptome sequencing depth in rhesus macaque to increase the detection power of variants, and the strand-specific, long paired-end reads designed to increase the proportions of uniquely-mapped reads (**Materials and Methods**), it is likely that some non-technical factors, *e.g.*, the inherent difference in genetic makeup for the inverted *Alu* pairs, may also contribute to this human-macaque difference [4], [7], [40], [41].

Viewed together, although our rigorous experimental and computational paradigm would cause some false-negatives, it would be a necessary compensation for an accurate and quantitative catalog of RNA-editing sites in rhesus macaque, considering the poor genome annotations and error-prone gene structures in rhesus macaque [19], [20], [22]. Importantly, the catalog represents a representative account of RNA-editing sites across rhesus macaque genome for further interrogation of the global attributes of the RNA editome.

### Characteristics and implications of the macaque editome

Aside from the technical issues, the present work on the macaque RNA editome provided novel insights into several aspects of the RNA editing process. First, large-scale sequencing on a broad range of tissue samples from the same or different animals allowed for a comparative editome analysis. We subsequently deduced from such a study that, while there is large degree of variance between sites and tissues (**Figure 3A**), the intra-population variability of editing levels is significantly lower than that across tissues, suggesting a regulatory commonality of RNA editing within populations similar to other fundamental gene regulation mechanisms [26], [27] (**Figures 3B, C, D** **, S7 & S8**). Second, the global attributes of editing were further verified and quantified to show that the occurrence of RNA editing is correlated with the flanking sequence signatures, as well as the levels of *ADARs* expression (**Figure 4**). The macaque editome is thus partially associated with *ADARs*-mediated enzymatic reactions, and the *cis*- and *trans*-directed mechanisms associate with *ADARs*, such as the chemical affinity of *ADAR* binding sites and *ADARs* concentration, are thus likely to be relevant with the regulation of the macaque editome.

### RNA-editing regulation: Functional outcome and significance

While hereditary information is modified by RNA editing, evidence for functional significance of this process is largely lacking thus far [17]. Although functional RNA-editing sites have been sporadically reported, they may represent only isolated cases rather than a general mode of regulation. In this study, with an accurate and informative editome defined across multiple tissues and animals, we found some intra-population conservation of the macaque editome, as well as some parallels of the editome across multiple primate species (**Figures 3** **,** **4** **&** **5**). However, our findings also suggest that the editome is partially associated with *ADARs*-mediated enzymatic reactions. It is thus possible that sites showing high affinity to *ADARs* in one macaque animal would also have high affinity to *ADARs* in other macaque animals, or in humans and chimpanzees, considering the relatively lower sequence divergences among these closely related primate species. To this end, we tested whether the cross-species similarities in RNA editome were maintained by purifying selection due to the functional implications of these regulations, or simply due to such a passive mechanism. Interestingly, in support of the functional relevance of some of these editing sites, substitution valley of decreased divergence was detected around the editing site (**Figure 5**), suggesting the evolutionary necessity of retaining some of these editing substrates with their double-stranded structure. Taken together, the findings on the population-wide and evolutionary conservation of the macaque editome, as well as the contribution of purifying selection to editome shaping, lend support to the functional significance of this co-transcriptional regulation as a whole.

Interestingly, when investigating the dampened divergence rate for editing sites across different genomic regions, stronger selective constraint was detected on coding regions, while sites in other regions also showed some degrees of weaker evolutionary constraints (**Figure 5A & B**). This analysis implies that, in contrast to the sites in coding regions, a smaller proportion of functional RNA editing sites exist in non-coding genomic regions (**Figure 5A & B**). The varied proportions of functional editing sites across different genomic regions thus support the “continuous probing” model postulating that most of the editing sites are neutral with low editing levels, acting as a selection pool for a few functional editing sites [17]. However, our findings also suggest that the RNA editing levels are partially associated with the chemical affinity of *ADAR* binding sites, as well as *ADAR* concentration. Thus, the editing levels are not necessarily low even for those potentially neutral editing sites (**Figure S12**), a notion that complements the “continuous probing” model by illustrating a clearer process for the tinkering-based origination of functional RNA editing sites [**17**], [**37**].

## Materials and Methods

### Ethics statement

Rhesus macaque tissue samples were obtained from the AAALAC-accredited (**A**ssociation for **A**ssessment and **A**ccreditation of **L**aboratory **A**nimal **C**are) animal facility at the Institute of Molecular Medicine in Peking University. Experiments with animals were done in accordance with protocols approved by the Institutional Animal Care and Use Committee of Peking University and followed good practice.

### Library preparation, sequencing, and quality control for RNA-Seq, genome resequencing and Exome-Seq

Strand-specific RNA-Seq libraries were prepared from seven rhesus macaque tissues derived from the same animal as previously reported [38], [39], [42]. Genomic DNA was obtained from prefrontal cortex and blood of the same animal for the library preparation of genome resequencing and parallel exome capture and sequencing, respectively. Exome fragments were captured and enriched using SureSelect Human All Exon 50 Mb kits (Agilent Technologies) following the manufacture's protocol. NGS was performed on a HiSeq 2000 Sequencing System, with a 90×2 paired-end read mode. Comprehensive evaluation of sequencing data was carried out using FastQC (v0.10.0), bedtools (v2.16.2), the Tophat (v1.2.0) package, and in-house Perl and R scripts. Overall, high-quality RNA-Seq data were obtained on the basis of high base quality (**Figure S1A**), unbiased read distribution on transcripts (**Figure S1B**), correct strand information (**Figure S1C**), little DNA contamination (**Figure S1D**), and a low and evenly-distributed mismatch rate across the reads (**Figure S1E**). The quality of genome resequencing and exome-Seq were also evaluated following the strategy reported in a previous study [3] (**Figure S2**). Deep sequencing data in this study are available at NCBI Gene Expression Omnibus and SRA under accession numbers GSE34426, GSE42857 and SRP039366.

### Sequence alignment and strategy for single-nucleotide variation calling and filtering

Exome-Seq or genome resequencing reads were aligned to the rhesus macaque genome (rheMac2) with BWA (v0.5.9-r16), from which only uniquely-mapped reads were retained. RNA-Seq reads were firstly mapped to the macaque genome (rheMac2) by BWA (v0.5.9-r16). Meanwhile, the RNA-Seq reads were also mapped to the transcriptome (Ensembl v65) by BWA (v0.5.9-r16) to address the technical issue of gapped alignment, as well as to control for false-positives derived from pseudogene- or paralog-related misalignments in short-read processing. Unique mapping of a RNA-Seq read was then defined as i) no more than four mismatches detected in the alignment to the genome or transcriptome reference; and ii) with no second-best hit or the second-best mapping model including no less than three additional alignment mismatches than the best model. For reads aligned to both the genome and the transcriptome, unique mapping of RNA-Seq reads was defined when combining the genomic mapping with the transcriptome mapping results, while other reads uniquely aligned only to the genome or the transcriptome were kept. Unique alignments were further examined to remove cases with incorrect read-pairing information [39]. This stringent mapping strategy effectively eliminated potential mis-alignment due to processed pseudogenes or un-annotated transcripts [3]. With this stringent mapping strategy, it is possible that the hyper-edited regions are under-represented, as their detection requires alignment procedures tolerating more mismatches and thus is different from most of the current editing detection schemes including ours [43].

Uniquely-mapped reads were first divided into two groups: reads transcribed from the plus-strand and those from the minus-strand. After removing redundant reads with identical start and end positions, single-nucleotide variation (SNV) calling was separately performed for the two groups of reads using Samtools (v0.1.16), with the parameter “samtools mpileup -C 50 -E –Q 25 –ug”. The special parameter “bcftools view -p 1” was set to keep SNVs significantly deviating from heterozygous allele distribution. Reads harboring SNVs within 5 bp of both ends were discarded in SNV calling due to read end-biased sequencing errors [8]–[10].

### Inclusion criteria for RNA-editing sites

Whole genome sequencing data were used to determine the genotype by adopting criteria similar to those of Li *et al* [4]. At least ten genomic reads were required to estimate the genotype of one site, and the corresponding genotype of a candidate RNA-editing site was required to be homozygous with >95% of the covered reads supporting the major allele type (**DNA Filter**, **Figure 1A**). RNA SNVs with a homozygous genotype were included in an initial list of RNA-editing sites, and were further subjected to a stringent filtering protocol: **i**) The **RNA SNP Filter**, in which at least five RNA-Seq reads, with ≥3 nucleotides sequenced with high PHRED base quality (≥25), were required to support the variant form, thus eliminating false-positives due to amplification bias or sequencing error (**Figure 1A**). SNVs displaying more than one mismatch type were discarded. For candidate RNA-editing sites in non-*Alu* regions, we performed BLAT alignment filtering to eliminate SNVs potentially caused by mis-alignment to paralogs or pseudogenes [3], [7], [12], [21]; **ii**) A **Strand Bias Filter** was also introduced as previously proposed [7]. Briefly, RNA-editing sites exhibiting strand bias in read distribution (*Fisher's exact test*, *p-value*<0.05), or supported by <2 reads on either of the two strands, were excluded (**Figure 1A**), while the minimal supporting reads required on each strand was decreased from two to one for candidate editing sites located in *Alu* regions. To evaluate the performance of this filter, we performed mass array-based genotyping to evaluate the removed sites in coding regions. This filter efficiently removed false positives at a low cost of identification sensitivity, in that 84.6% of the sites were indeed erroneous identification and six verified editing sites were included in the final list; **iii**) The **RhesusBase Filter**, in which we further checked the RNA-editing sites by mapping the raw reads containing these sites to refined transcript structures in the RhesusBase [19], [20], since some exon-intron boundaries have been mis-annotated previously [19], [20]. SNVs located within 5 bp of the splicing junctions were further manually curated, as alignment errors are more frequent around these junctions [8], [10]. The locations of editing sites were then defined on the basis of both Ensembl and RhesusBase gene annotations [19], [20]. Especially, considering the error-prone macaque gene models [19], [20], editing sites located in coding regions were defined only when the exon-intron structures were supported by at least one RNA-Seq junction reads or RefSeq gene models mapped from other species.

All candidate RNA-editing sites in coding regions that passed the above protocol, as well seventy-nine randomly selected RNA-editing sites in untranslated, intronic and intergenic regions, were further verified by PCR amplification and Sanger sequencing of both DNA and the corresponding RNA (**Figures S3 & S4,** **Tables 2** **& S2**). The sequence coverage of these sites ranges from 12 to more than 100 RNA-Seq reads, with the estimated editing levels from 3% to 100%. For editing sites in coding regions, we also performed mass array-based genotyping on all cDNA and the matched DNA samples on an iPLEX Gold MassARRAY system (Sequnom Inc.) to independently verify the RNA-editing sites and the corresponding editing levels. Primers were designed with MassARRAY assay design software. Amplification reactions, digestion of unincorporated dNTPs and MALDI-TOF mass spectrometry were performed in accordance with the manufacturer's instructions. Signal intensities for two alleles were automatically assigned followed by manual confirmation. Briefly, the genotype was assigned as the ratio of the area of ‘G’ signal to the area of both ‘G’ and ‘A’ signals if the editing form was A-to-G, and ideally a ratio of 0 represented homozygous A/A while 1 represented homozygous G/G. Considering the noise in the Sequenom mass array platform [44], a candidate RNA-editing site was confirmed when the ratio of edited form was ≥0.10 in at least one of the seven cDNA samples derived from macaque tissues, and <0.10 in the DNA samples.

To further assess the degrees of false-negatives of this stringent computational pipeline, two evaluations were performed on the basis of the human YH genome and the associated poly(A)-positive RNA-Seq data [7], which were used previously to identify human editing sites [7], [21]. First, we applied the identical pipeline and inclusion criteria used in our study on this dataset to identify human editing sites [7]. Second, considering the total sequencing depth of this human study is much lower than that of our study in rhesus macaque [7], the inclusion criteria for RNA-editing sites were slightly modified by decreasing from five to two the minimum RNA-Seq reads required to support the variant form (while keeping all parameters used in sequence alignment and single-nucleotide variation calling).

### Characteristics of RNA-editing sites

The levels of RNA editing were estimated separately for high-throughput, medium-throughput and low-scale data on the basis of read numbers [6], signal intensity contrast [44] and peak height ratio [45] between wild-type and edited forms, respectively. The sequence motif was built by Two Sample Logo [46], with the level of preference/depletion shown in height proportional to scale (**Figure 4A**).

We evaluated the dependence of editing levels on sequence motif. The RNA-editing sites were divided into four categories according to the nearby sequence preferences (**Figure 4A**), with a ‘matched’ motif referring to the consensus sequence of **YAS** [**Y** = **T**/**C**, **S** = **C**/**G**], a ‘5′ matched’ motif of **YAW** [**W** = **A**/**T**], a ‘3′ matched’ motif of **RAS** [**R** = **A**/**G**], and a ‘not matched’ motif of **RAW**. We further performed a quantitative study to estimate how much site-to-site variances could be explained by the nearby sequence motif. We fitted the relationship between editing level and the local sequence context by controlling for cross-tissue and intra-population variations, using a Triplet model as previously described [29]:
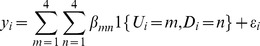
Where, 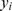 indicates the editing level of *i* th editing site; A, T, C and G were denoted by 1, 2, 3 and 4; 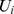 and 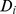 represented the 1 bp upstream or downstream nucleotide of the *i* th editing site; 1{A} was characteristic function (when A is satisfied, 1{A} = 1, otherwise, 1{A} = 0.); 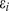 was the normally-distributed error term. The adjusted *R^2^* values obtained under the regression model was used to indicate the prediction power of the local sequence context on the editing levels [29].

### RNA-editing profile across individuals and tissue types

Public high-throughput datasets of multiple tissues from human [27], rhesus macaque [27], [47] and chimpanzee [27] were integrated and processed using a pipeline as previously reported [39]. Mass array-based genotyping data (Sequenom) from multiple tissues were also generated to profile the distribution of editing levels for sites in coding regions across animals and tissues, from which RNA-editing sites without reliable genotyping data were excluded. Hierarchical clusters were built using complete linkage hierarchical clustering by Cluster (v3.0), on the basis of editing levels across different tissues in different individuals, for all editing sites (**Figure 3B**), or for several subsets of these editing sites (**Figures 3C** **& S8**).

Besides the qualitative clustering data, we further measured the coefficients of variation (CV) of editing level across different animals, as well as across tissues. RNA-Seq data in brain samples from seven animals and seven tissues from the same animal were integrated and analyzed in standard pipelines for estimation of editing levels and CVs (**Table S4**). Only those editing sites covered with at least 30 RNA-Seq reads and at least 5 observations in each group were included. A CV score less than one indicates a smaller standard deviation than the mean, and thus a small intra-population variation for RNA editing levels.

Expression profiles of *ADARs* were estimated as previously reported [39] and tissue-specific correlation between RNA editing level and *ADAR* expression was analyzed. Only those editing sites covered with at least ten RNA-Seq reads in each of the seven tissues were included. A cutoff for Spearman's rank correlation coefficient at ≥0.5 was used to indicate a positive correlation between the tissue-biased profile of the RNA editome and *ADARs* expression profile, and sites correlated with both *ADARs* were considered to be associated with the one showing higher correlation coefficient. To further provide a quantitative estimate, we performed linear regression analysis to illustrate the association of *ADARs* expression profile with the editing levels:
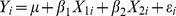
Where 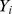 indicates the editing levels, *μ* the mean of editing level, 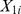 and 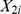 the expression levels of *ADAR1* and *ADAR2*, *β*_1_ and *β*_2_ the corresponding regression coefficients, and *ε_i_* the normally distributed error term. The *R^2^* was used as a quantitative indicator for the proportion of the variance of editing level that could be explained by the *ADAR* expression profile. 10,000 *Monte Carlo* simulations were performed to estimate the distribution of *R^2^* values on permutation datasets neglecting tissue relationships for the tissue expression profile. Finally, according to the test of significance of *β*_1_ and *β*_2_, we further classified sites as significantly correlated with *ADAR1* and/or *ADAR*2 using a cutoff of single-tailed test *p-value*≤0.05 and coefficient >0 (**Figure 4E & F**). Multiple testing corrections were performed as previously described (coefficient >0 and FDR<0.1) [**48**].

### Comparative genomics analyses and the detection of selective constraints

To examine whether the editing sites we identified in rhesus macaque could also be detected in human and chimpanzee orthologous regions, we re-analyzed public available RNA-Seq data in human or chimpanzee to trace the orthologous regions of macaque editing sites. Cross-species comparisons of RNA-editing regulation was analyzed in five tissues (cerebellum, prefrontal cortex, testis, kidney and heart) in the genomic context of sequence multiple alignments among human, chimpanzee and rhesus macaque [49]. Only macaque editing sites covered by at least 10 reads in both human and chimpanzee were included, a cutoff of sequence coverage required for accurate examination of the editing status: for the remaining sites with lower coverage, the assignment of the editing status is compromised by confounding factors such as the lower detection power of an editing site. To further confirm the authenticity of “editing signals” in other species, the adjacent non-edited sites were used as the background to indicate the degrees of sequence errors (**Figures 5A** **& S10**).

We then tested whether the cross-species similarities in RNA editome were maintained by purifying selection due to the functional implications of these regulations. The genomic sequences nearby all of these macaque editing sites were compiled and the percentage of sequence divergence between human and macaque was calculated for each 6-nucleotide window. To investigate whether higher expression may be linked with higher conservation, we performed the analysis again using three subsets of these editing sites, divided according to their abundance of expression tags, estimated on the basis of the sum of normalized abundance of expression tags in seven tissues and (**Figure S11E, F & G**).

For editing sites in coding regions, the *dS* and *dN* scores for each window nearby the editing sites were estimated using DnaSP (v5). The average *dS* and *dN* scores between human and rhesus macaque were estimated using 2,929 coding sequences with high-quality pairwise alignment [50]. Due to limited number of editing sites in coding regions, 10,000 *Monte Carlo* simulations with random sampling of coding regions across the macaque genome were performed to assess whether the detected divergence rate drop was a consequence of sampling bias on limited observations. A series of Perl and R scripts (v2.13.1) were implemented to perform these statistical analyses.

## Supporting Information

Figure S1 High-quality strand-specific RNA-Seq was performed for seven rhesus macaque tissues. (**A**) PHRED quality scores across all bases of reads. (**B**) Distribution of RNA-Seq reads across transcripts. Transcripts were binned into even intervals from 5′ to 3′, with the percentage of all short reads aligned to each interval shown as mean ± SD. (**C**) Efficiency of strand-specific sequencing strategy. PFC: prefrontal cortex. (**D**) Average read distribution in exonic, intronic and intergenic regions as measured by RPKM. (**E**) Mismatch frequency at each position of reads summarized as mean ± SD.
(TIF)

Figure S2 Statistics for whole-genome sequencing (WGS) and parallel exome capture and sequencing (Exome-Seq) in rhesus macaque. The reads coverage in WGS (upper panel) and Exome-Seq (lower panel) is summarized and shown in cumulative frequency plots.
(TIF)

Figure S3 Results of Sanger sequencing validation for all 29 candidate macaque editing sites in coding regions. For each candidate editing site (indicated by genome coordinates and red arrows), raw chromatograms of sequences derived from seven cDNA and the matched DNA (gDNA) samples are shown (**S3-1** to **S3-27**).
(PDF)

Figure S4 Results of Sanger sequencing validation for 79 candidate macaque editing sites in non-coding regions. For each candidate editing site (indicated by genome coordinates and red underlines) in intronic regions (**S4-1**), untranslated regions (**S4-2**), or intergenic regions (**S4-3**), raw chromatograms of sequences derived from one cDNA and the matched DNA (gDNA) samples are shown. ^*^Sites used in regression analysis.
(PDF)

Figure S5 The levels of RNA editing could be accurately estimated using RNA-Seq data. For each editing site with adequate read coverage, the levels of editing were estimated using different RNA-Seq datasets of macaque brain. Pair-wise Pearson correlation coefficients are shown in (**A**). The basic information on these public datasets is summarized in (**B**).
(TIF)

Figure S6 Enrichment of the A-to-G editing sites by the multi-filter strategy. Initial: initial list of macaque editing sites identified by high-throughput sequencing. Relative representation of macaque editing types for sites that progressively passed the DNA filter, the RNA SNP filter, the strand-bias and RhesusBase filter, as described in **Materials and Methods**.
(TIF)

Figure S7 Quantitative analysis of intra-population and cross-tissue variations of editing levels. The distributions of coefficient of variance (CV) values within macaque animals (**A**), as well as across different tissues (**B**) are shown.
(TIF)

Figure S8 Hierarchical clustering of editing levels for subsets of editing sites. Editing levels were estimated on the basis of RNA-Seq data in this study (Testis, Lung, Kidney, Heart, Muscle, Prefrontal cortex) and other public RNA-Seq data [Brain (1–6), Cerebellum (1–2), Muscle (1–8), Heart (1–5), Kidney (1–3), Lung (1–3), Testis (1–3)], with missing data in dark cyan. Six subsets of editing sites, including sites in *Alu* (**A**), non-*Alu* repeat (**B**), non-repetitive (**C**), un-translated (**D**), intronic (**E**) and intergenic (**F**) regions were analyzed separately.
(TIF)

Figure S9 Distributions of *R^2^* values. Distributions of *R^2^* values in models assuming association of editing level of *Alu* (**A**) or non-*Alu* sites (**B**) with *ADARs* expression are shown.
(TIF)

Figure S10 Cross-species comparisons of different subsets of editing sites. The percentages of macaque editing sites that had corresponding editing sites in human and/or chimpanzee are denoted by red bars. Comparisons were done for the background using the adjacent non-edited sites to indicate the degrees of RNA-Seq sequence errors (**A**), as well as for the subsets of editing sites in different genomic context (**B**).
(TIF)

Figure S11 Signatures of purifying selection for editing sites in different subsets. The genomic sequences nearby the macaque editing sites were compiled according to the distances to the editing sites. For each 6-nucleotide window, the proportion of human-macaque (**A, B, D, E, F & G**) or human-chimpanzee (**C**) divergent sites is shown for different subsets of macaque editing sites. **A**: all macaque editing sites, **B**: conserved sites between human and macaque, **C**: non-edited sites in both human and chimpanzee, D: editing sites in *Alu* regions, **E–G**: editing sites with low, medium and high expression levels, respectively. The nearby regions are highlighted in red and the average divergence rate of the distal regions is indicated by blue dashed lines.
(TIF)

Figure S12 Distribution of levels of RNA editing in different genomic regions. For editing sites in untranslated, CDS, intronic and intergenic regions, the distribution of the levels of RNA editing is summarized in boxplot.
(TIF)

Table S1 Basic information of RNA-editing sites identified in this study.
(XLSX)

Table S2 Primers used in Sanger sequencing.
(PDF)

Table S3 Known human RNA-editing sites in coding regions. The percentage of “G” reads, together with the number of total reads (“A” and “G”) in each tissue sample is shown. *Yes: included in final list; DNA Depth: failed to pass inclusion criteria for read coverage; Filter: failed to pass the filter; ^#^Ce: cerebellum, Te: testis, Pr: prefrontal cortex, Ki: kidney, Lu: lung, Mu: muscle, He: heart.
(PDF)

Table S4 Statistics of public RNA-Seq data integrated in this study. ^$^PFC: prefrontal cortex; ^&^C: chimpanzee, H: human, R: rhesus macaque; ^#^M: million reads; ^*^Samples for cross-species comparison in **Figure 5A**; ^@^Samples for intra-species editing level comparison in **Figure 3B**; ^%^Samples for calculating intra-population CV values in **Figures 3D** **& S7**.
(PDF)

Table S5 *P-values* of one-tail Wilcoxon tests for four classes of motif. ^*^M: matched motif; 5′ M: 5′ matched; 3′ M: 3′ Matched; N: Not matched.
(PDF)

Table S6 Quantitative correspondence of the tissue-biased profile of the RNA editome to the tissue expression profile of *ADARs*.
(XLSX)
