# Supplementary figures and images for: RNA Editome in Rhesus Macaque Shaped by Purifying Selection
Source: PLoS Genet. 2014 Apr 10;10(4):e1004274. doi: 10.1371/journal.pgen.1004274 (PMC3983040; doi:10.1371/journal.pgen.1004274)

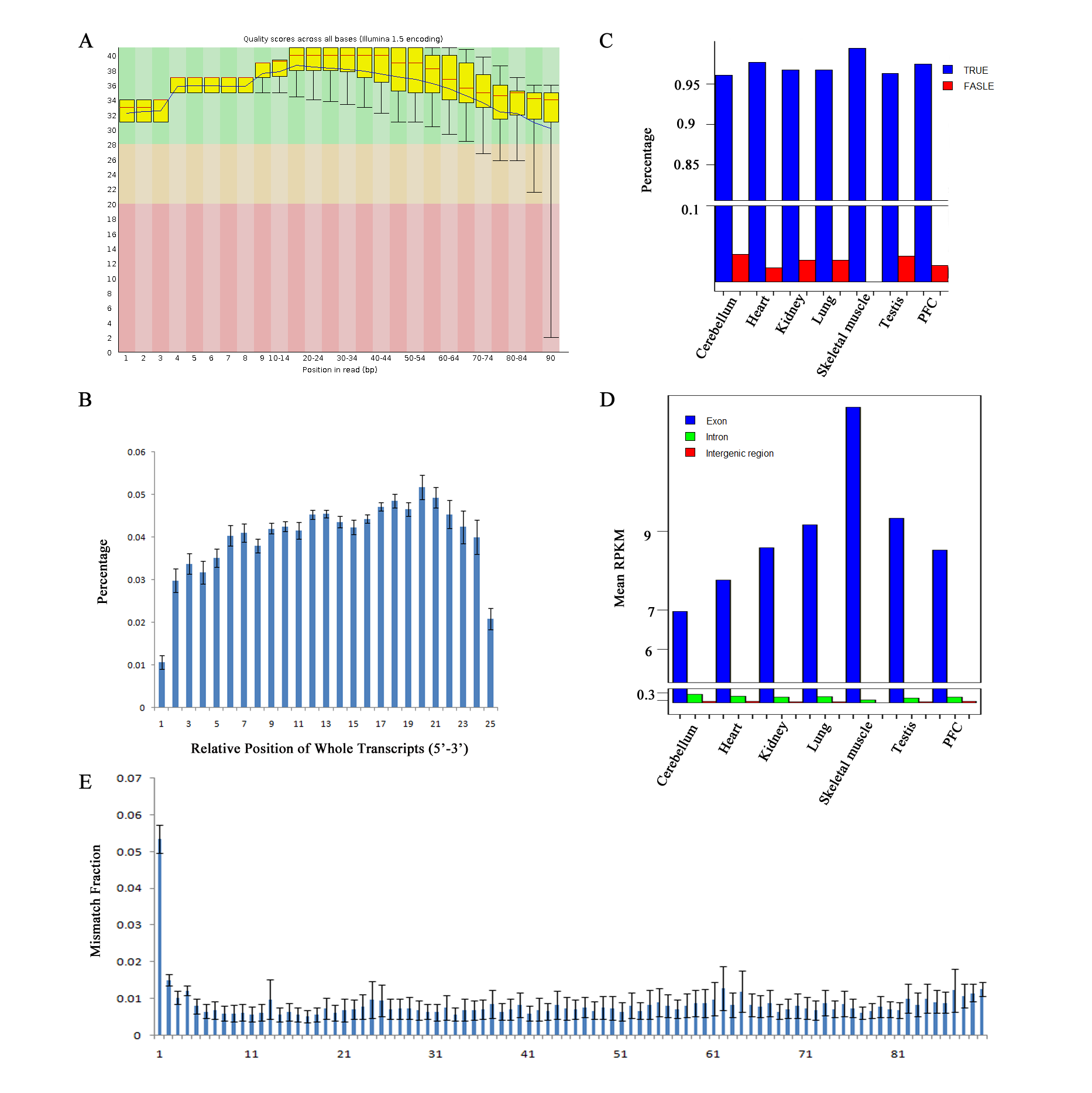

Supplement: Figure S1 — High-quality strand-specific RNA-Seq was performed for seven rhesus macaque tissues. (A) PHRED quality scores across all bases of reads. (B) Distribution of RNA-Seq reads across transcripts. Transcripts were binned into even intervals from 5′ to 3′, with the percentage of all short reads aligned to each interval shown as mean ± SD. (C) Efficiency of strand-specific sequencing strategy. PFC: prefrontal cortex. (D) Average read distribution in exonic, intronic and intergenic regions as measured by RPKM. (E) Mismatch frequency at each position of reads summarized as mean ± SD. (TIF) [file pgen.1004274.s001.tif]

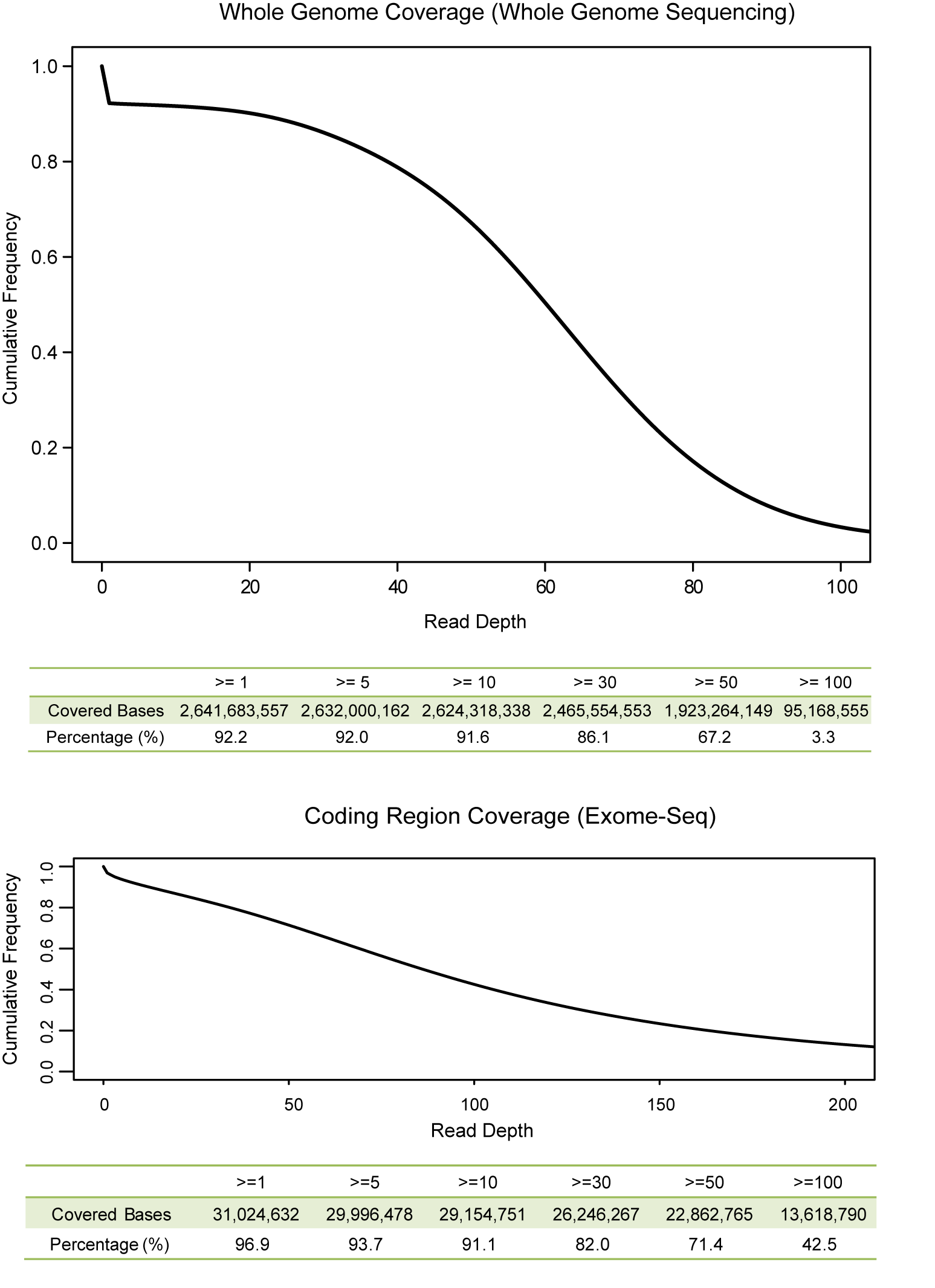

Supplement: Figure S2 — Statistics for whole-genome sequencing (WGS) and parallel exome capture and sequencing (Exome-Seq) in rhesus macaque. The reads coverage in WGS (upper panel) and Exome-Seq (lower panel) is summarized and shown in cumulative frequency plots. (TIF) [file pgen.1004274.s002.tif]

**Figure S3.**  
**S3-1**

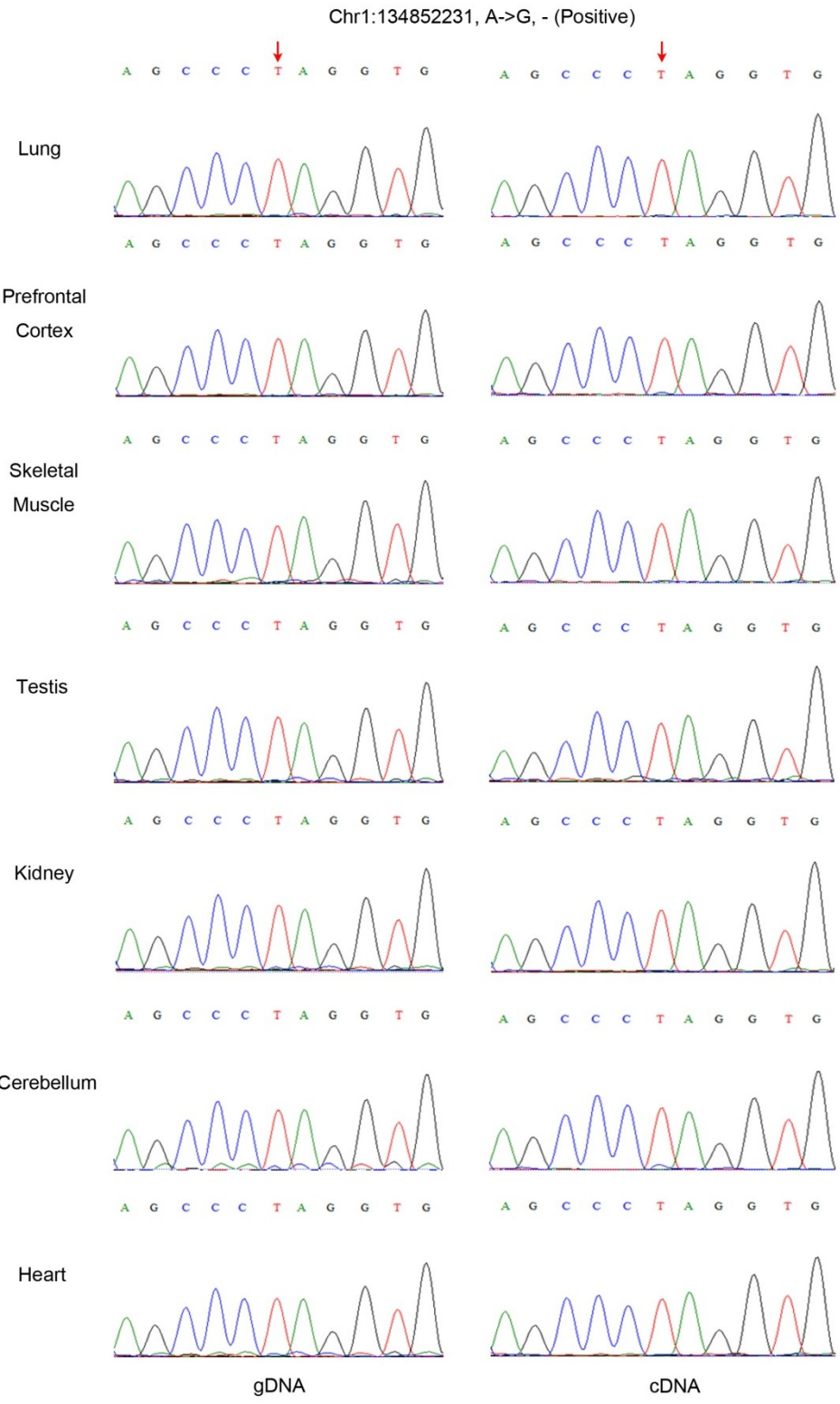

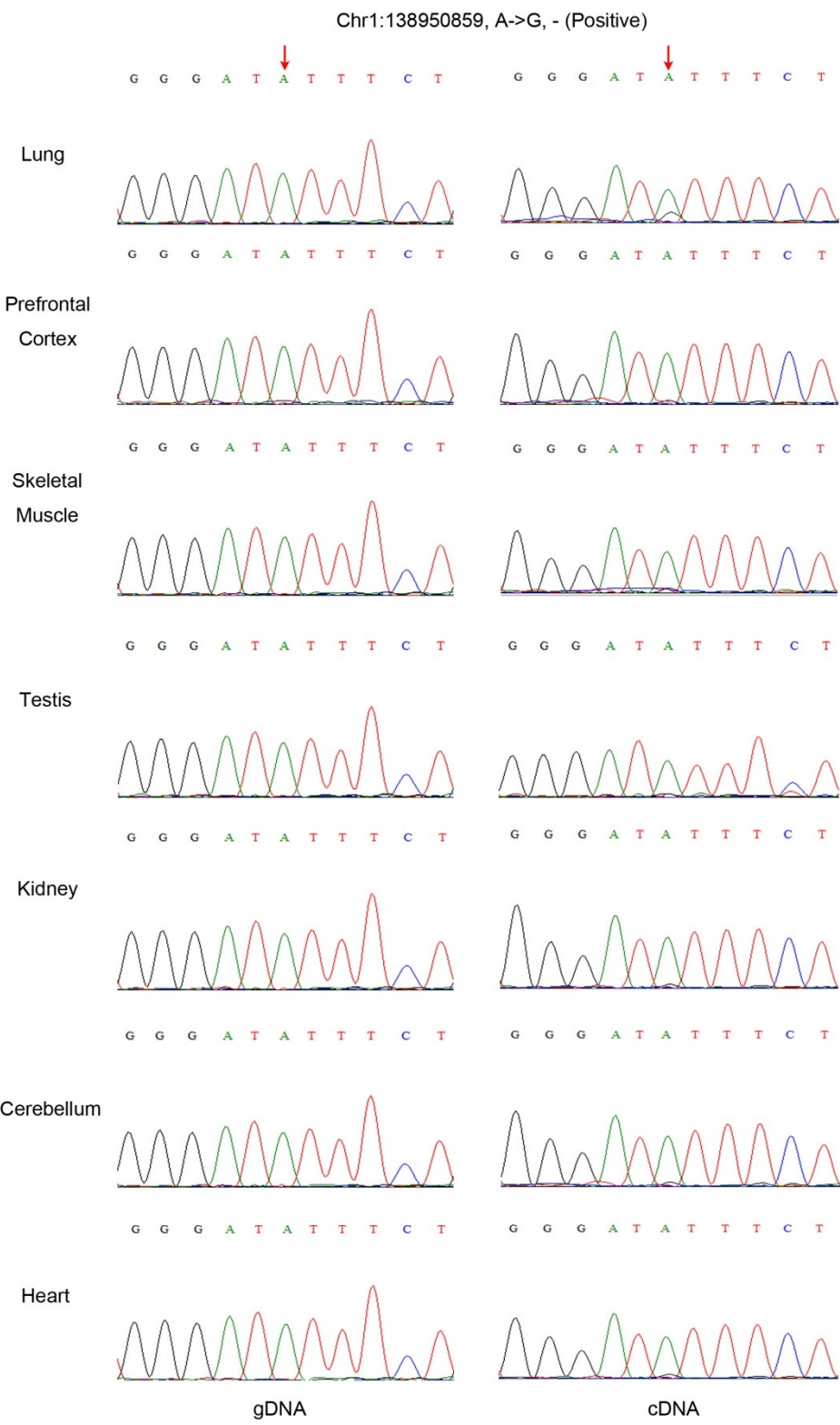

# S3-3

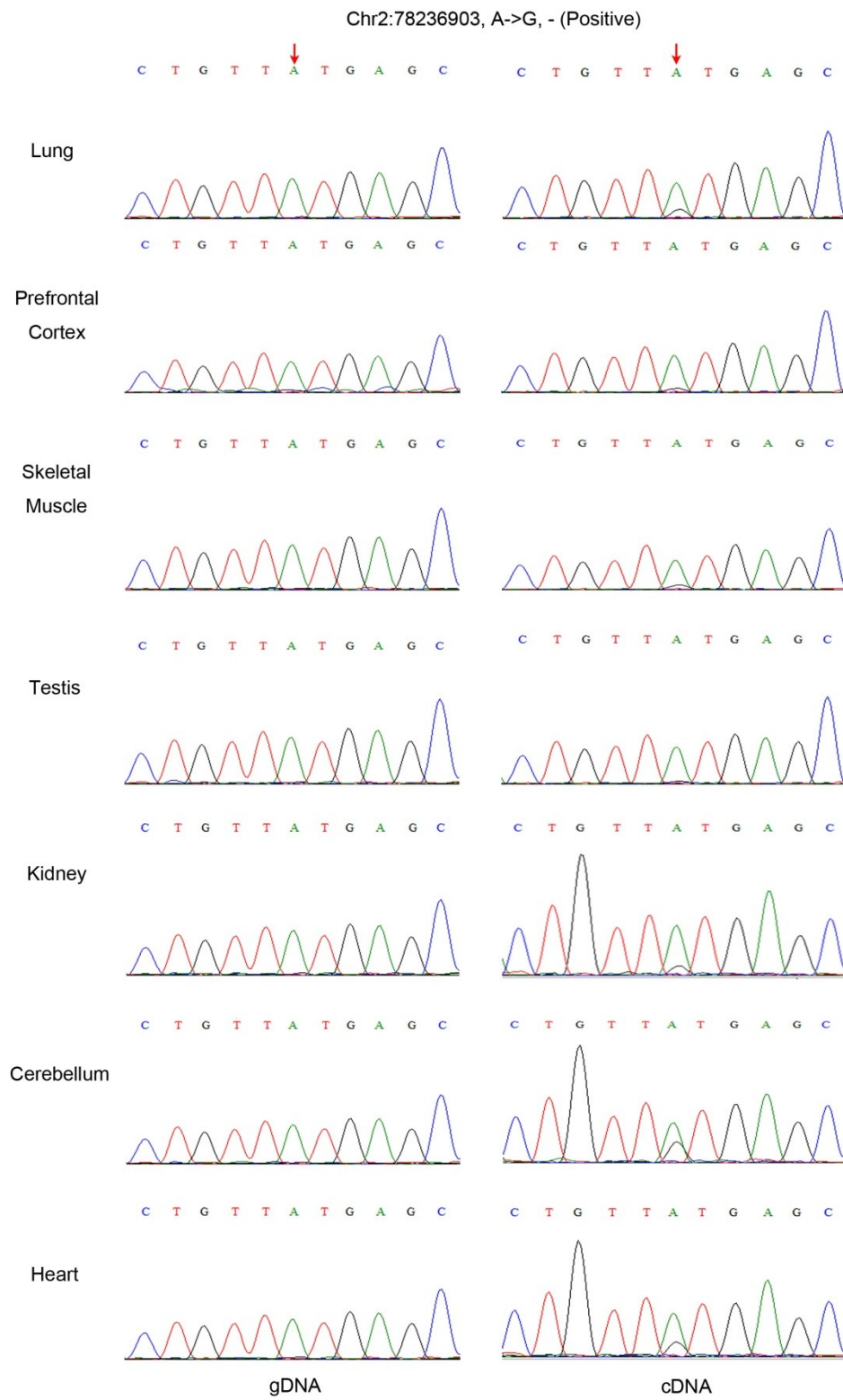

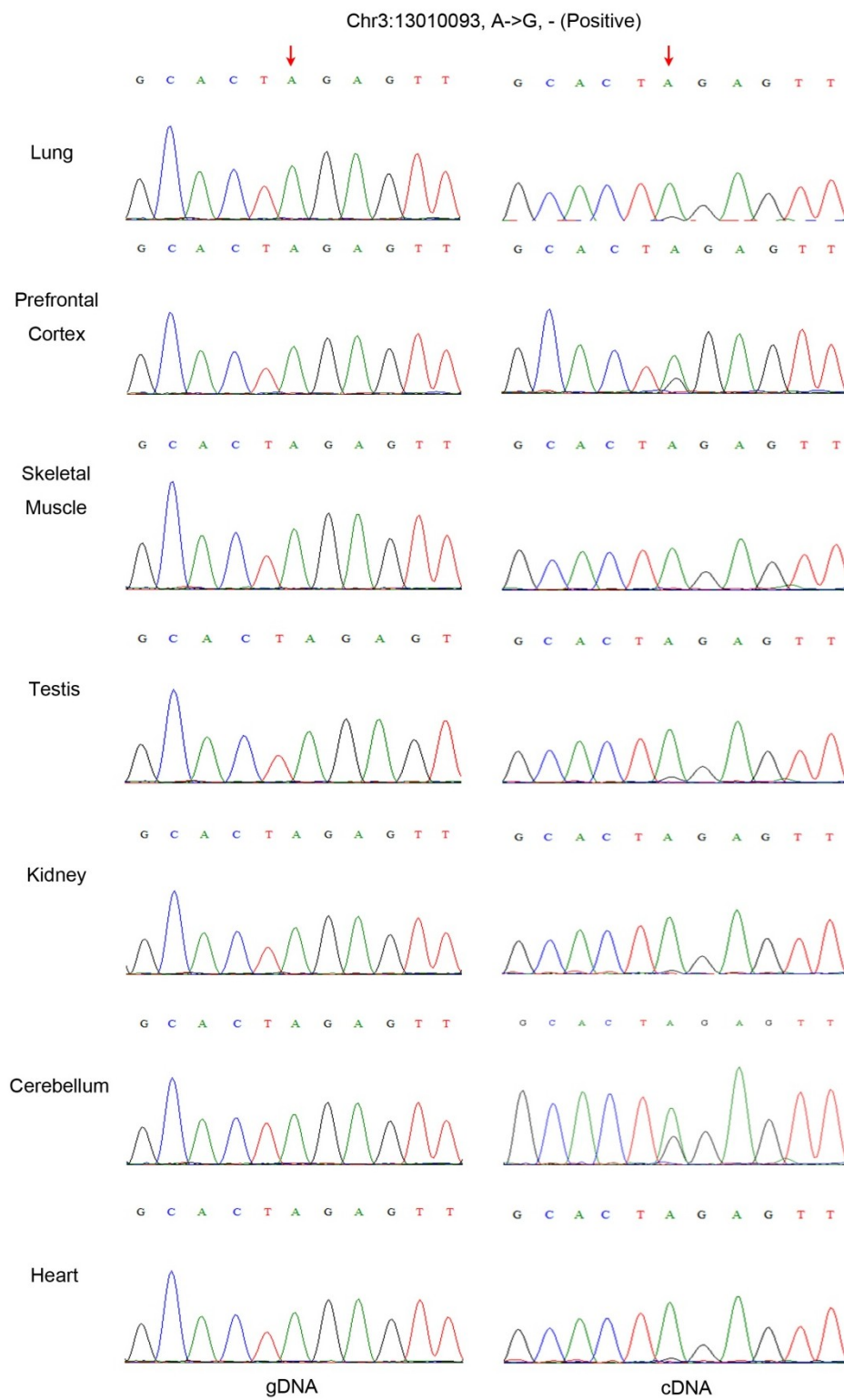

**S3-5**

Chr4:33833717, A->G, - (Positive)

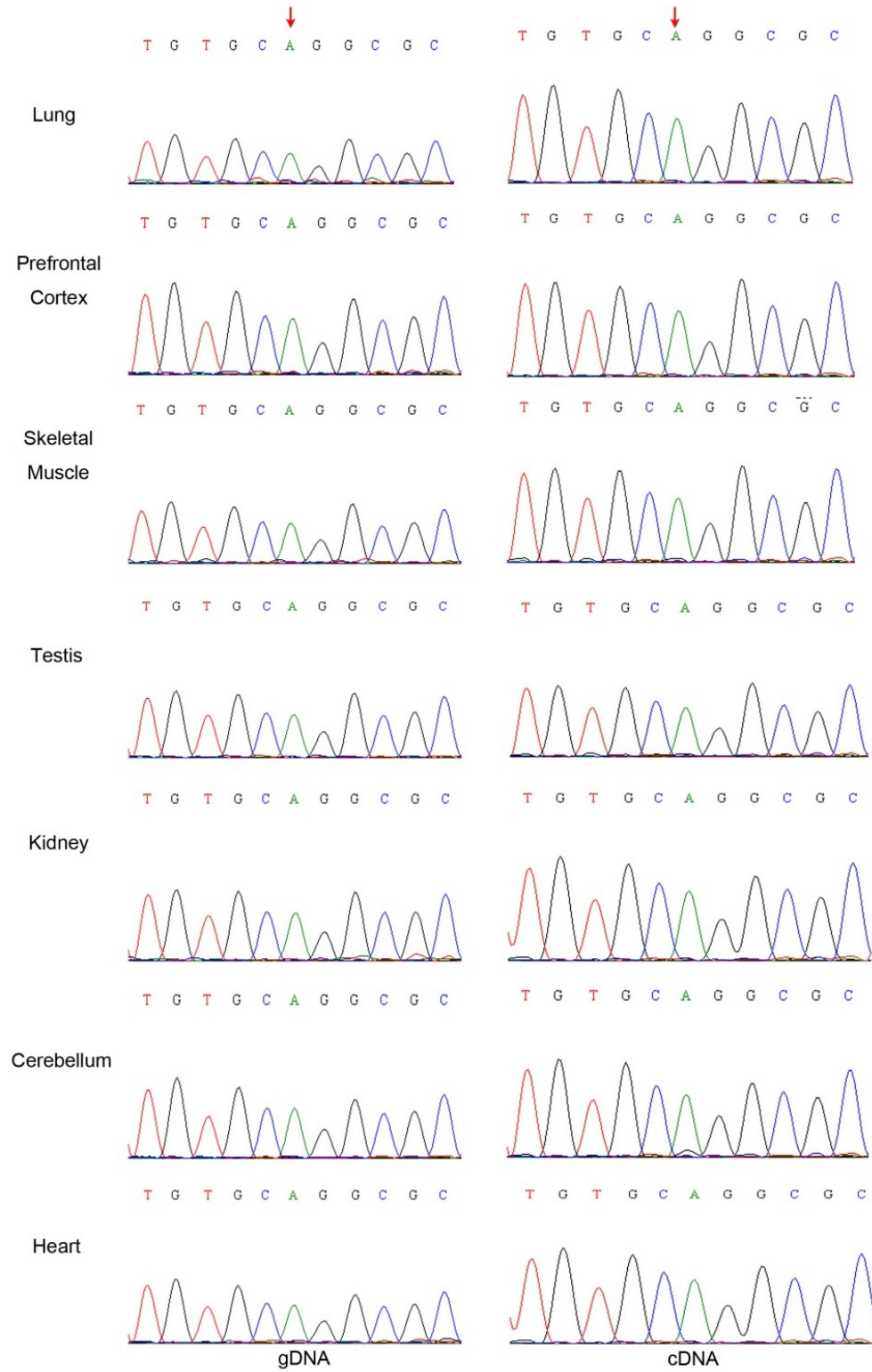

# S3-6

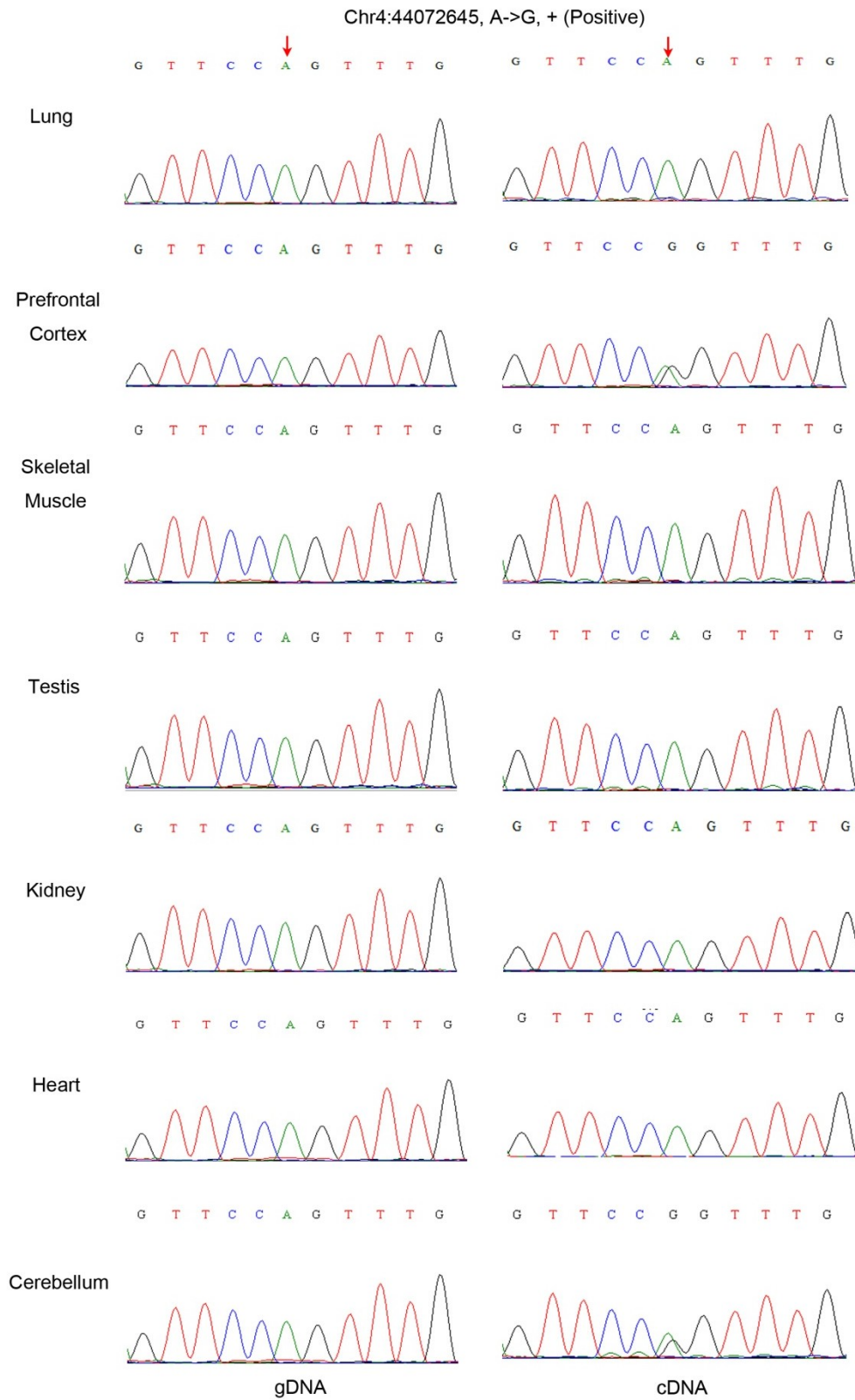

S3-7

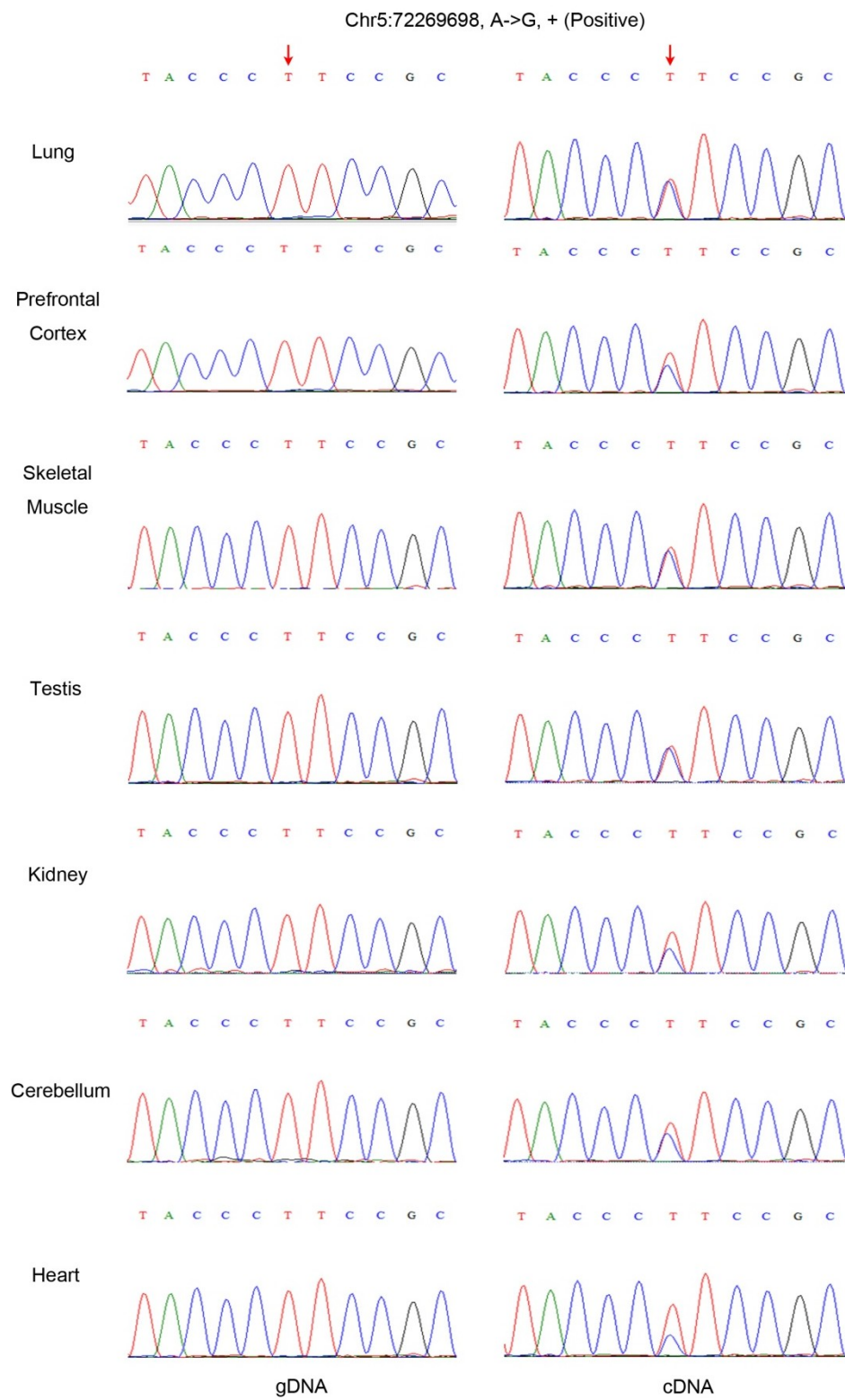

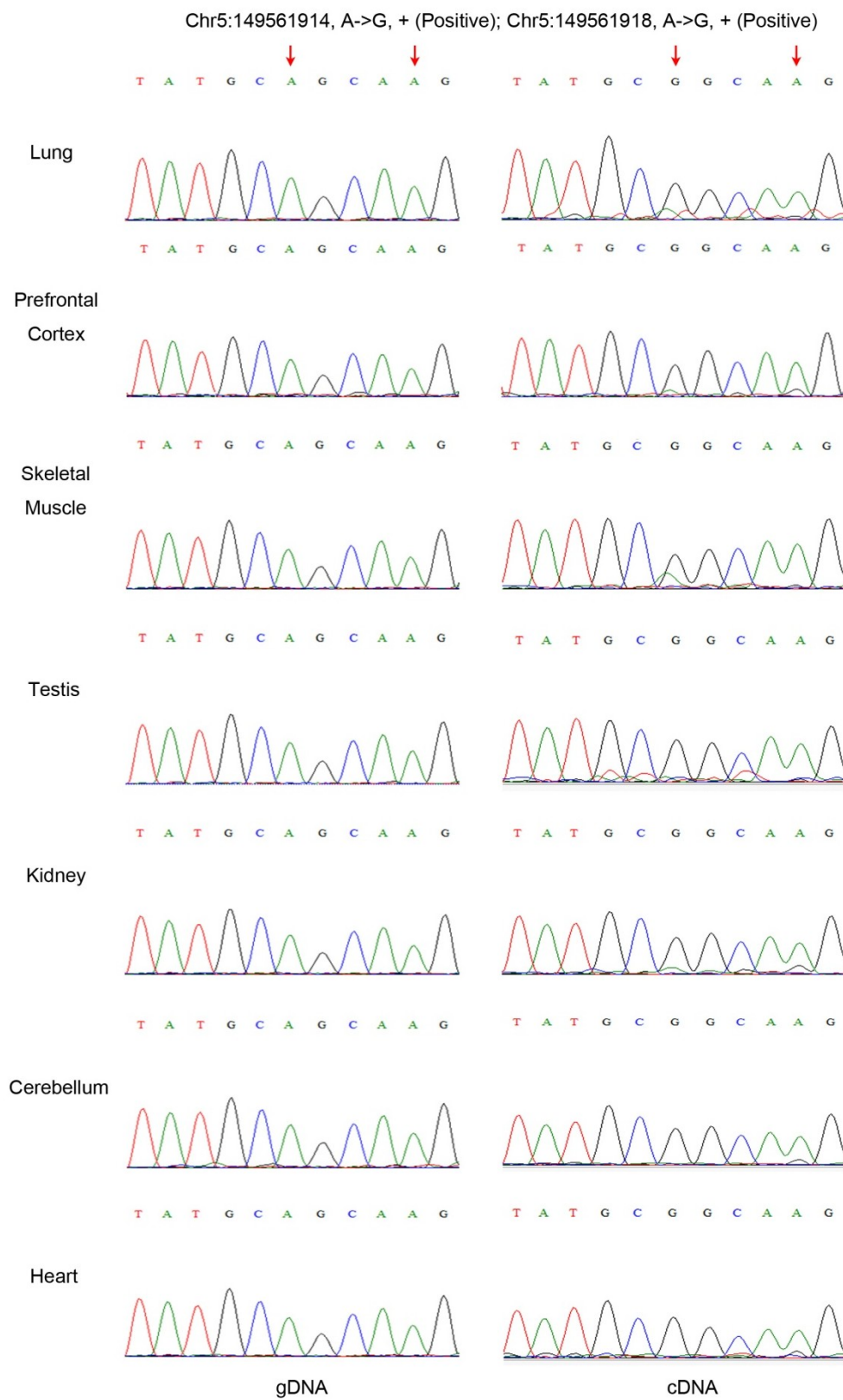

S3-9

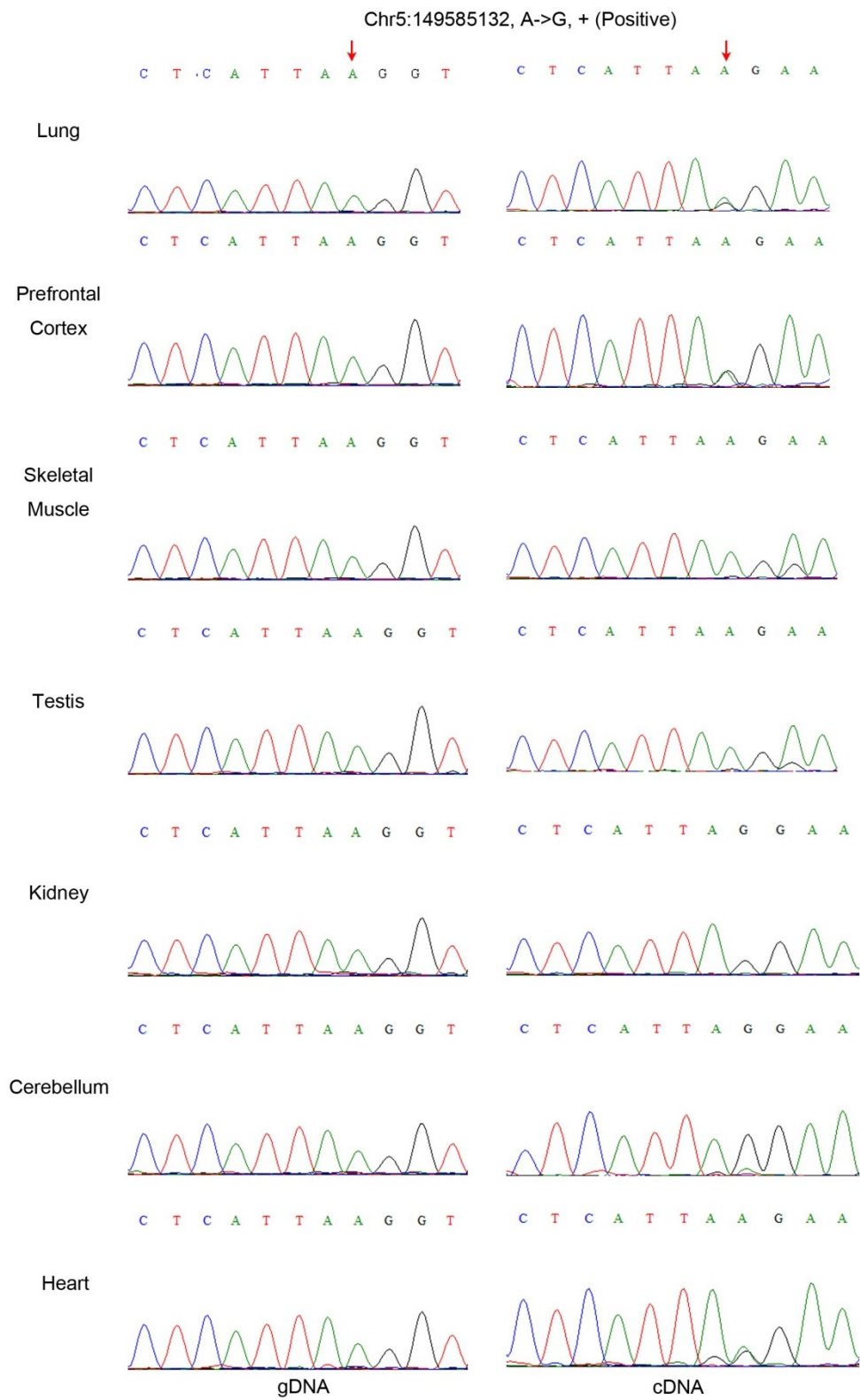

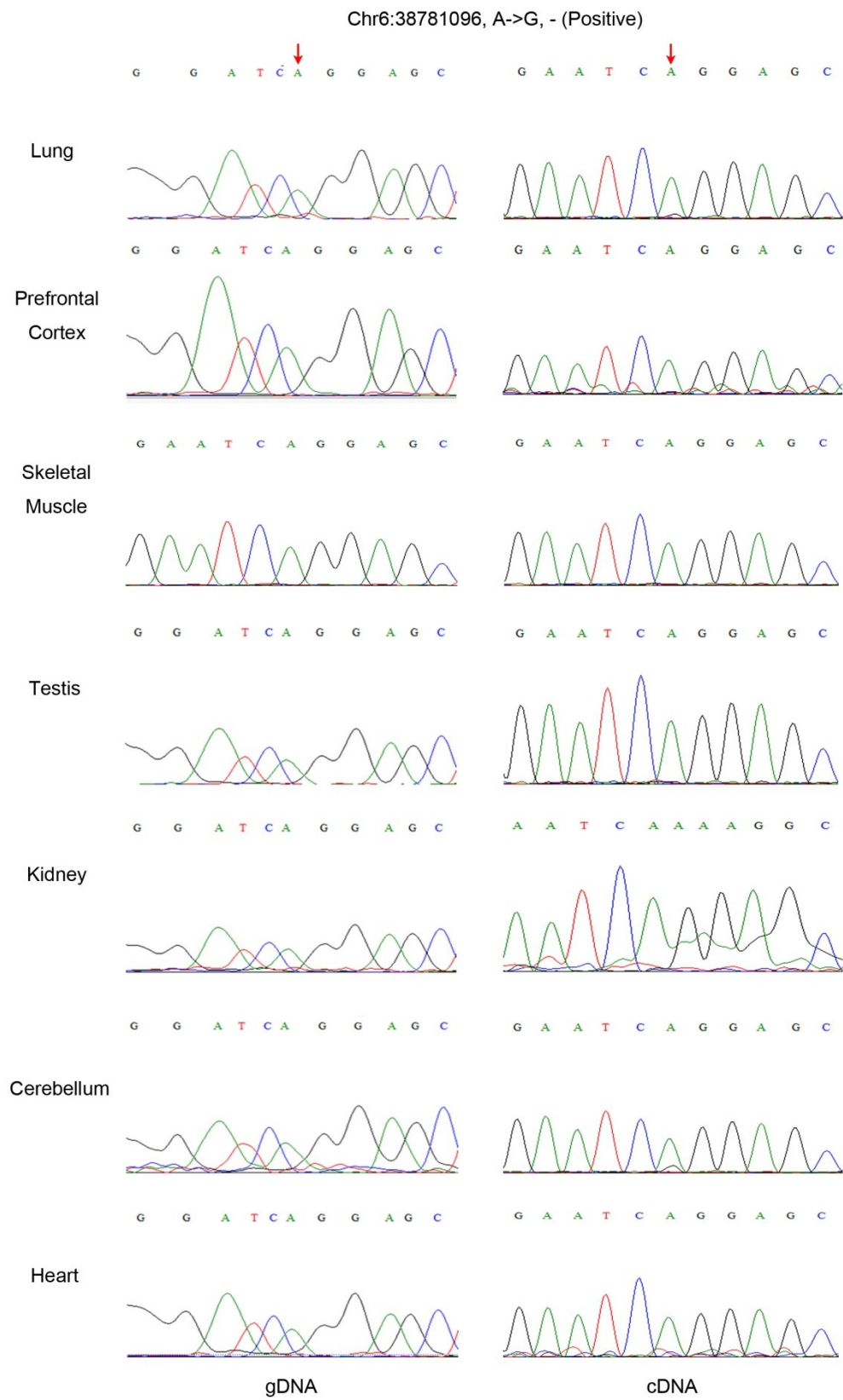

# S3-11

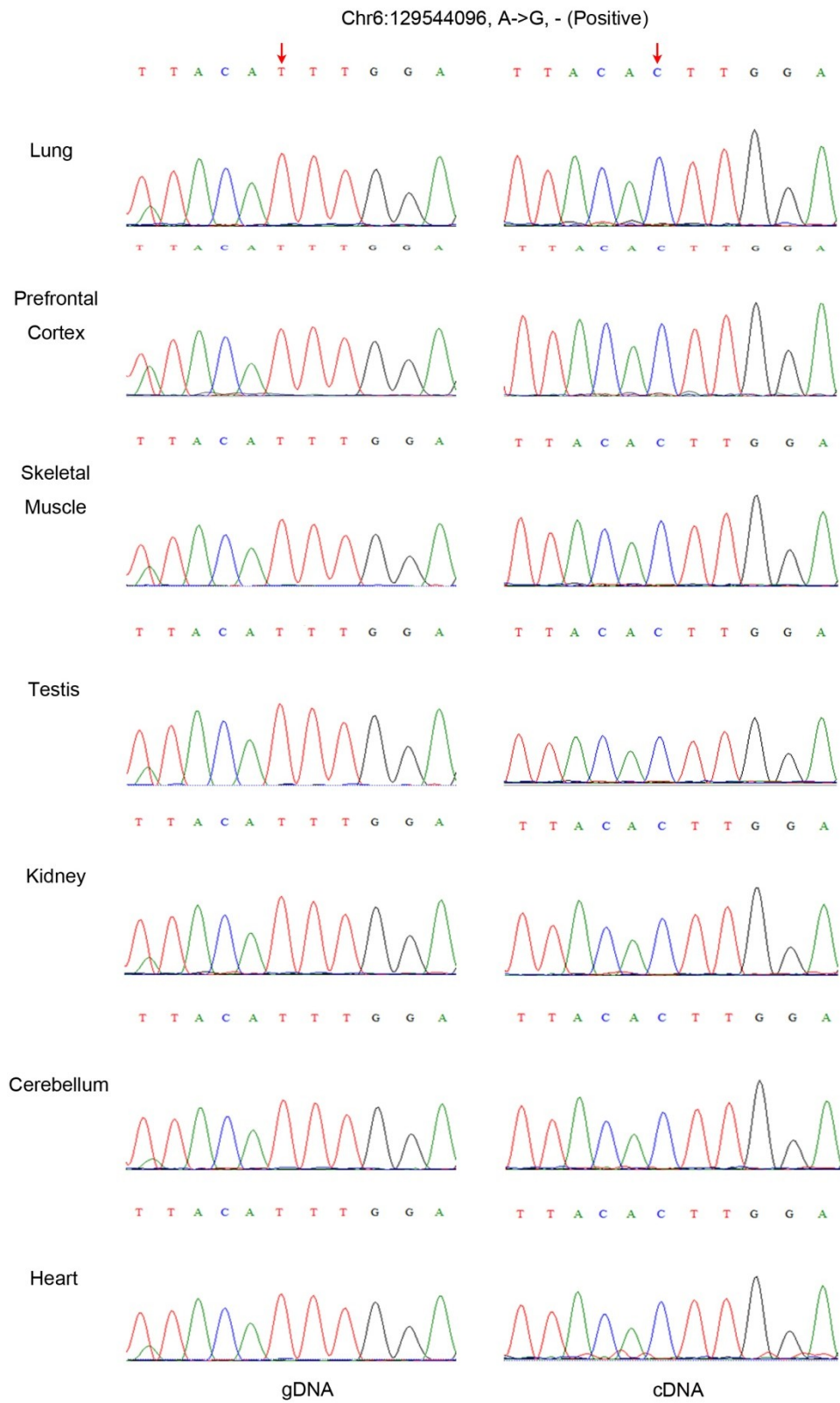

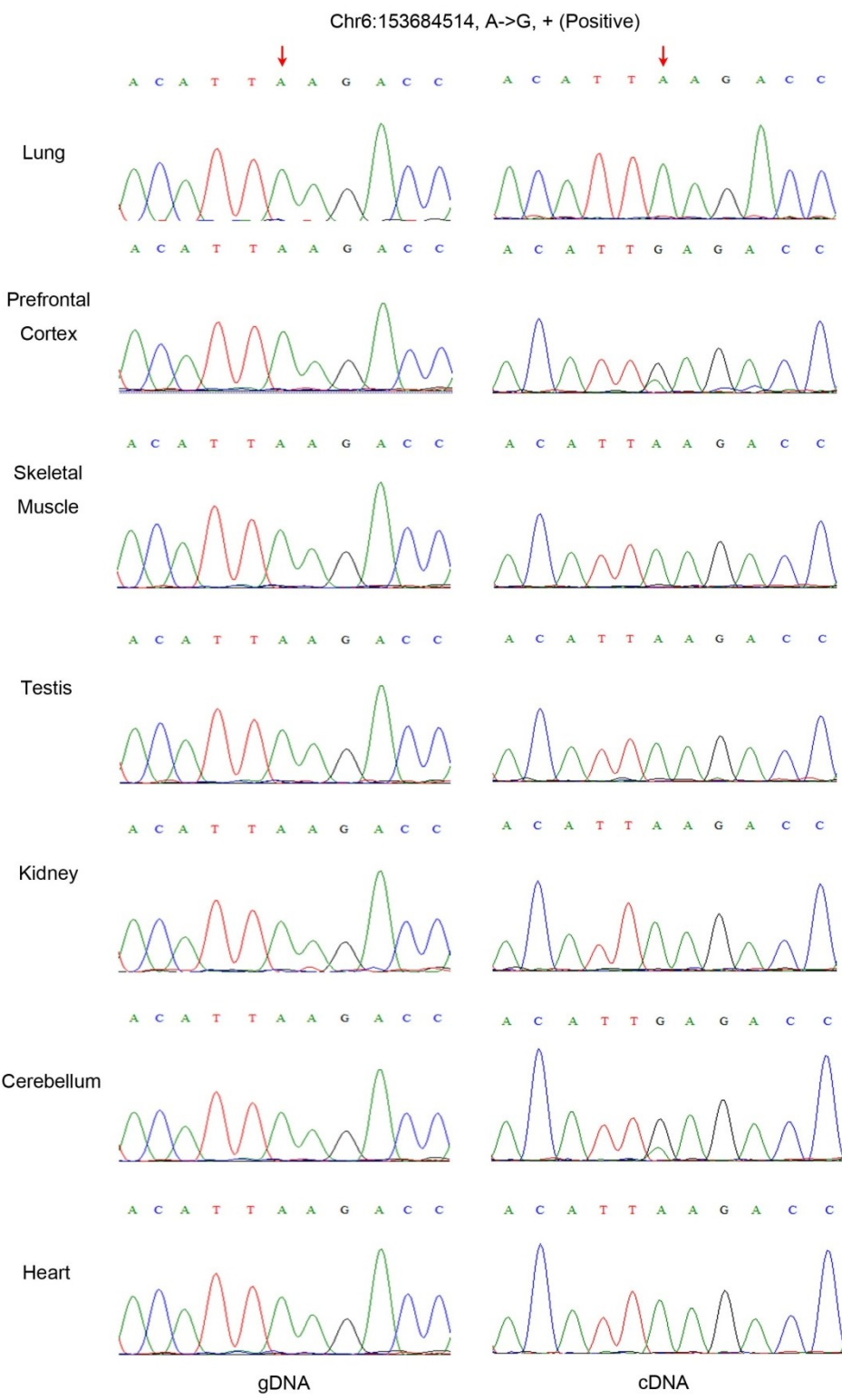

# S3-13

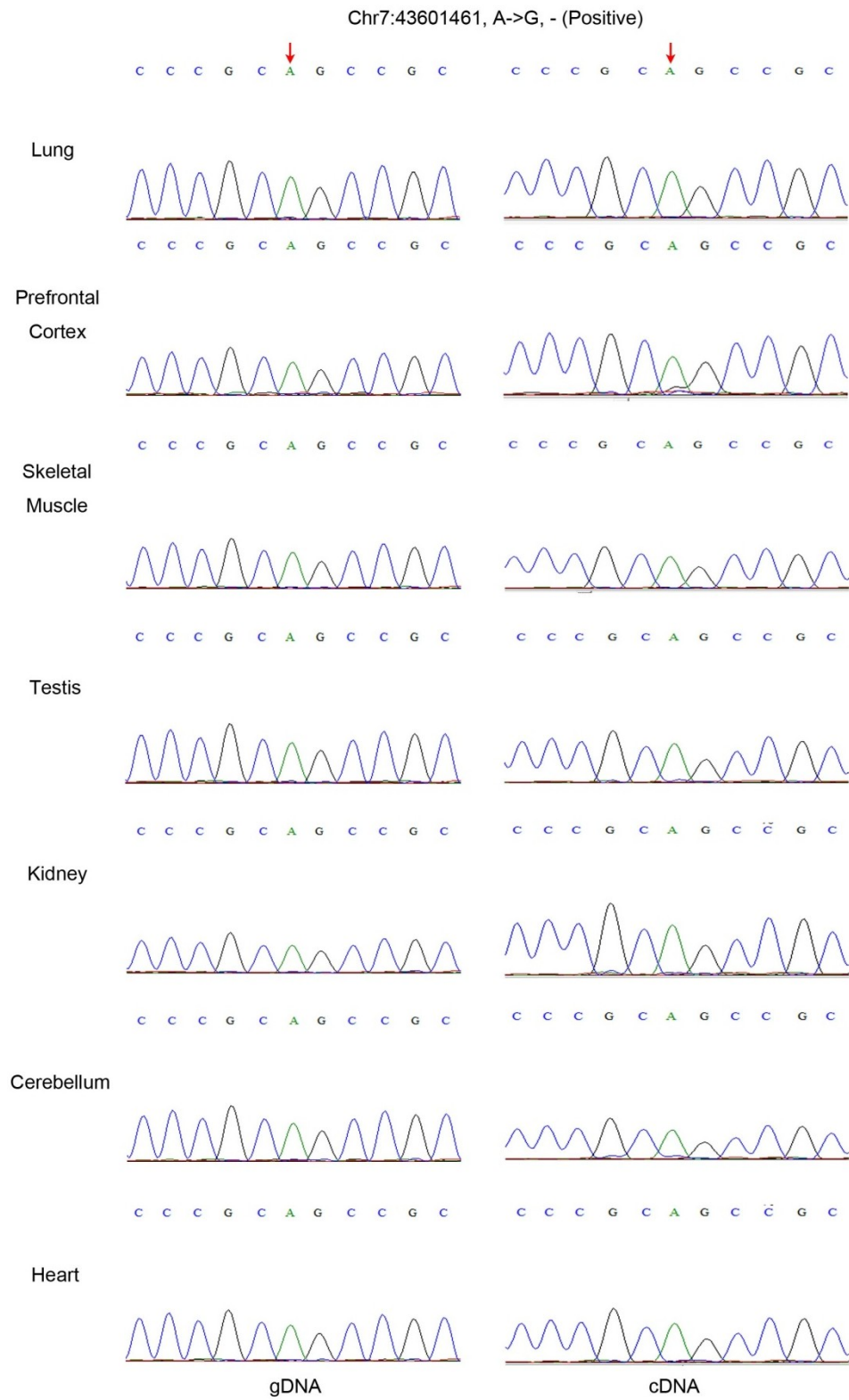

# S3-14

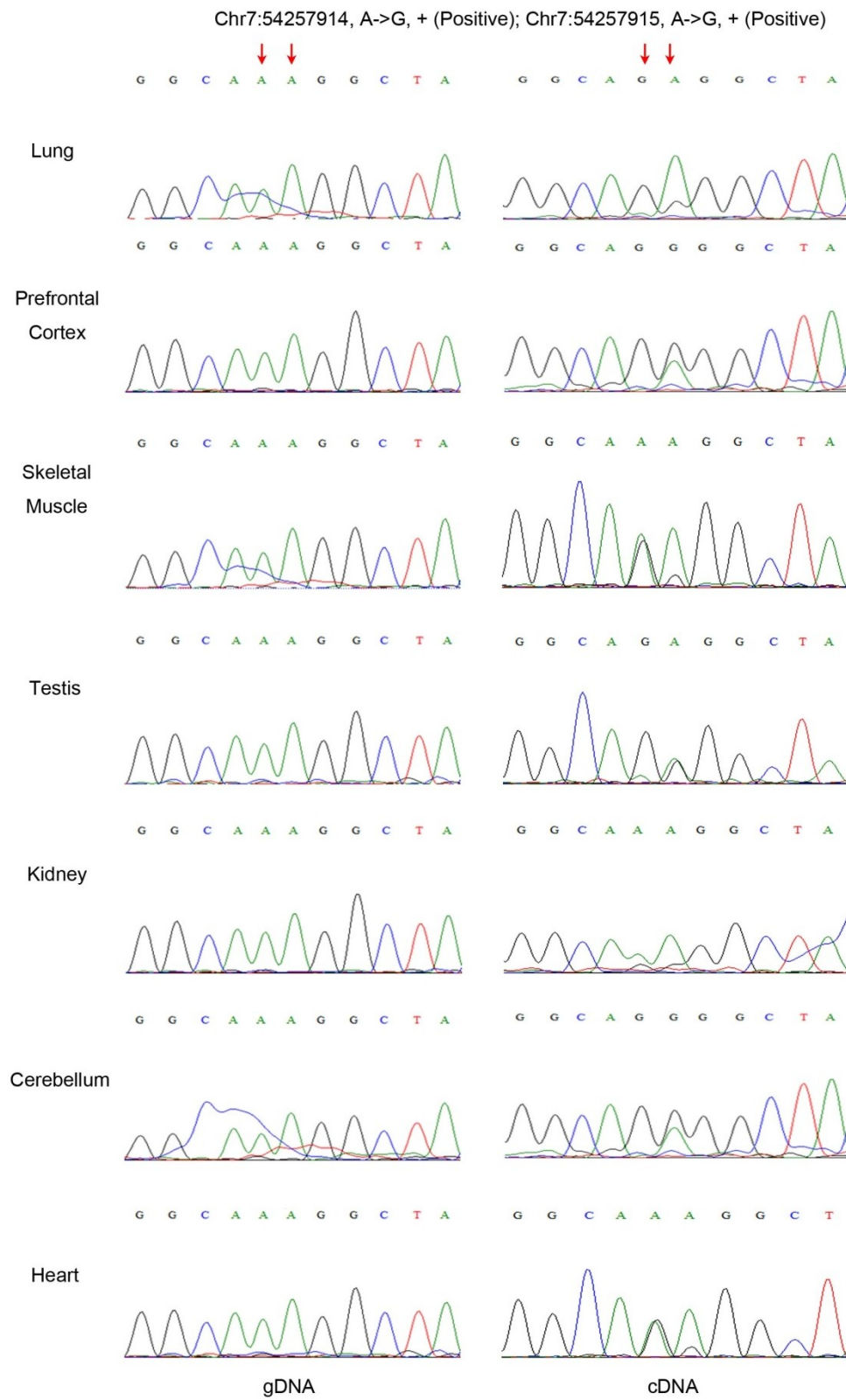

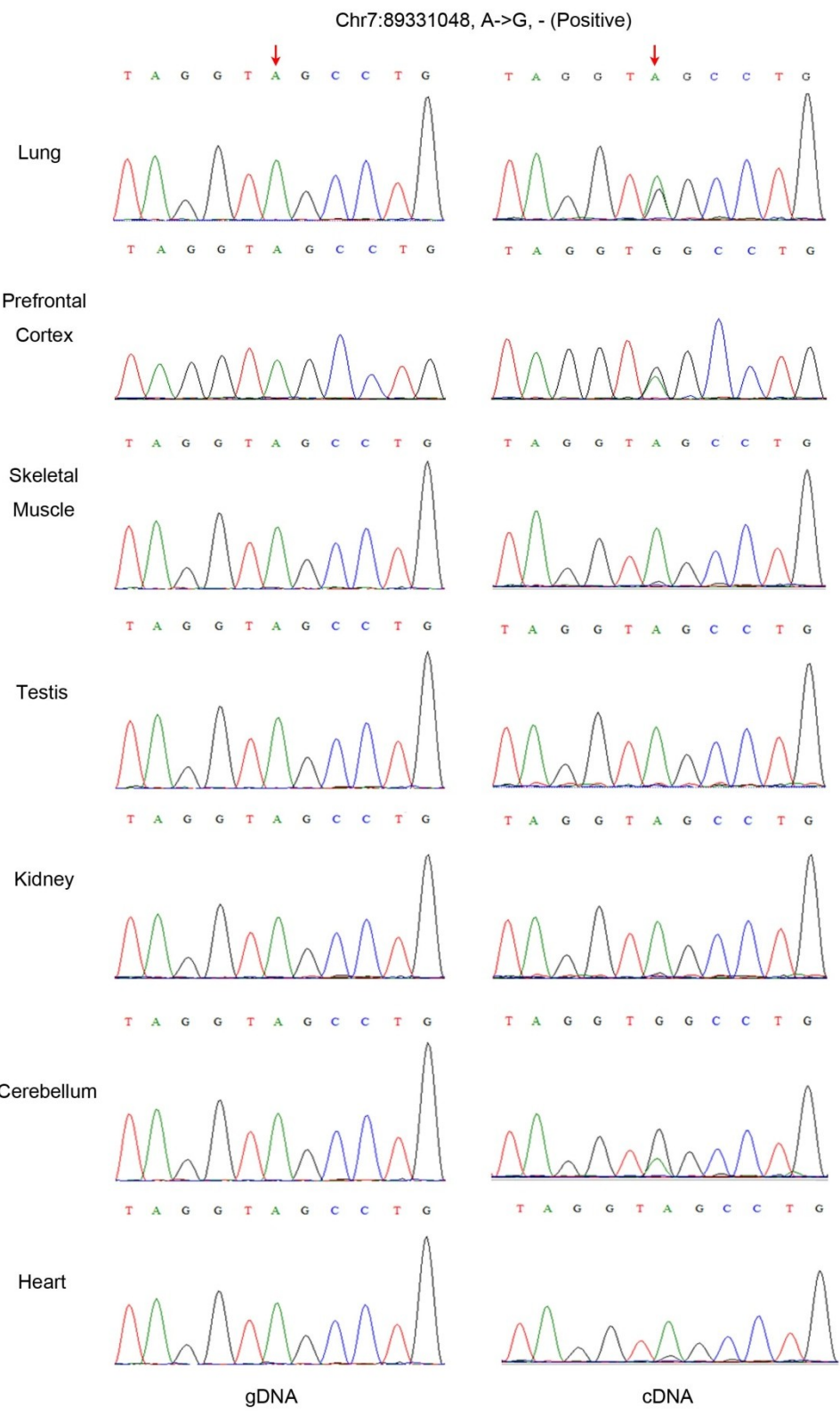

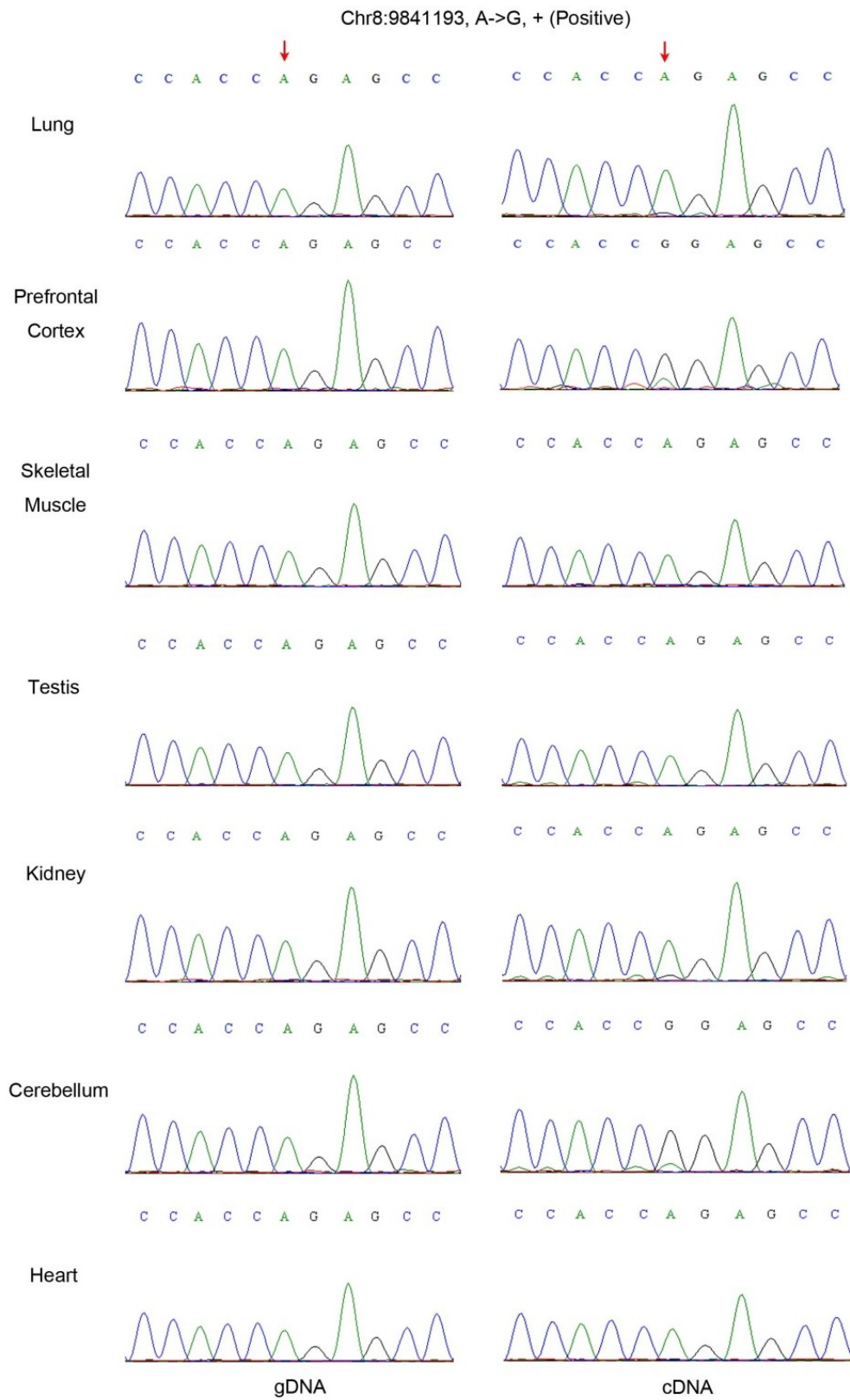

Chr10:26945910, A-&gt;G, + (Positive)

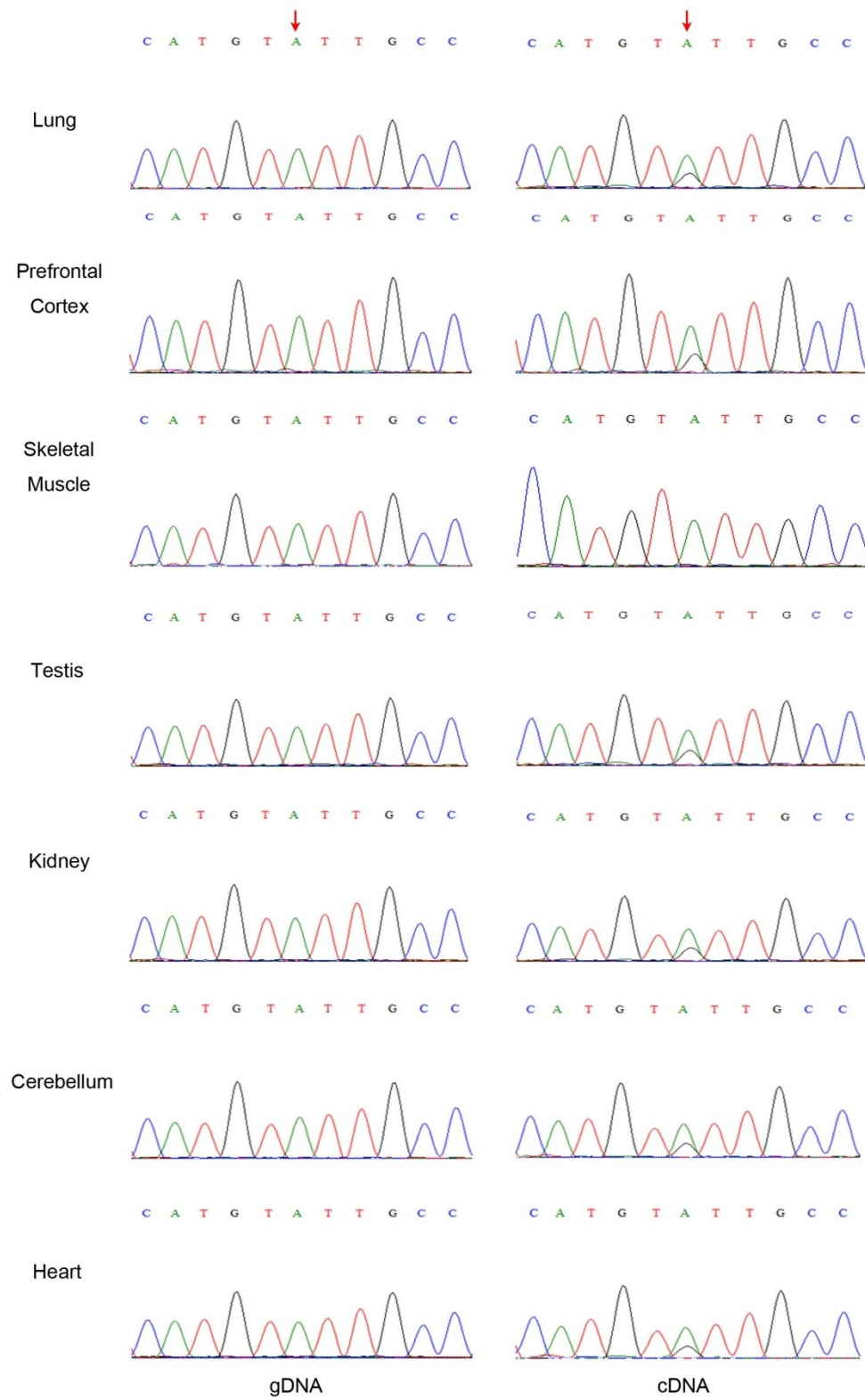

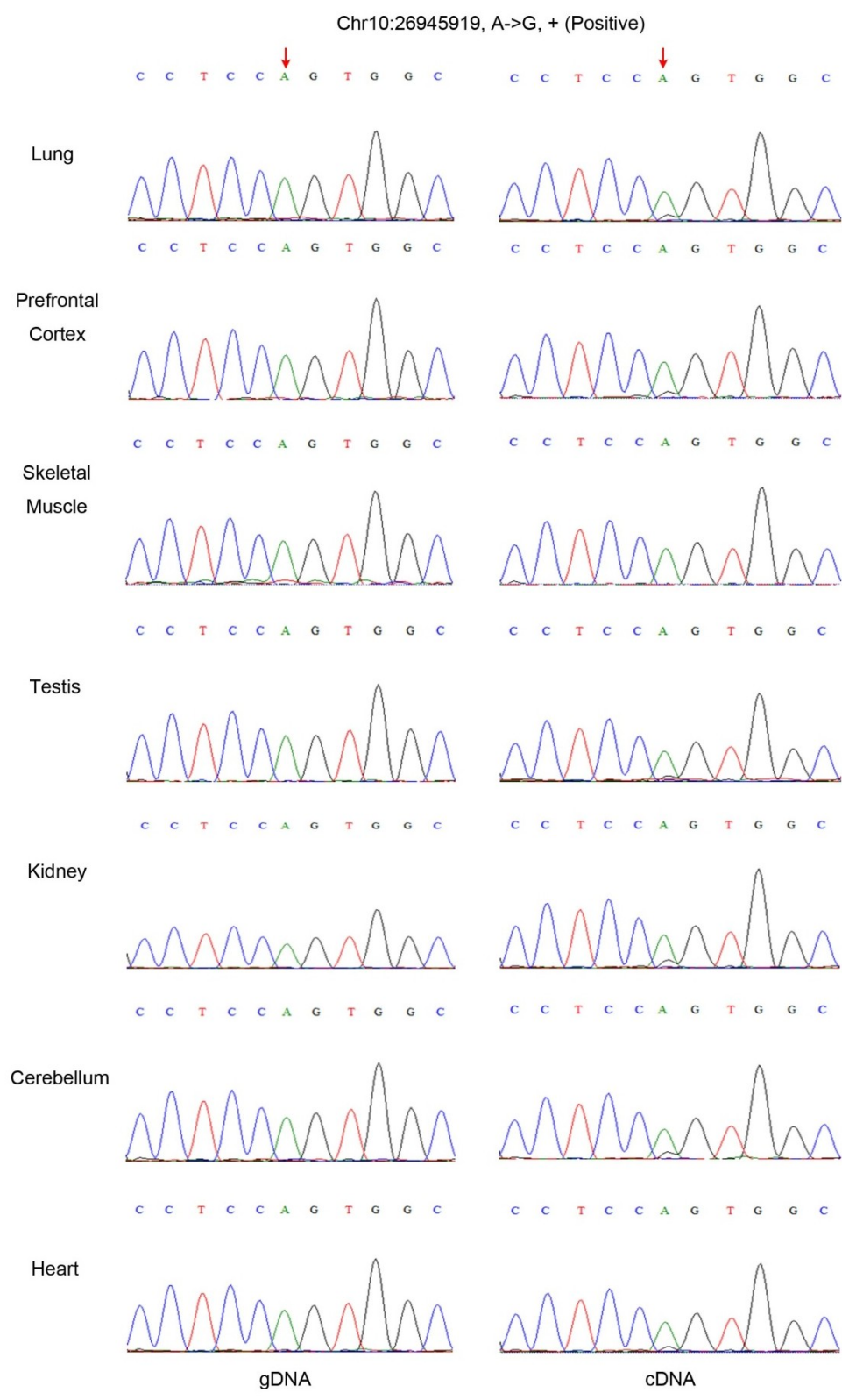

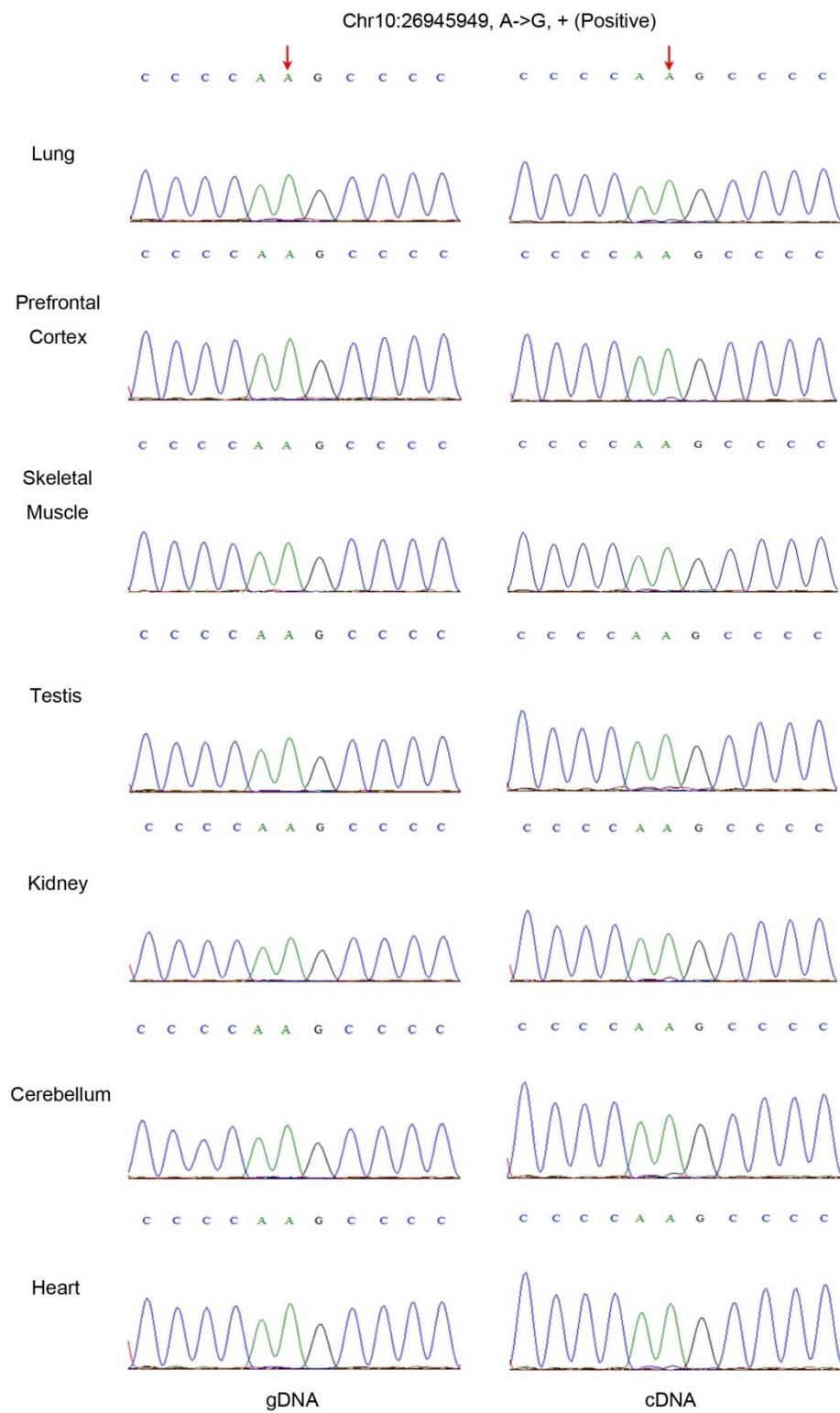

Chr11:5028364, A->G, + (Positive)

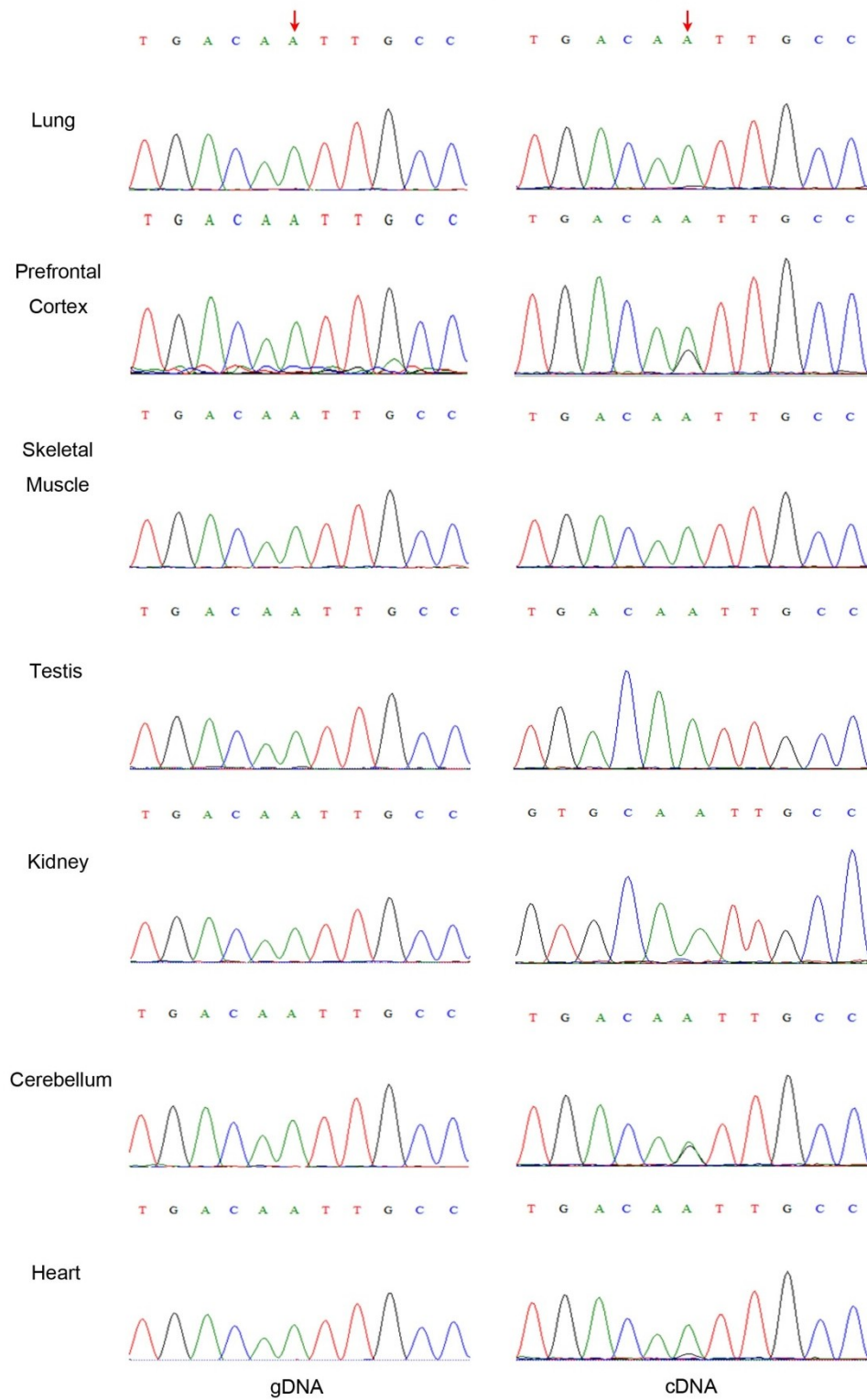

S3-21

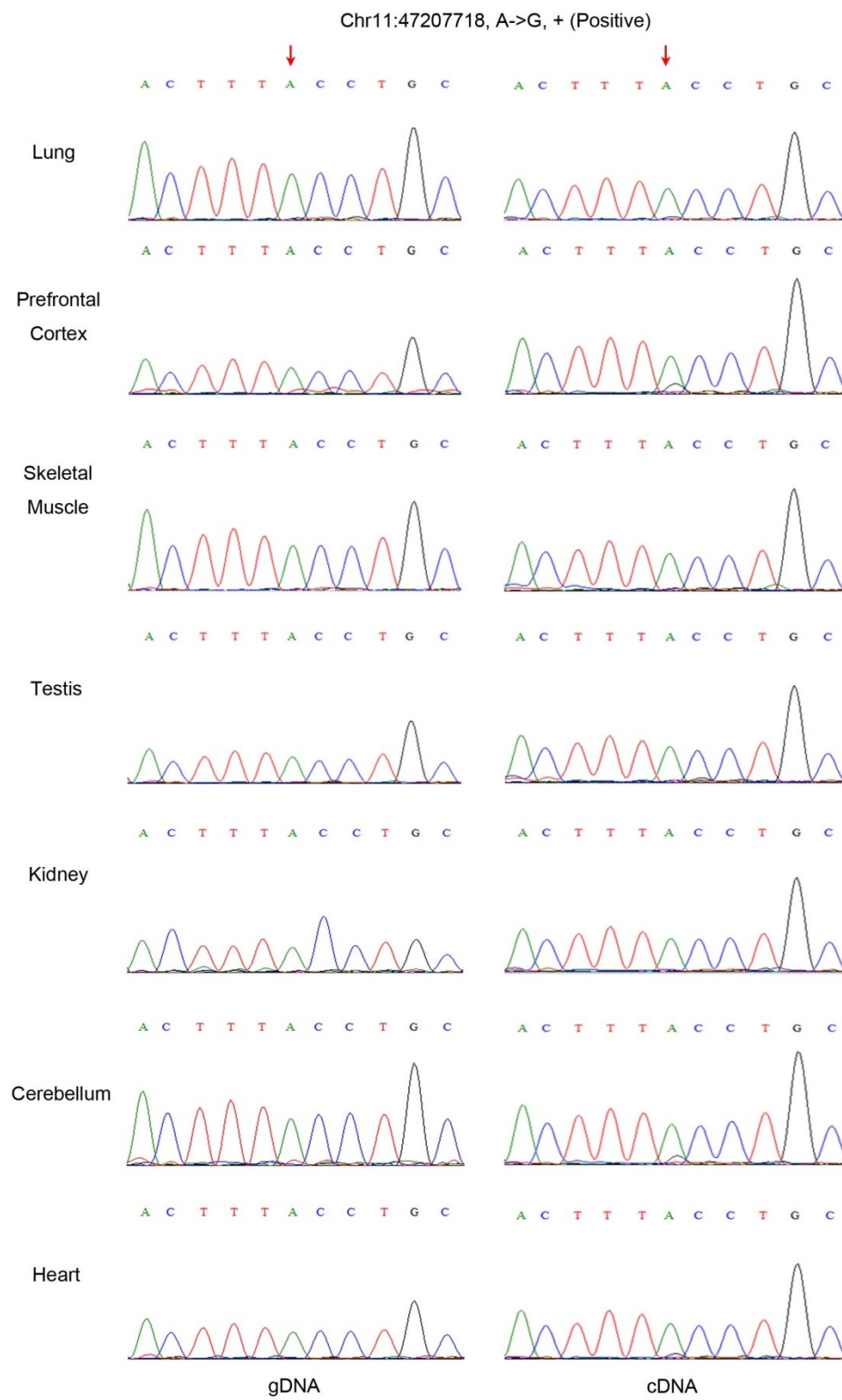

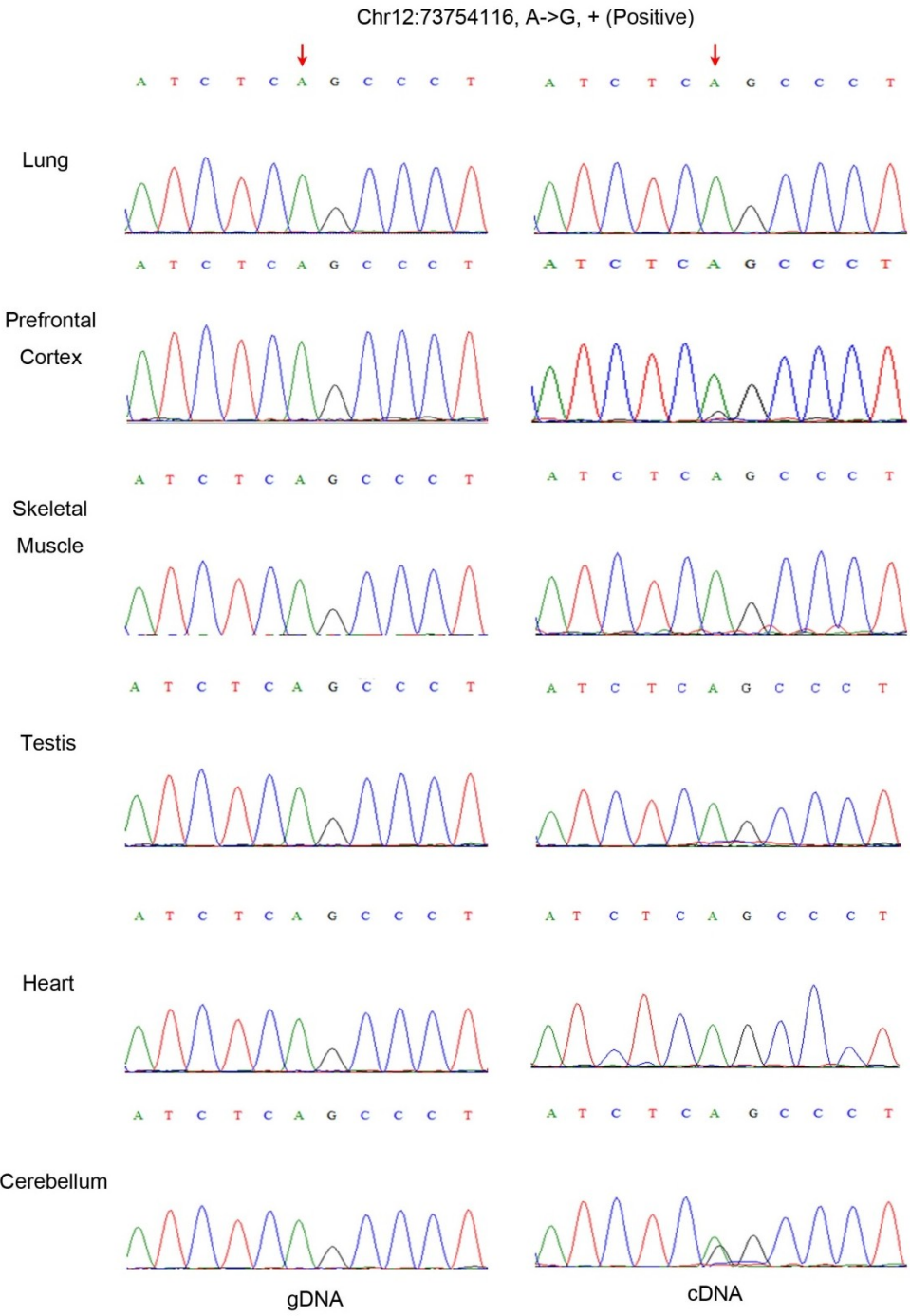

Chr17:24407704, A->G, + (Positive)

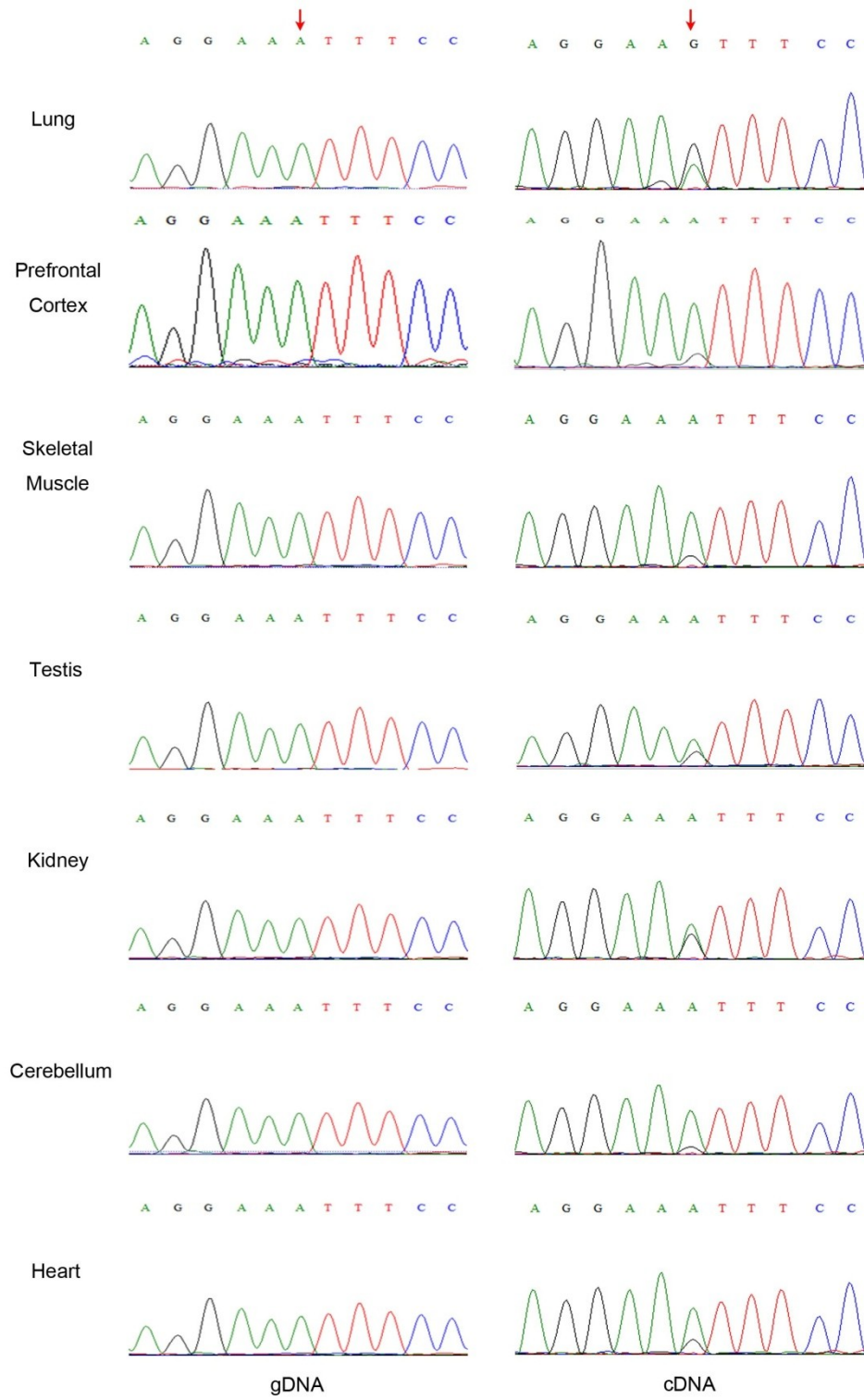

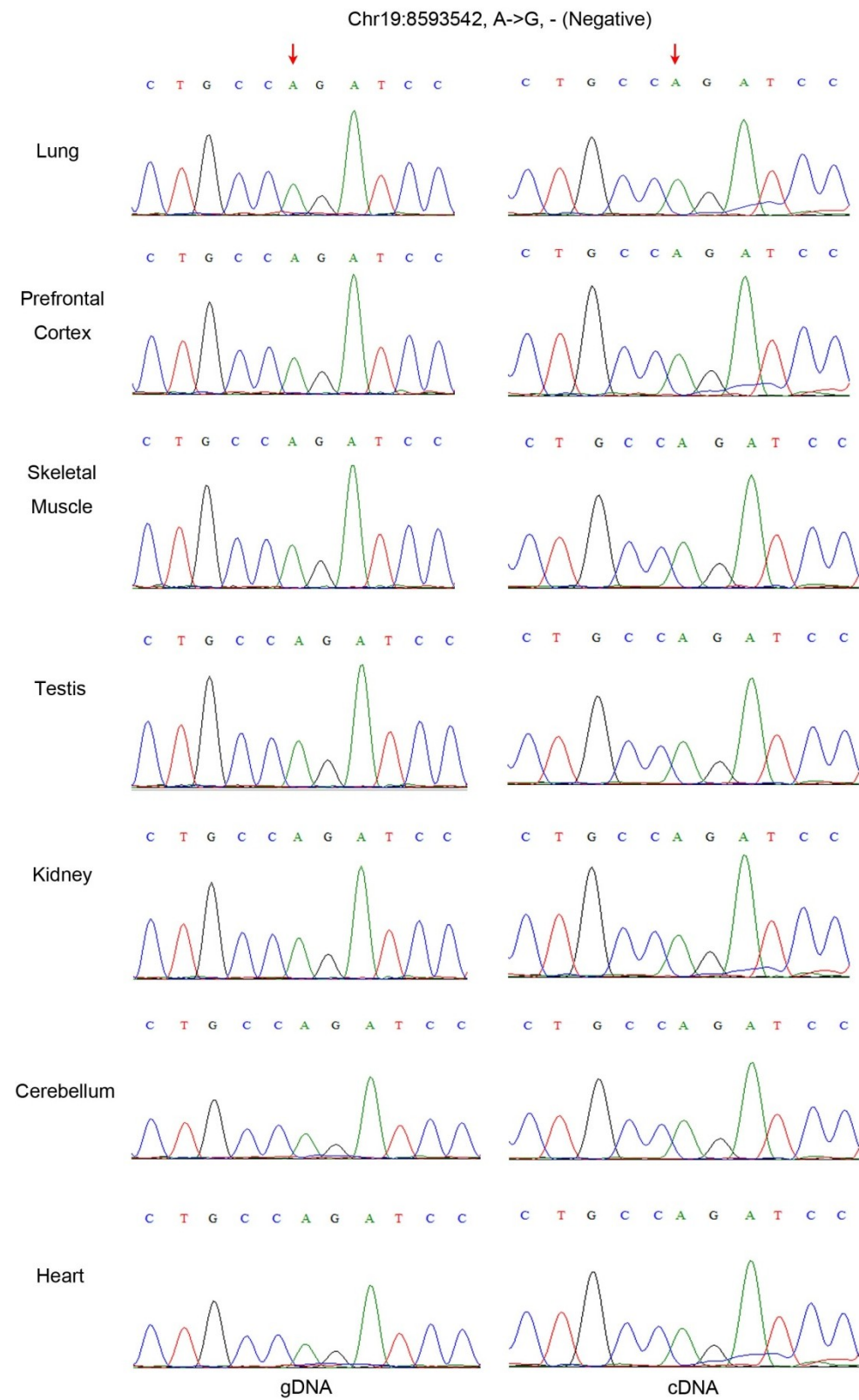

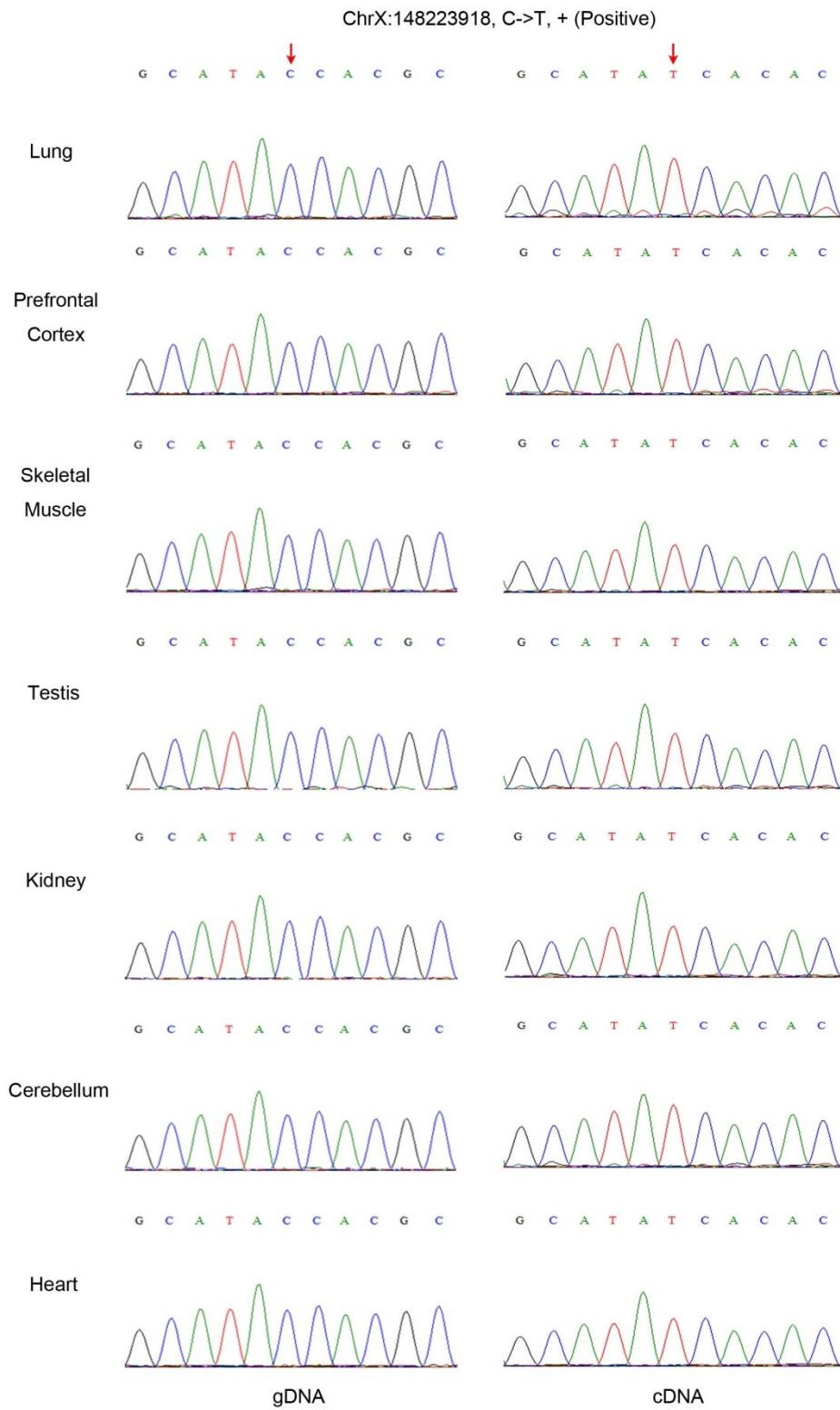

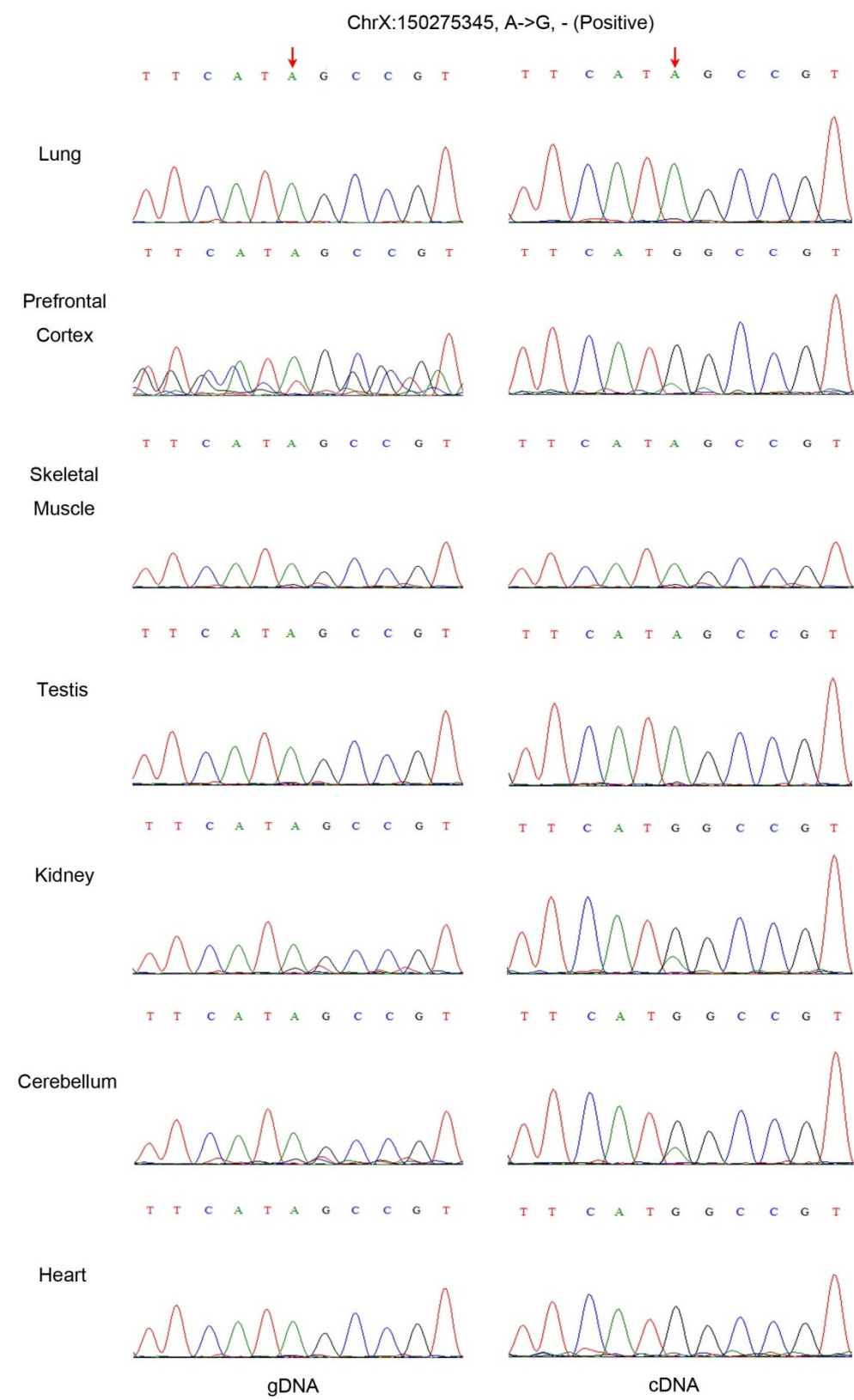

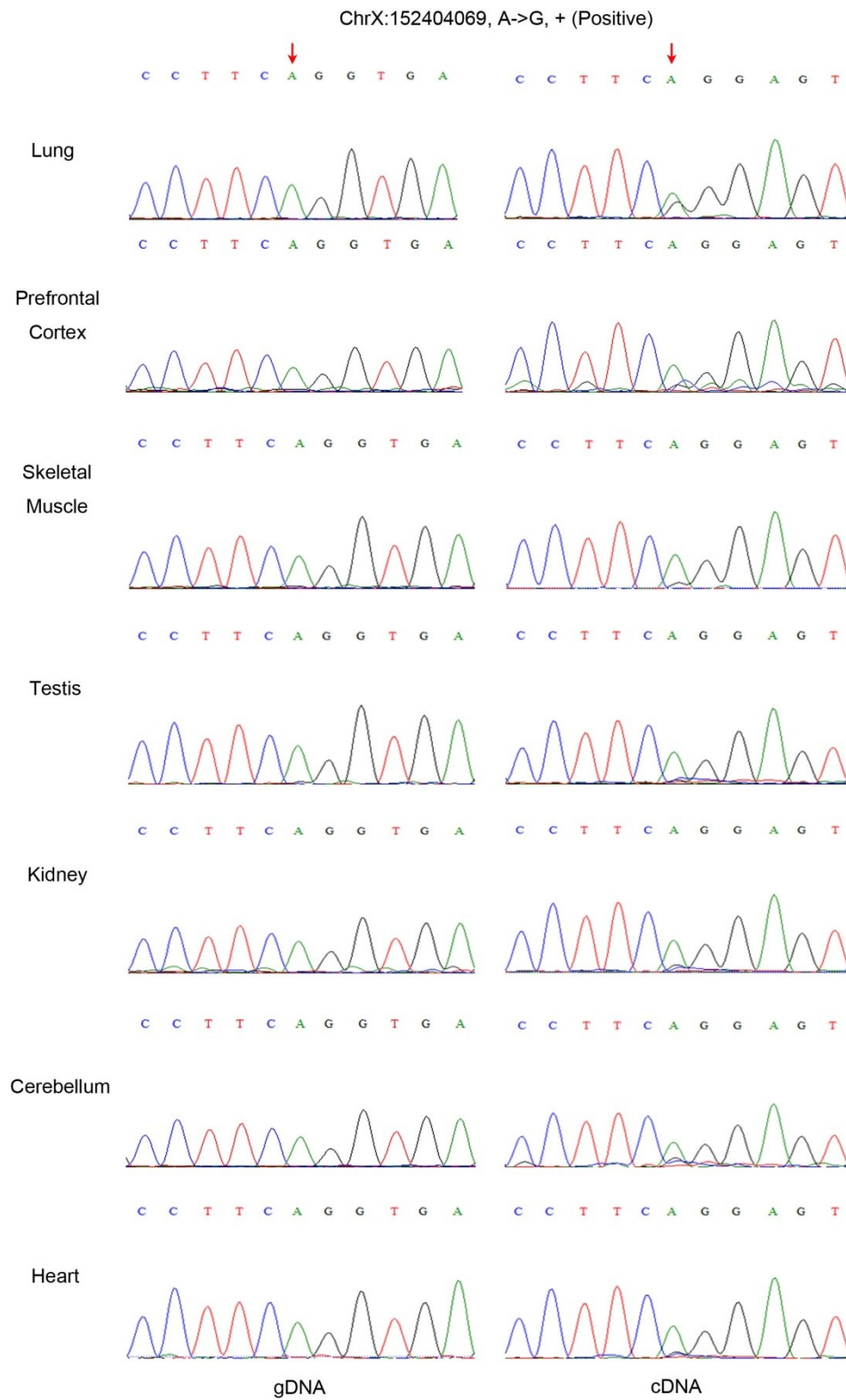

Supplement: Figure S3 — Results of Sanger sequencing validation for all 29 candidate macaque editing sites in coding regions. For each candidate editing site (indicated by genome coordinates and red arrows), raw chromatograms of sequences derived from seven cDNA and the matched DNA (gDNA) samples are shown (S3-1 to S3-27). (PDF) [file pgen.1004274.s003.pdf]

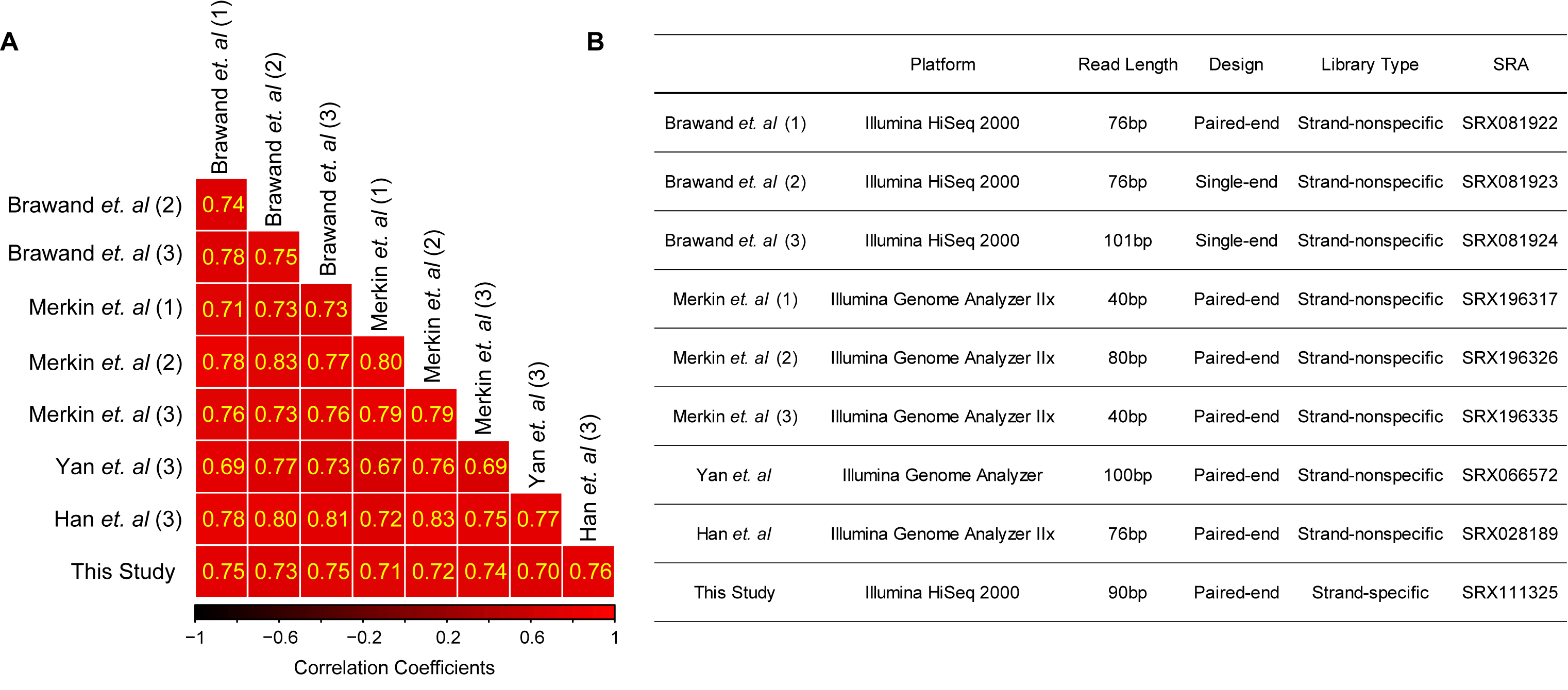

Supplement: Figure S5 — The levels of RNA editing could be accurately estimated using RNA-Seq data. For each editing site with adequate read coverage, the levels of editing were estimated using different RNA-Seq datasets of macaque brain. Pair-wise Pearson correlation coefficients are shown in (A). The basic information on these public datasets is summarized in (B). (TIF) [file pgen.1004274.s005.tif]

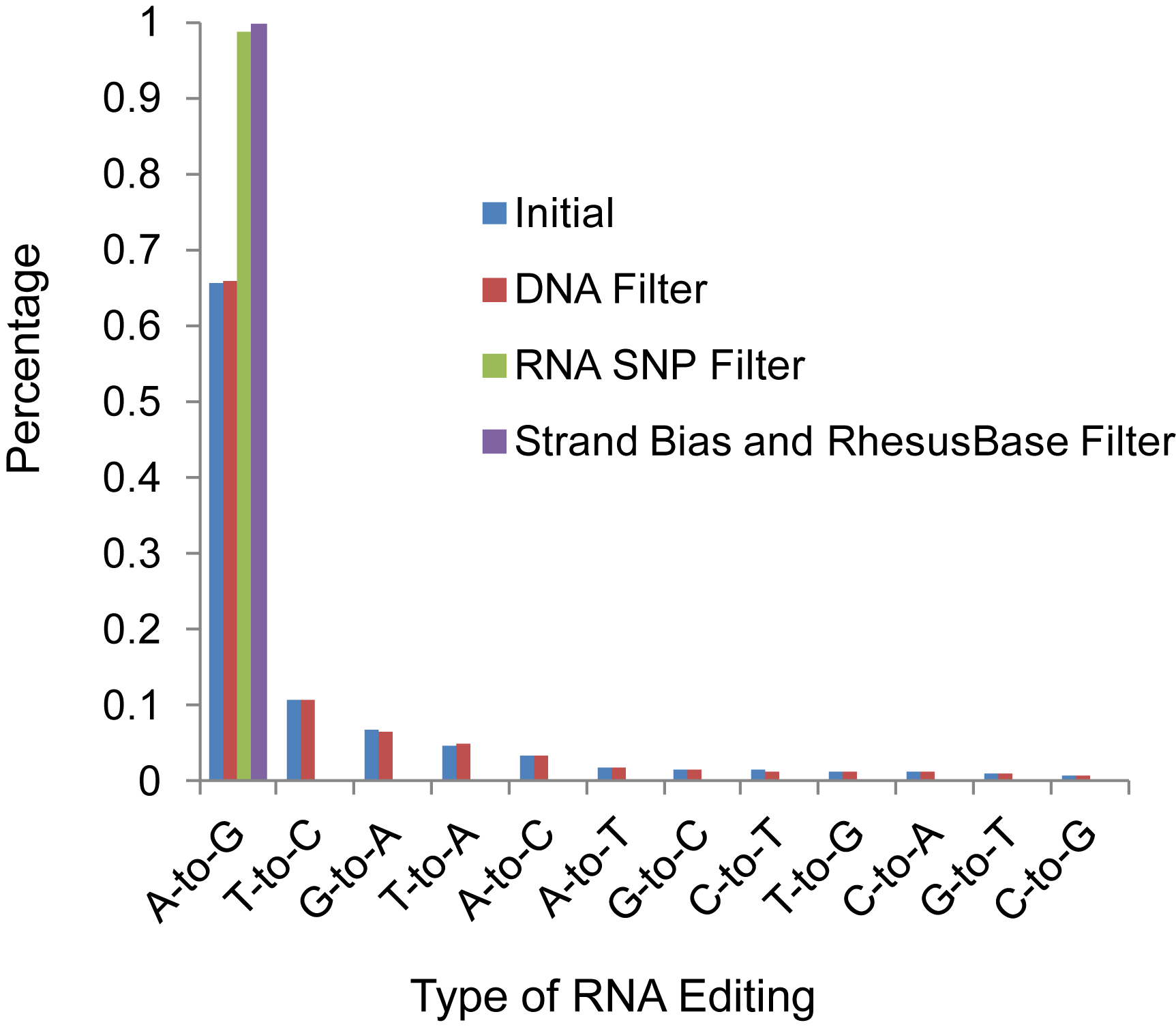

Supplement: Figure S6 — Enrichment of the A-to-G editing sites by the multi-filter strategy. Initial: initial list of macaque editing sites identified by high-throughput sequencing. Relative representation of macaque editing types for sites that progressively passed the DNA filter, the RNA SNP filter, the strand-bias and RhesusBase filter, as described in Materials and Methods . (TIF) [file pgen.1004274.s006.tif]

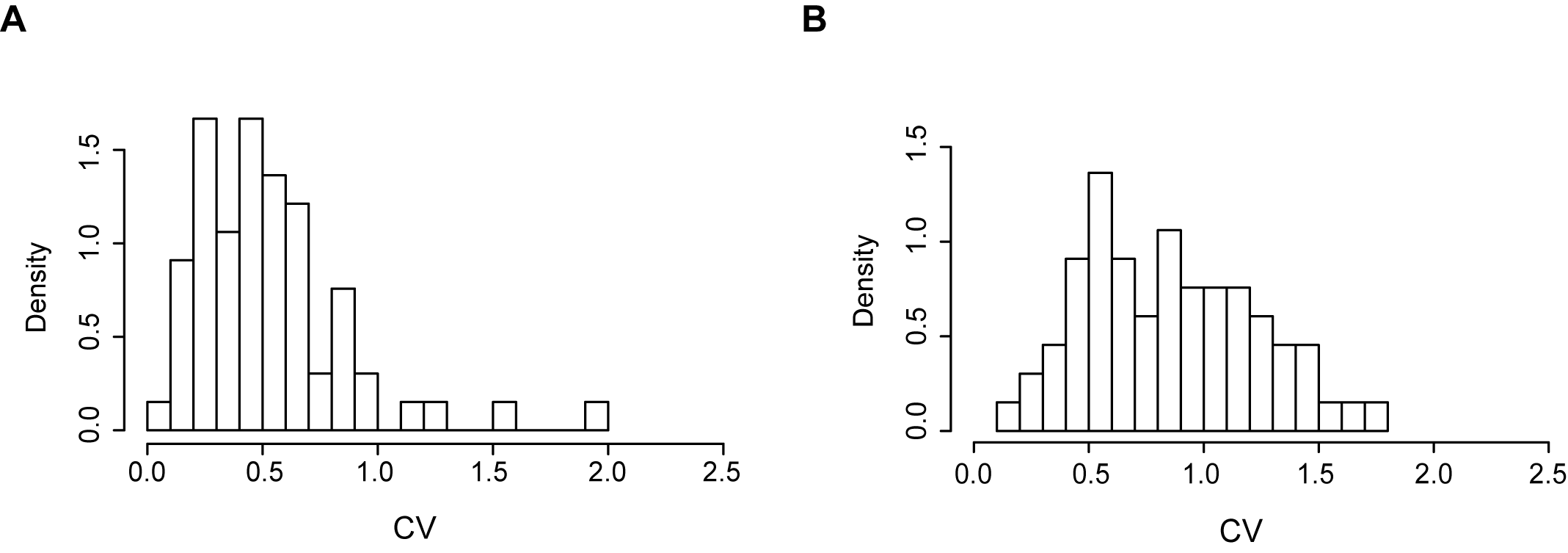

Supplement: Figure S7 — Quantitative analysis of intra-population and cross-tissue variations of editing levels. The distributions of coefficient of variance (CV) values within macaque animals (A), as well as across different tissues (B) are shown. (TIF) [file pgen.1004274.s007.tif]

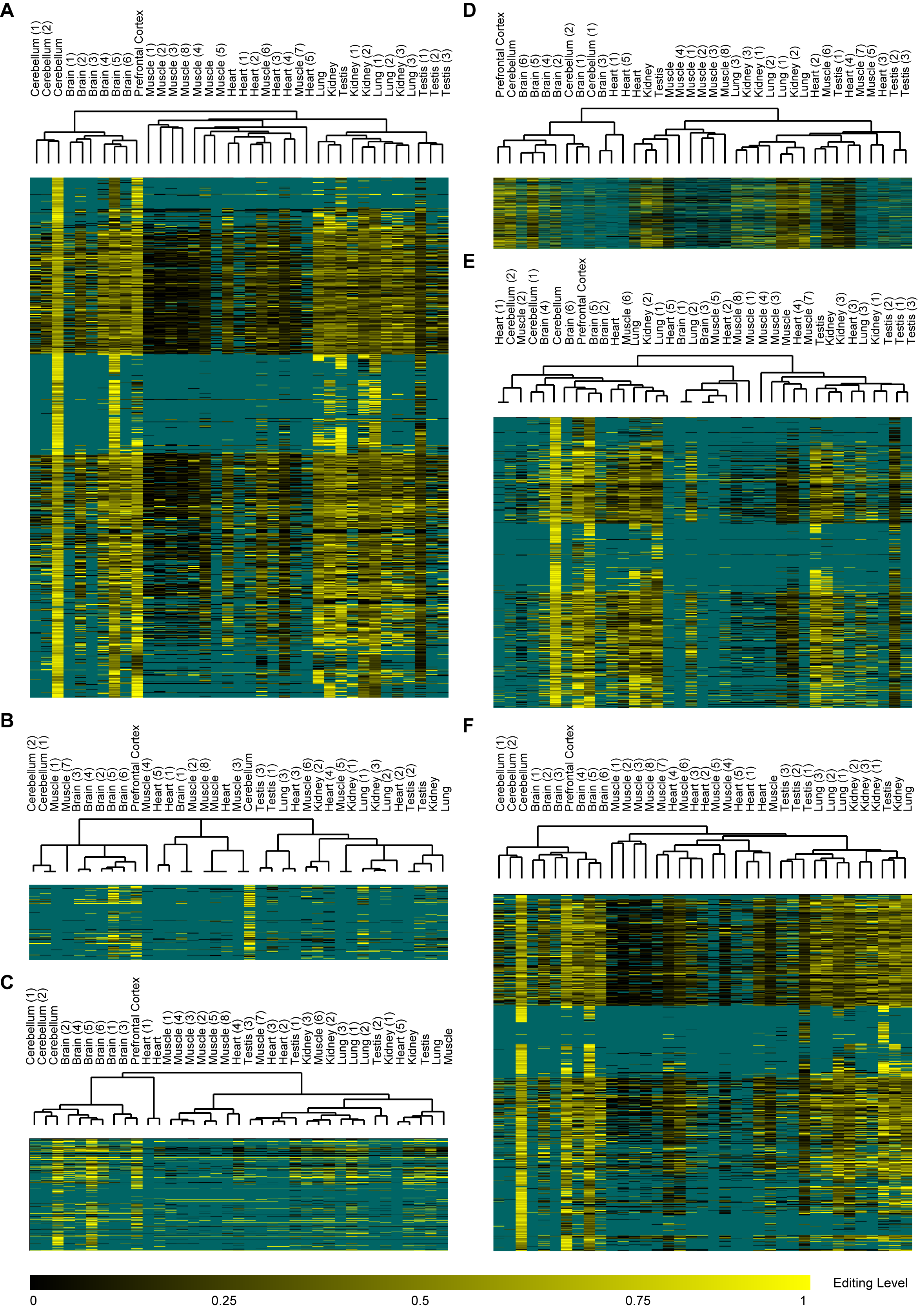

Supplement: Figure S8 — Hierarchical clustering of editing levels for subsets of editing sites. Editing levels were estimated on the basis of RNA-Seq data in this study (Testis, Lung, Kidney, Heart, Muscle, Prefrontal cortex) and other public RNA-Seq data [Brain (1–6), Cerebellum (1–2), Muscle (1–8), Heart (1–5), Kidney (1–3), Lung (1–3), Testis (1–3)], with missing data in dark cyan. Six subsets of editing sites, including sites in Alu (A), non-Alu repeat (B), non-repetitive (C), un-translated (D), intronic (E) and intergenic (F) regions were analyzed separately. (TIF) [file pgen.1004274.s008.tif]

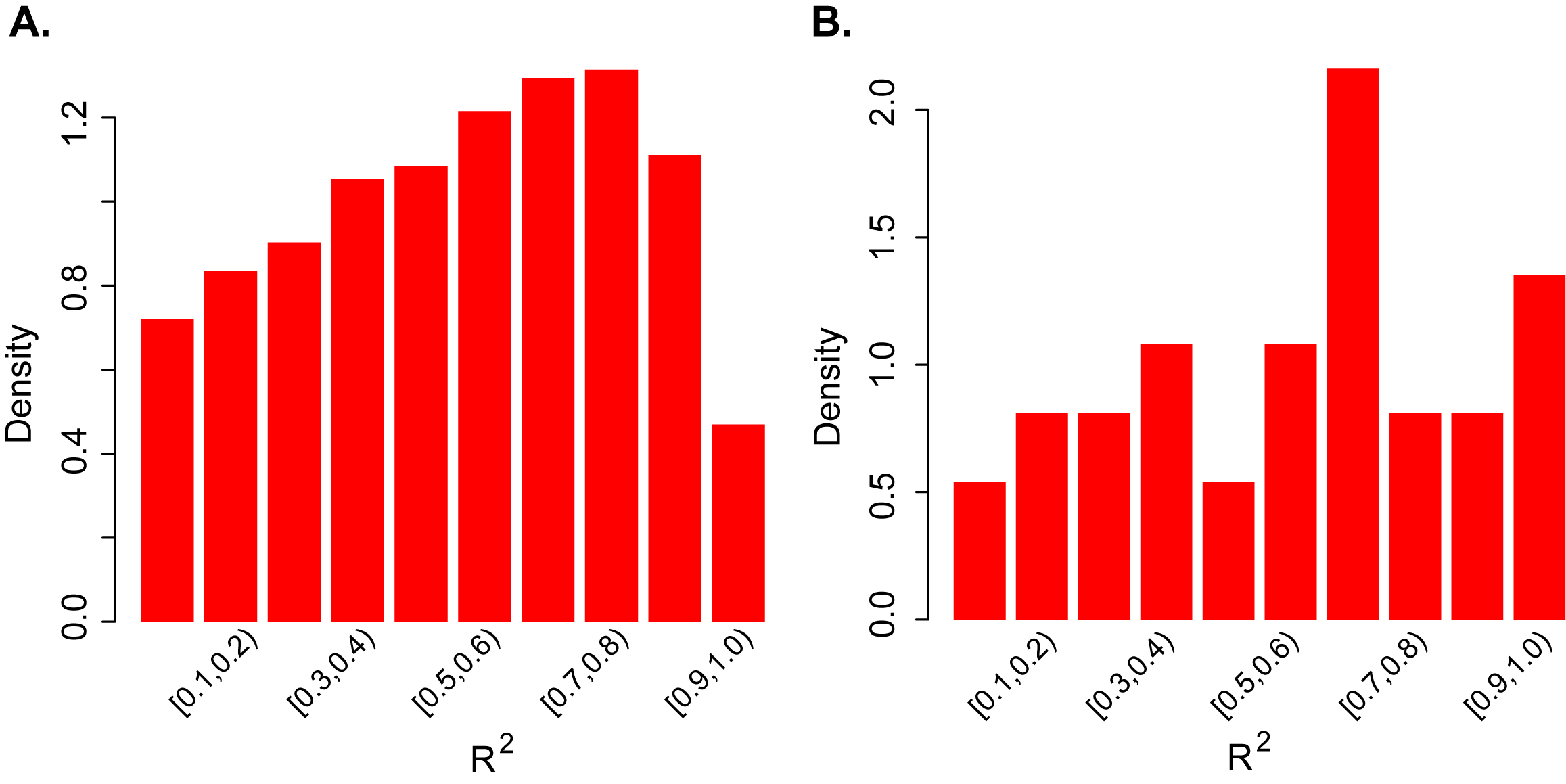

Supplement: Figure S9 — Distributions of R2 values. Distributions of R2 values in models assuming association of editing level of Alu (A) or non-Alu sites (B) with ADARs expression are shown. (TIF) [file pgen.1004274.s009.tif]

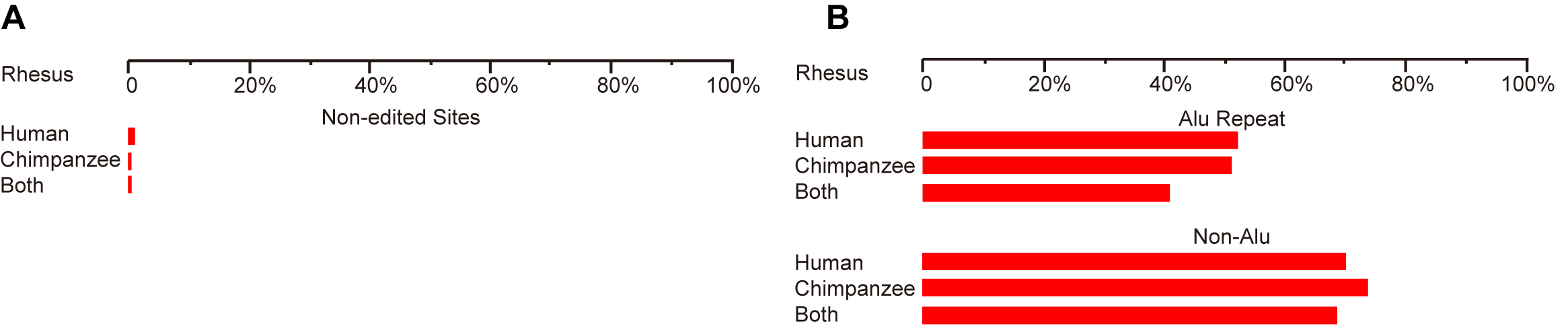

Supplement: Figure S10 — Cross-species comparisons of different subsets of editing sites. The percentages of macaque editing sites that had corresponding editing sites in human and/or chimpanzee are denoted by red bars. Comparisons were done for the background using the adjacent non-edited sites to indicate the degrees of RNA-Seq sequence errors (A), as well as for the subsets of editing sites in different genomic context (B). (TIF) [file pgen.1004274.s010.tif]

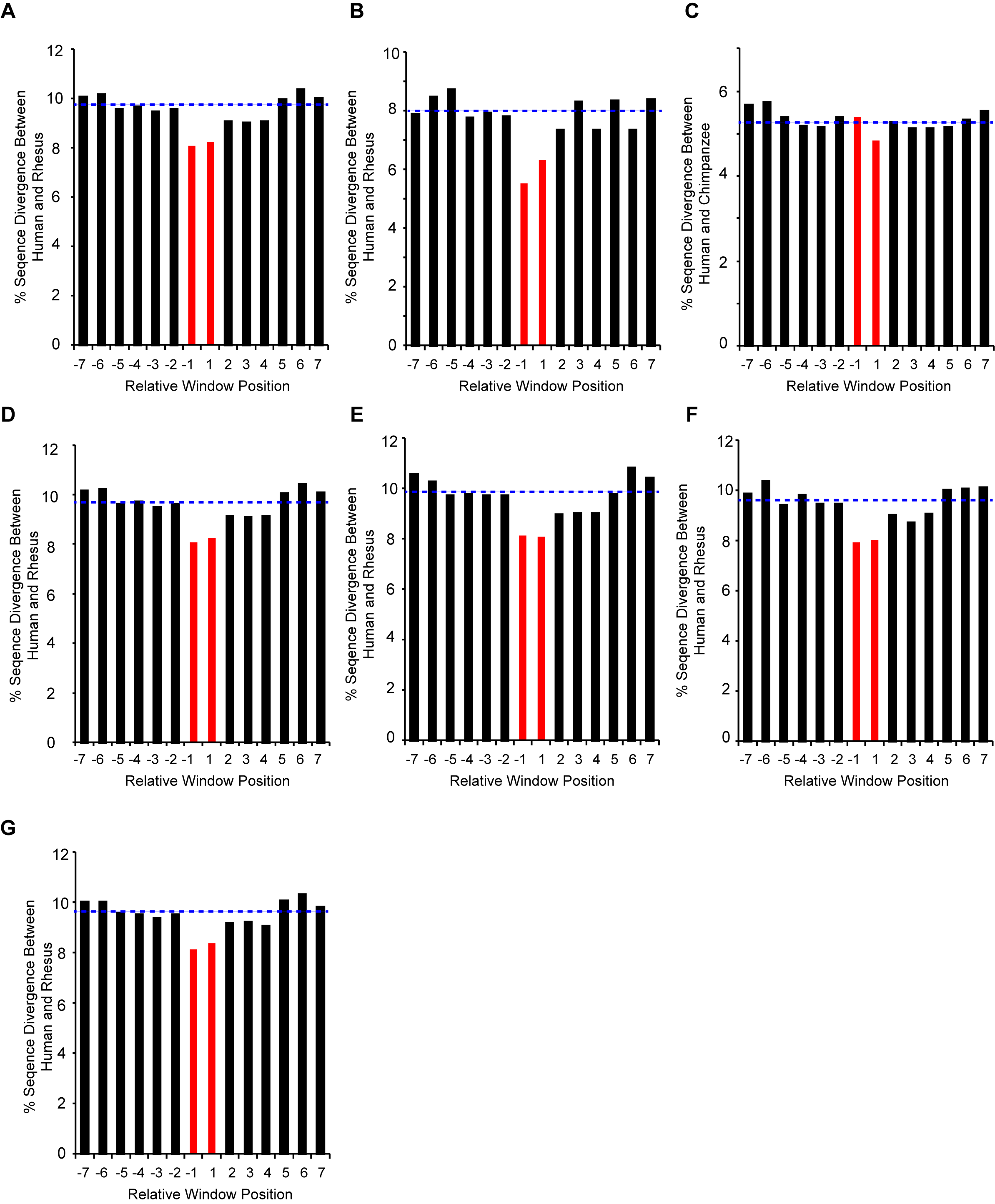

Supplement: Figure S11 — Signatures of purifying selection for editing sites in different subsets. The genomic sequences nearby the macaque editing sites were compiled according to the distances to the editing sites. For each 6-nucleotide window, the proportion of human-macaque (A, B, D, E, F & G) or human-chimpanzee (C) divergent sites is shown for different subsets of macaque editing sites. A: all macaque editing sites, B: conserved sites between human and macaque, C: non-edited sites in both human and chimpanzee, D: editing sites in Alu regions, E–G: editing sites with low, medium and high expression levels, respectively. The nearby regions are highlighted in red and the average divergence rate of the distal regions is indicated by blue dashed lines. (TIF) [file pgen.1004274.s011.tif]

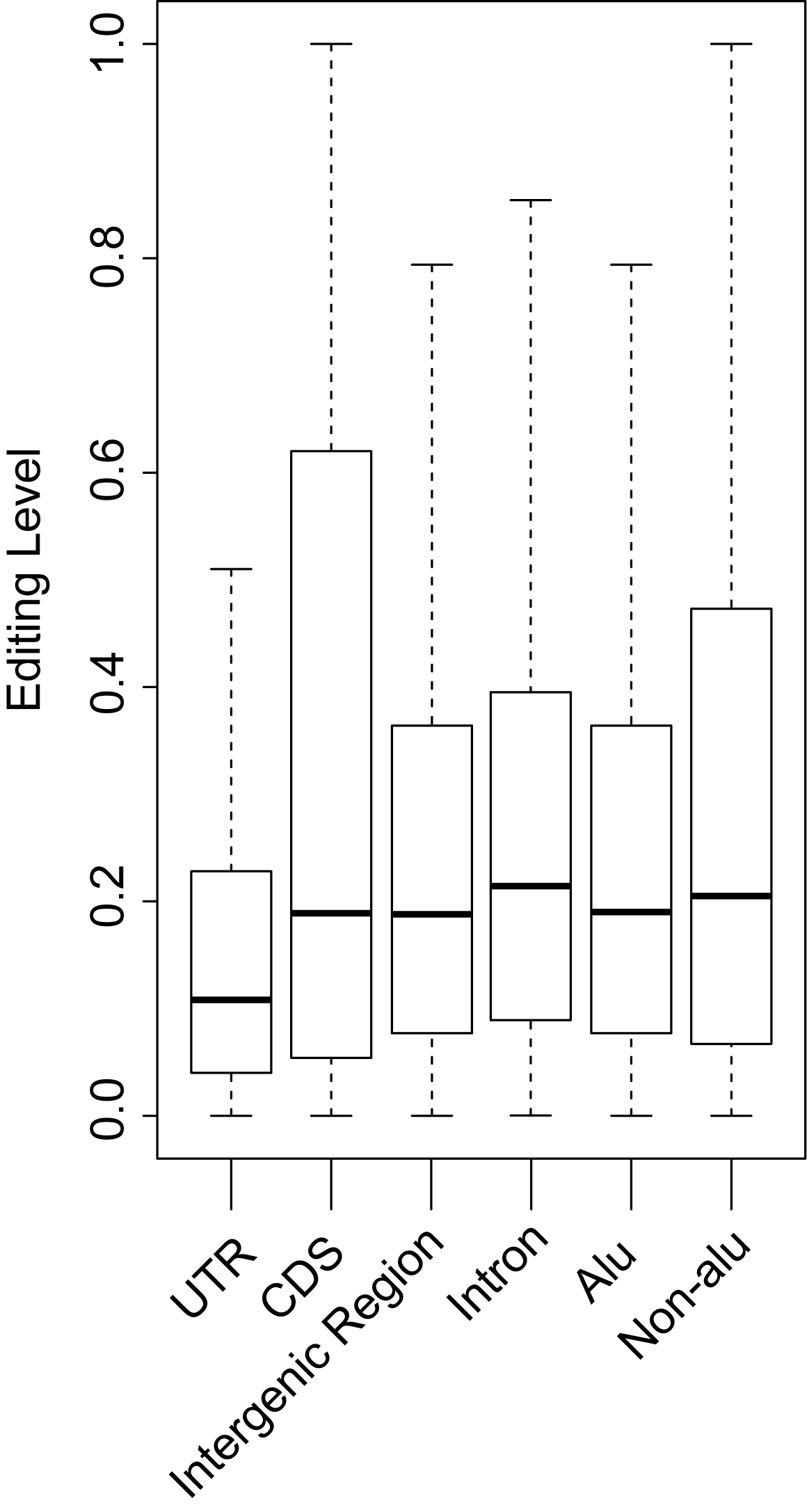

Supplement: Figure S12 — Distribution of levels of RNA editing in different genomic regions. For editing sites in untranslated, CDS, intronic and intergenic regions, the distribution of the levels of RNA editing is summarized in boxplot. (TIF) [file pgen.1004274.s012.tif]
