# Supplementary material for: RNA Editome in Rhesus Macaque Shaped by Purifying Selection
Source: PLoS Genet. 2014 Apr 10;10(4):e1004274. doi: 10.1371/journal.pgen.1004274 (PMC3983040; doi:10.1371/journal.pgen.1004274)
Supplement: Figure S4 — Results of Sanger sequencing validation for 79 candidate macaque editing sites in non-coding regions. For each candidate editing site (indicated by genome coordinates and red underlines) in intronic regions (S4-1), untranslated regions (S4-2), or intergenic regions (S4-3), raw chromatograms of sequences derived from one cDNA and the matched DNA (gDNA) samples are shown. *Sites used in regression analysis. (PDF) [file pgen.1004274.s004.pdf]

Figure S4

S4-1. Intronic Region

chr4:97883996:A-to-G:+

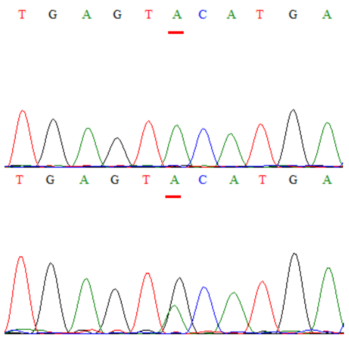

chr4:97883987:A-to-G:+

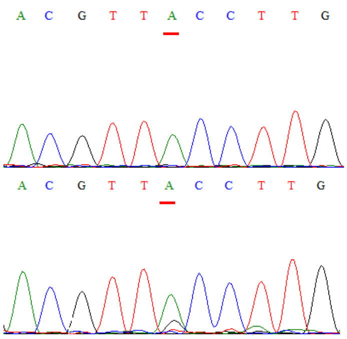

chr4:97883969:A-to-G:+

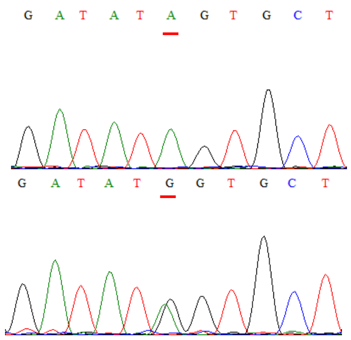

chr4:97883925:A-to-G:+

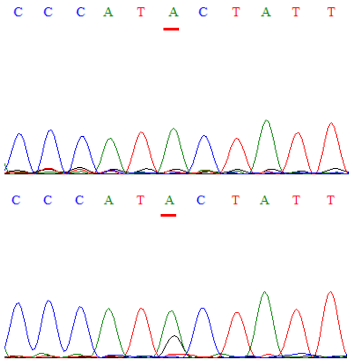

chr4:97883906:A-to-G:+

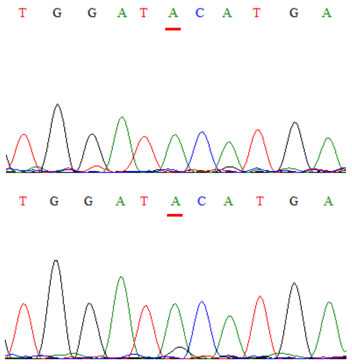

chr8:76047235:A-to-G:+

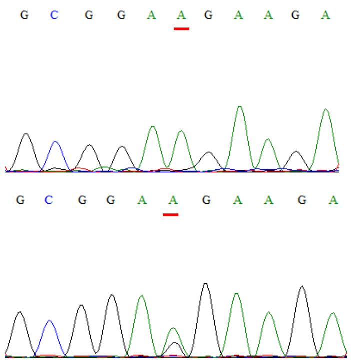

chr16: 43522348:A-to-G:+
chr16: 43522351:A-to-G:+

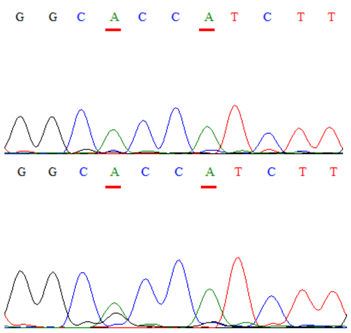

chr16: 43522387:A-to-G:+
chr16: 43522391:A-to-G:+

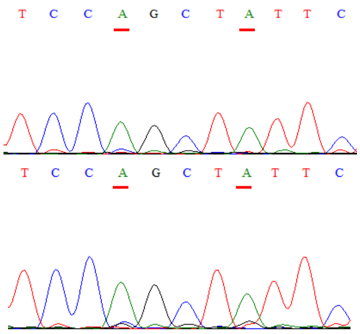

chr16: 43522467:A-to-G:+
chr16: 43522470:A-to-G:+

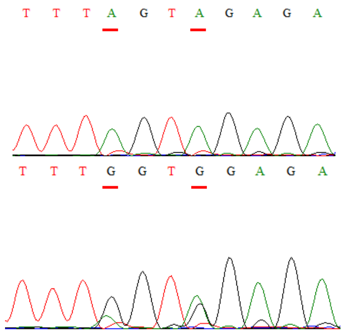

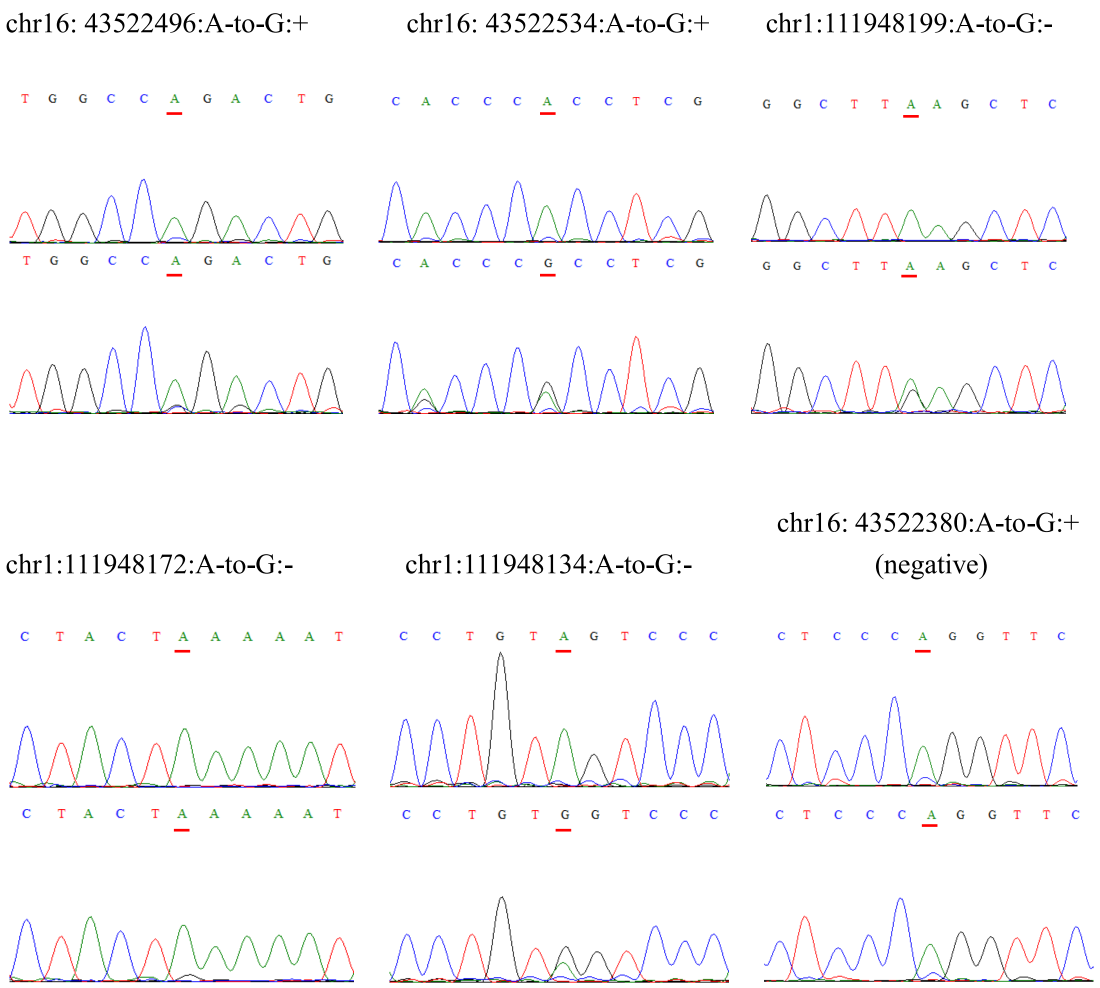
chr16: 43522496:A-to-G:+
T G G C C A G A C T G
T G G C C A G A C T G
chr16: 43522534:A-to-G:+
C A C C C A C C T C G
C A C C C G C C T C G
chr1:111948199:A-to-G:-
G G C T T A A G C T C
G G C T T A A G C T C
chr1:111948172:A-to-G:-
C T A C T A A A A A T
C T A C T A A A A A T
chr1:111948134:A-to-G:-
C C T G T A G T C C C
C C T G T G G T C C C
chr16: 43522380:A-to-G:+
(negative)
C T C C C A G G T T C
C T C C C A G G T T C

S4-2. UTR

chr6:128381178:A-to-G:-*

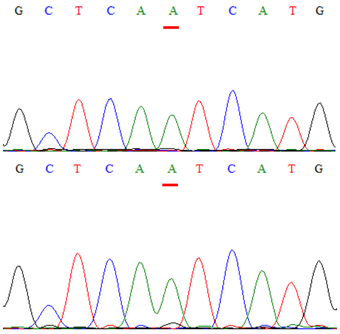

chr6:128381165:A-to-G:-*
chr6:128381163:A-to-G:-*

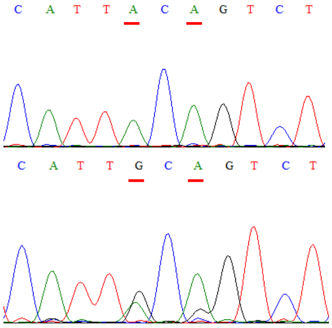

chr6:128381143:A-to-G:-*

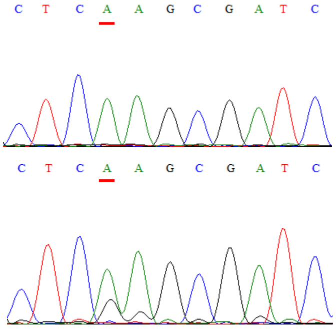

chr6:128381118:A-to-G:-*
chr6:128381117:A-to-G:-*
chr6:128381114:A-to-G:-*

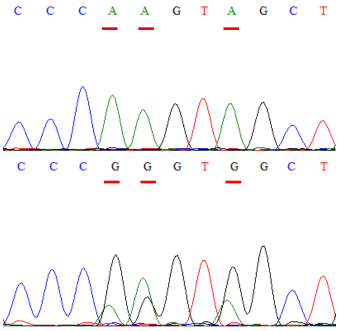

chr6:128381104:A-to-G:-*
chr6:128381102:A-to-G:-*
chr6:128381096:A-to-G:-*

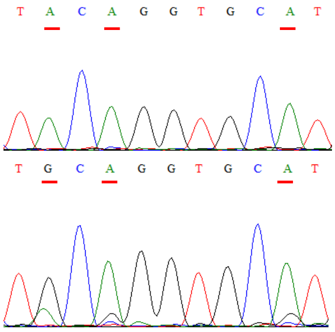

chr6:128381035:A-to-G:-*

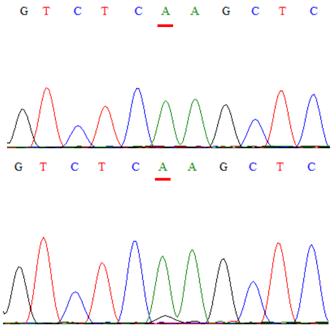

chr6:128380993:A-to-G:-*
chr6:128380987:A-to-G:-*

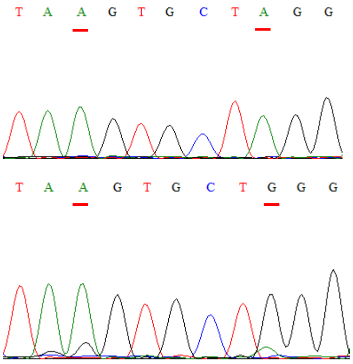

chr6:128380981:A-to-G:-*
chr6:128380975:A-to-G:-*

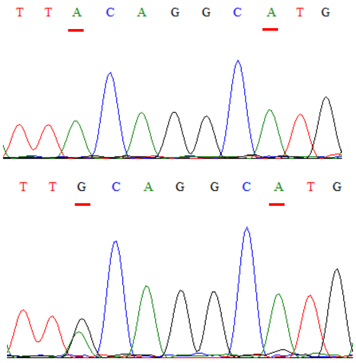

chr19:63670112:A-to-G:+

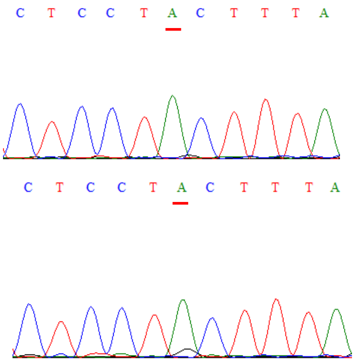

chr19:63670139:A-to-G:+

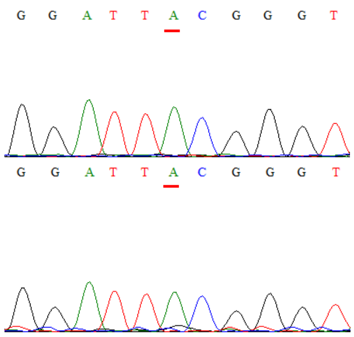

chr19:63670207:A-to-G:+

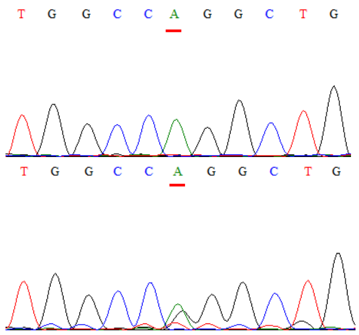

chr19:63670226:A-to-G:+

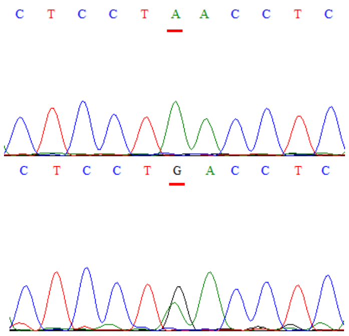

chr19:63670259:A-to-G:+
chr19:63670260:A-to-G:+
chr19:63670267:A-to-G:+

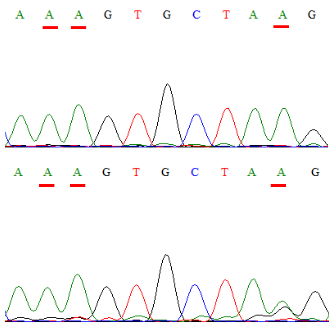

chr8: 39450062:A-to-G:+

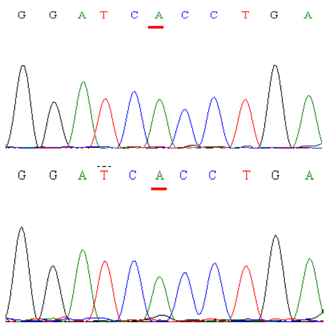

chr8: 39450072:A-to-G:+

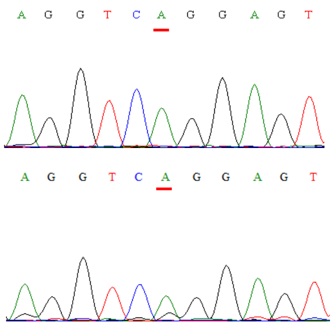

chr8:39450228:A-to-G:+

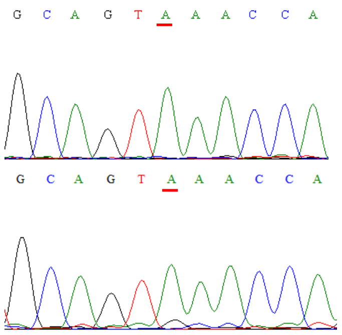

chr8:39450205:A-to-G:+

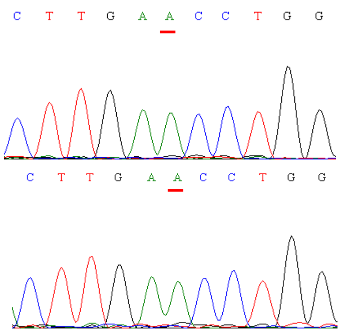

chr8:39450177:A-to-G:+

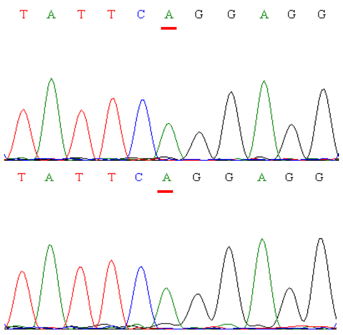

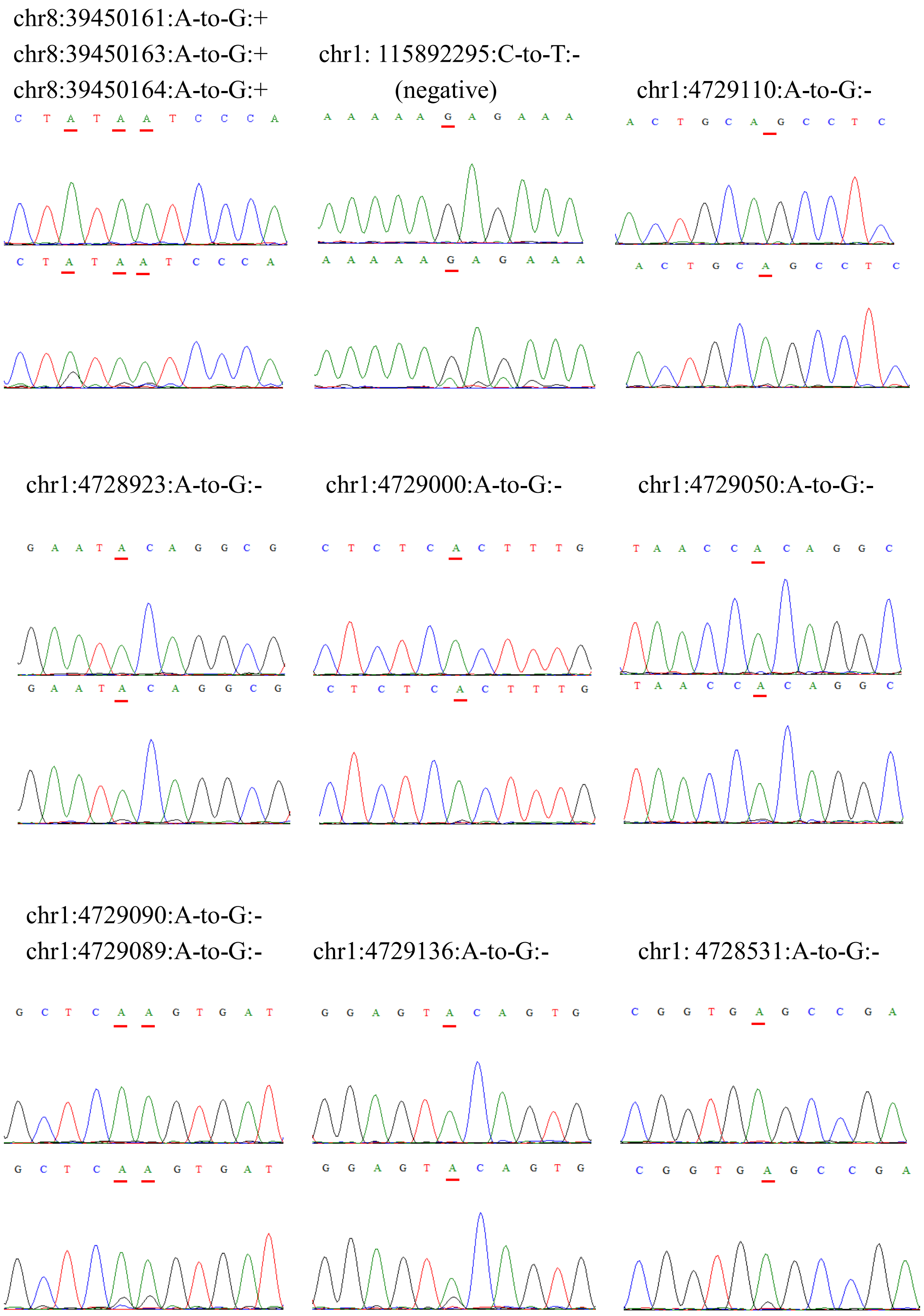

chr8:39450161:A-to-G:+
chr8:39450163:A-to-G:+
chr8:39450164:A-to-G:+
C T A T A A T C C C A
C T A T A A T C C C A
chr1: 115892295:C-to-T:-
(negative)
A A A A A G A G A A A
A A A A A G A G A A A
chr1:4729110:A-to-G:-
A C T G C A G C C T C
A C T G C A G C C T C
chr1:4728923:A-to-G:-
G A A T A C A G G C G
G A A T A C A G G C G
chr1:4729000:A-to-G:-
C T C T C A C T T T G
C T C T C A C T T T G
chr1:4729050:A-to-G:-
T A A C C A C A G G C
T A A C C A C A G G C
chr1:4729090:A-to-G:-
chr1:4729089:A-to-G:-
G C T C A A G T G A T
G C T C A A G T G A T
chr1:4729136:A-to-G:-
G G A G T A C A G T G
G G A G T A C A G T G
chr1: 4728531:A-to-G:-
C G G T G A G C C G A
C G G T G A G C C G A

chr1: 4728555:A-to-G:-

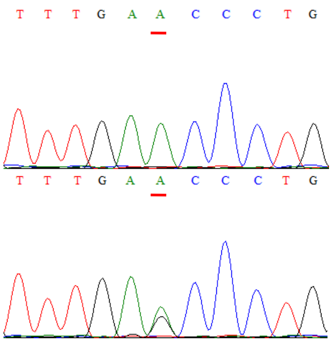

chr1: 4728570:A-to-G:-

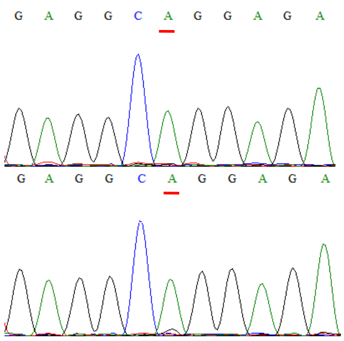

chr1: 4728583:A-to-G:-

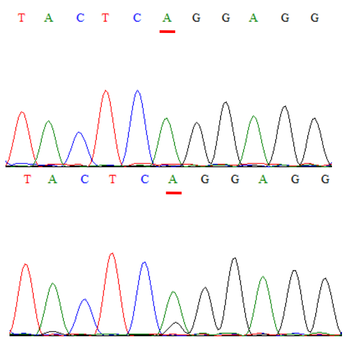

chr1: 4728597:A-to-G:-

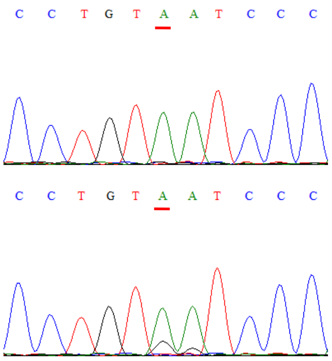

chr1: 4728628:A-to-G:-
chr1: 4728627:A-to-G:-

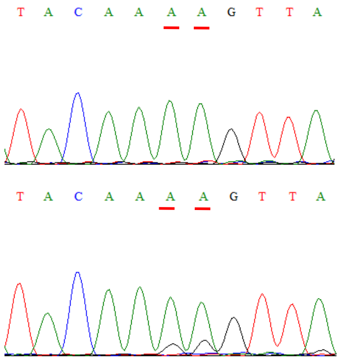

chr1:4728737:A-to-G:-

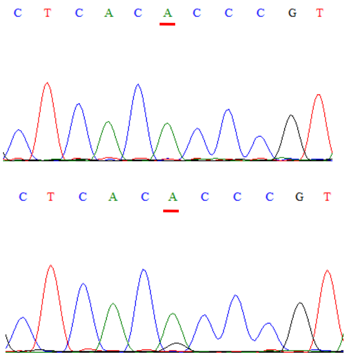

S4-3. Intergenic Regions

chr6:44916092:A-to-G:+
chr6:44916093:A-to-G:+
chr6:44916100:A-to-G:+

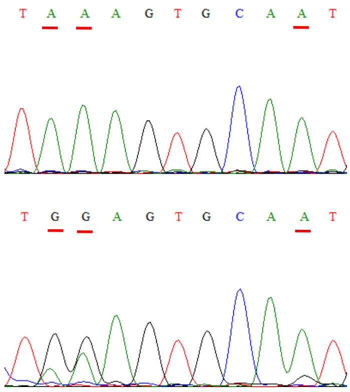

chr6:44916227:A-to-G:+
chr6:44916228:A-to-G:+

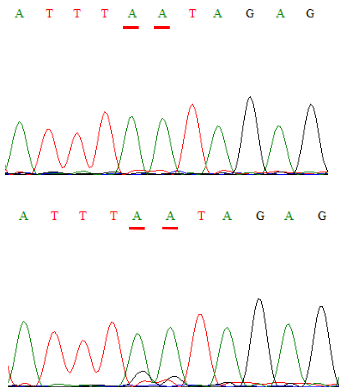

chr6:44916244:A-to-G:+

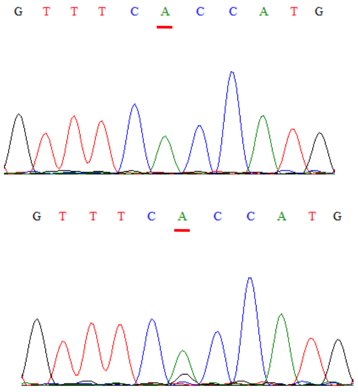

chr6:44916261:A-to-G:+

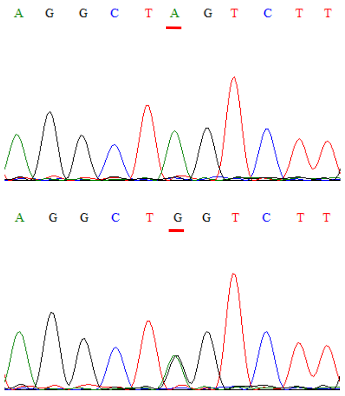

chr6:146462770:A-to-G:+
chr6:146462771:A-to-G:+

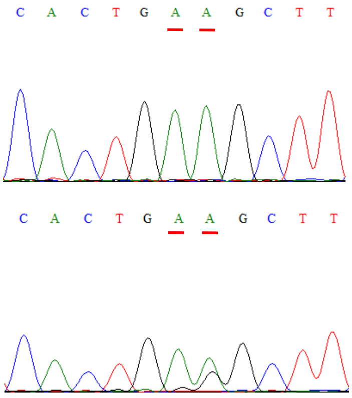

chr6:146462792:A-to-G:+

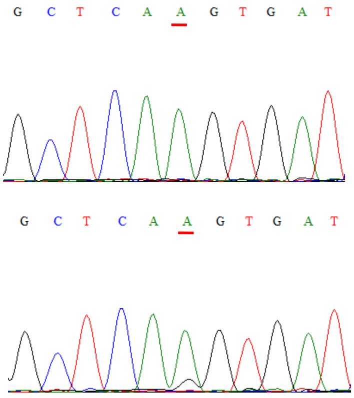

chr6: 146462804:A-to-G:+

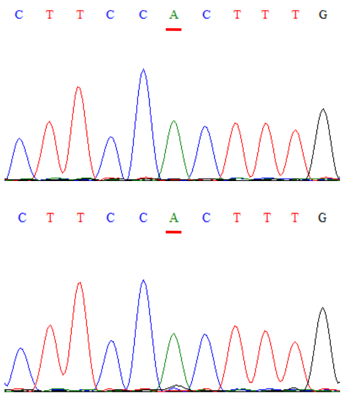

chr6:146462864:A-to-G:+

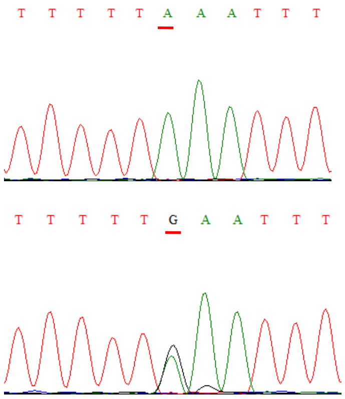

chr6:146462894:A-to-G:+

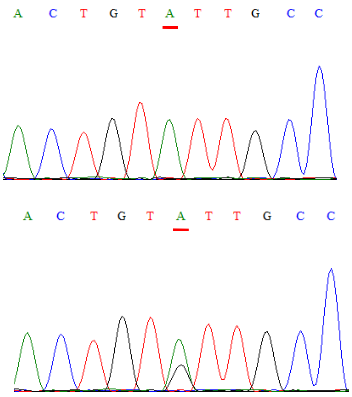

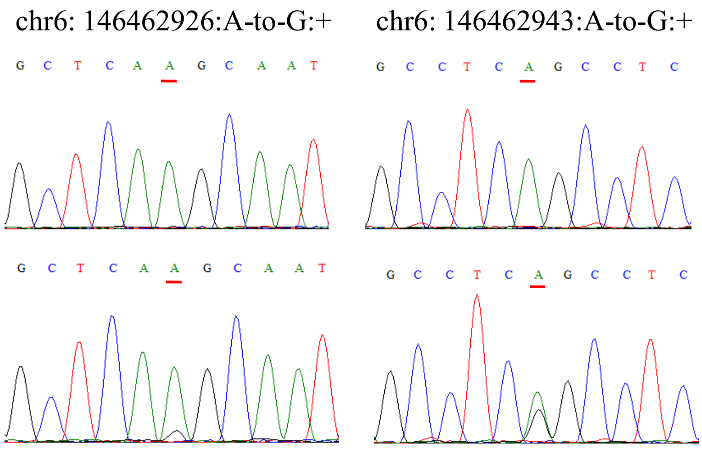
chr6: 146462926:A-to-G:+
chr6: 146462943:A-to-G:+
G C T C A A G C A A T
G C C T C A G C C T C
G C T C A A G C A A T
G C C T C A G C C T C
