# Supplementary material for: RNA Editome in Rhesus Macaque Shaped by Purifying Selection
Source: PLoS Genet. 2014 Apr 10;10(4):e1004274. doi: 10.1371/journal.pgen.1004274 (PMC3983040; doi:10.1371/journal.pgen.1004274)
Supplement: Table S2 — Primers used in Sanger sequencing. (PDF) [file pgen.1004274.s014.pdf]

Table S2. Primers used in Sanger sequencing

Position	DNA Primer	Product Length	RNA Primer	Product Length
chr1:138950859	TTCAACACCCTCAGAAACAA,<br>TGTTGACTTCCACCTCTAAT	302	TGCCTTTGATTCATTCATGC,<br>GATCAGACCATCCGAGTGTG	378
chr6:129544096	TGTGGTTTTTCCCACCTCTC,<br>TGTTTCCATTGCTTCCCAAT	537	GGTACTGCCAGTCGATCCAT,<br>GGTGCAACGAGGTCTTCTGT	471
chr6:38781096	TCGTCTGCTACCAGGAAACC,<br>TTTCTCGCTTCTGCAAGGAT	641	CCCGATTAACACCAAGCAGGATCG,<br>CCACCGGGAGAGCAAGACCG	249
chr7:89331048	TATTGAGCAGCCTGTGTTGC,<br>ACAGGCAATGACCTGGTAGC	536	TATTGAGCAGCCTGTGTTGC,<br>ACAGGCAATGACCTGGTAGC	536
chrX:150275345	TAGACTGCCCACTCCTGGTC,<br>GTCTTCTGCCAAGTGGGATG	503	GTTGAAGGTGGTGCTGGTTT,<br>CCCATGCATTTGGAAGATTT	618
chr10:26945910	CTGACAGCCAGAGAGCACAG,<br>CGGGATTTGAAACTCCAATG	501	CTGACAGCCAGAGAGCACAG,<br>CGGGATTTGAAACTCCAATG	501
chr10:26945919	CTGACAGCCAGAGAGCACAG,<br>CGGGATTTGAAACTCCAATG	501	CTGACAGCCAGAGAGCACAG,<br>CGGGATTTGAAACTCCAATG	501
chr10:26945949	CTGACAGCCAGAGAGCACAG,<br>CGGGATTTGAAACTCCAATG	501	CTGACAGCCAGAGAGCACAG,<br>CGGGATTTGAAACTCCAATG	501
chr11:5028364	ACCGAGATAGCTGAGCAGGA,<br>TTTGGTTAGCTGTGGTGCAA	640	CTCTAAGGGCCTCCAGATCC,<br>GTGGTGCAATTGCCAGTTCT	516
chr12:73754116	CCAGACATTACATGCCTTCAGA,<br>TCCTGAAGTTGTGGCAGGAC	530	CCAGACATTACATGCCTTCAGA,<br>TCCTGAAGTTGTGGCAGGAC	530
chr17:24407704	CCAGGTCAGGATCGTGCTTA,<br>CGTTAGCATGGTGACAGCTC	652	TCACAGGAAGCATTGTCTGC,<br>CTCCTTGACTGGCCATTGTT	448
chr5:72269698	CTCTTCCTCCTCTTCGGACA,<br>CTTGGGTAGGTGGTGCCATC	315	CTCTTCCTCCTCTTCGGACA,<br>CTTGGGTAGGTGGTGCCATC	315
chr6:153684514	AGCATCTCACGAGCTGTGTT,<br>TATTCTGATGGAATCCACCT	250	CTGGCTGACATTGTCAATAT,<br>CCACCTCACTGTTGCTGTAG	403
chrX:152404069	CTGTGGTGTGGCTTATGTGG,<br>ACGTGGTGGGCAGGATATT	519	CTGTGGTGTGGCTTATGTGG,<br>TCAGGTAAACGCCATTCTCC	329
chr11:47207718	CCACCACCTTCTCTCCTTTG,<br>GGTGGCAGCAGAATGTAGGT	544	TGCTCACTTCTGTCCCATGA,<br>AATTCAGGCTGGGAGCTAGG	646
chr7:54257914	ATCACCAGAGGAGGGAGAGT,<br>TGTTTGCCTGTGTGAACAGT	299	TACGAAACCTAGCGGACAAG,<br>GTCAGAGTCCACATCAGCAA	491
chr7:54257915	ATCACCAGAGGAGGGAGAGT,<br>TGTTTGCCTGTGTGAACAGT	299	TACGAAACCTAGCGGACAAG,<br>GTCAGAGTCCACATCAGCAA	491
chr5:149561914	GCCTCAGAAGTCCAAACCAG,<br>CCAAGAAGGCATGGAATGAT	538	CAGCAGATTTAGCCCCTACGAGTG,<br>TTAGGTGTTGCGATGCCATAGCC	585
chr5:149561918	GCCTCAGAAGTCCAAACCAG,<br>CCAAGAAGGCATGGAATGAT	538	CAGCAGATTTAGCCCCTACGAGTG,<br>TTAGGTGTTGCGATGCCATAGCC	585
chr1:134852231	CCCCTTTCTTCTCTTCCTGA,<br>GAATAGCAGGGTCAGGGTTC	249	CCCCTTTCTTCTCTTCCTGA,<br>GAATAGCAGGGTCAGGGTTC	249

chr3:13010093	CCGATGTTGTCACCACAGAC, TGCGGTAACAGTAGCAATGG	503	ATCGTTGACAGCTCCGATGT, AGCAATGGAGTTGACCGAAC	504
chr7:43601461	GACAGCCGAAAGGAGCA, GGAGGGACAAGTCTACAGAC	194	CTTCAGATAGTACGCCATCAGC, TCATGAGGAACCACTCAGATTC	292
chr2:78236903	GTGACTGGGTGACATCTGTG, CTGGGTTCAGAGTCTCTGAA	297	CCATTCTCATGAGGGATGAA, TGACCATAAAAATGGGTCGT	337
chrX:148223918	TGTCAGTATGGAGTCAGTTT, TTTGCTTCTGGCATCATTAC	441	TGTCAGTATGGAGTCAGTTT, TTTGCTTCTGGCATCATTAC	441
chr19:8593542	TGCATTCAGAATCTTCAGTCAG, CTTTCTGTTGGAGCCCAACT	370	GCAGGCTGTGAACAGGGGAG, TGGGTGAGGCCACCTTGTAG	332
chr4:33833717	CATGAATTCCATCCGCATAG, CTTGAAGAGGCGAAGGATGT	403	CATGAATTCCATCCGCATAG, CTTGAAGAGGCGAAGGATGT	403
chr4:44072645	TGAGCCTCCTCTCAGCCATAG, CCTGCCTTCCTAGAGTGGAGA	374	AGGCAGCTATTCGGTTTGAG, ATCTTCTTGTCCAGCTTGGC	370
chr8:9841193	TGCCAGCACTGTGTTGTGTC, GGTATCGCTTTCTTGGCATGTC	363	TGCCAGCACTGTGTTGTGTC, GGTATCGCTTTCTTGGCATGTC	363
chr5:149585132	ATGTGGACCTACATGAGGAGTG, GCAAACCAAACAAAACAAAATCC	426	ATGTGGACCTACATGAGGAGTGC, GGATGTAGAATACTCCAGCAACG	388
chr8: 39450062	CTTGTGTGCTTGCATGATCAG, TCAAATGGAAGGAGGTACGC	541	CTTGTGTGCTTGCATGATCAG, TCAAATGGAAGGAGGTACGC	541
chr8:39450072	CTTGTGTGCTTGCATGATCAG, TCAAATGGAAGGAGGTACGC	541	CTTGTGTGCTTGCATGATCAG, TCAAATGGAAGGAGGTACGC	541
chr8:39450161	CTTGTGTGCTTGCATGATCAG, TCAAATGGAAGGAGGTACGC	541	CTTGTGTGCTTGCATGATCAG, TCAAATGGAAGGAGGTACGC	541
chr8:39450163	CTTGTGTGCTTGCATGATCAG, TCAAATGGAAGGAGGTACGC	541	CTTGTGTGCTTGCATGATCAG, TCAAATGGAAGGAGGTACGC	541
chr8:39450164	CTTGTGTGCTTGCATGATCAG, TCAAATGGAAGGAGGTACGC	541	CTTGTGTGCTTGCATGATCAG, TCAAATGGAAGGAGGTACGC	541
chr8:39450177	CTTGTGTGCTTGCATGATCAG, TCAAATGGAAGGAGGTACGC	541	CTTGTGTGCTTGCATGATCAG, TCAAATGGAAGGAGGTACGC	541
chr8:39450205	CTTGTGTGCTTGCATGATCAG, TCAAATGGAAGGAGGTACGC	541	CTTGTGTGCTTGCATGATCAG, TCAAATGGAAGGAGGTACGC	541
chr8:39450228	CTTGTGTGCTTGCATGATCAG, TCAAATGGAAGGAGGTACGC	541	CTTGTGTGCTTGCATGATCAG, TCAAATGGAAGGAGGTACGC	541
chr1: 115892295	ATTCTGGTCCACCTTCCCTC, CCAGAAAAATTTGGCACCAGAC	247	ATTCTGGTCCACCTTCCCTC, CCAGAAAAATTTGGCACCAGAC	247
chr8: 76047235	TCCCTAATTCCTTCAGTCAGAAC, AGTTAATCTGCCCATGGAATGC	441	TCCCTAATTCCTTCAGTCAGAAC, AGTTAATCTGCCCATGGAATGC	441
chr4: 97883996	TATGCTCATAGGACAAACTTGC, CCAATAAAGTCAAACAAGACTGG	399	TATGCTCATAGGACAAACTTGC, CCAATAAAGTCAAACAAGACTGG	399
chr4:97883987	TATGCTCATAGGACAAACTTGC, CCAATAAAGTCAAACAAGACTGG	399	TATGCTCATAGGACAAACTTGC, CCAATAAAGTCAAACAAGACTGG	399
chr4:97883969	TATGCTCATAGGACAAACTTGC, CCAATAAAGTCAAACAAGACTGG	399	TATGCTCATAGGACAAACTTGC, CCAATAAAGTCAAACAAGACTGG	399
chr4:97883925	TATGCTCATAGGACAAACTTGC,	399	TATGCTCATAGGACAAACTTGC,	399

	CCAATAAAGTCAAACAAGACTGG		CCAATAAAGTCAAACAAGACTGG	
chr4:97883906	TATGCTCATAGGACAAACTTGC,<br>CCAATAAAGTCAAACAAGACTGG	399	TATGCTCATAGGACAAACTTGC,<br>CCAATAAAGTCAAACAAGACTGG	399
chr19: 63670112	TGAATGTGGGAAATCCTTCG,<br>GCGTGAACCACATCATTCTC	517	TGAATGTGGGAAATCCTTCG,<br>GCGTGAACCACATCATTCTC	517
chr19:63670139	TGAATGTGGGAAATCCTTCG,<br>GCGTGAACCACATCATTCTC	517	TGAATGTGGGAAATCCTTCG,<br>GCGTGAACCACATCATTCTC	517
chr19:63670207	TGAATGTGGGAAATCCTTCG,<br>GCGTGAACCACATCATTCTC	517	TGAATGTGGGAAATCCTTCG,<br>GCGTGAACCACATCATTCTC	517
chr19:63670226	TGAATGTGGGAAATCCTTCG,<br>GCGTGAACCACATCATTCTC	517	TGAATGTGGGAAATCCTTCG,<br>GCGTGAACCACATCATTCTC	517
chr19:63670259	TGAATGTGGGAAATCCTTCG,<br>GCGTGAACCACATCATTCTC	517	TGAATGTGGGAAATCCTTCG,<br>GCGTGAACCACATCATTCTC	517
chr19:63670260	TGAATGTGGGAAATCCTTCG,<br>GCGTGAACCACATCATTCTC	517	TGAATGTGGGAAATCCTTCG,<br>GCGTGAACCACATCATTCTC	517
chr19:63670267	TGAATGTGGGAAATCCTTCG,<br>GCGTGAACCACATCATTCTC	517	TGAATGTGGGAAATCCTTCG,<br>GCGTGAACCACATCATTCTC	517
chr6: 128380975	CTAGATAGTTCCTGAATATTTTAATGAGC,<br>CTTTTTGTTTGGAGACAGGG	385	CTAGATAGTTCCTGAATATTTTAATGAGC,<br>CTTTTTGTTTGGAGACAGGG	385
chr6:128380981	CTAGATAGTTCCTGAATATTTTAATGAGC,<br>CTTTTTGTTTGGAGACAGGG	385	CTAGATAGTTCCTGAATATTTTAATGAGC,<br>CTTTTTGTTTGGAGACAGGG	385
chr6:128380987	CTAGATAGTTCCTGAATATTTTAATGAGC,<br>CTTTTTGTTTGGAGACAGGG	385	CTAGATAGTTCCTGAATATTTTAATGAGC,<br>CTTTTTGTTTGGAGACAGGG	385
chr6:128380993	CTAGATAGTTCCTGAATATTTTAATGAGC,<br>CTTTTTGTTTGGAGACAGGG	385	CTAGATAGTTCCTGAATATTTTAATGAGC,<br>CTTTTTGTTTGGAGACAGGG	385
chr6:128381035	CTAGATAGTTCCTGAATATTTTAATGAGC,<br>CTTTTTGTTTGGAGACAGGG	385	CTAGATAGTTCCTGAATATTTTAATGAGC,<br>CTTTTTGTTTGGAGACAGGG	385
chr6:128381096	CTAGATAGTTCCTGAATATTTTAATGAGC,<br>CTTTTTGTTTGGAGACAGGG	385	CTAGATAGTTCCTGAATATTTTAATGAGC,<br>CTTTTTGTTTGGAGACAGGG	385
chr6:128381102	CTAGATAGTTCCTGAATATTTTAATGAGC,<br>CTTTTTGTTTGGAGACAGGG	385	CTAGATAGTTCCTGAATATTTTAATGAGC,<br>CTTTTTGTTTGGAGACAGGG	385
chr6:128381104	CTAGATAGTTCCTGAATATTTTAATGAGC,<br>CTTTTTGTTTGGAGACAGGG	385	CTAGATAGTTCCTGAATATTTTAATGAGC,<br>CTTTTTGTTTGGAGACAGGG	385
chr6:128381114	CTAGATAGTTCCTGAATATTTTAATGAGC,<br>CTTTTTGTTTGGAGACAGGG	385	CTAGATAGTTCCTGAATATTTTAATGAGC,<br>CTTTTTGTTTGGAGACAGGG	385
chr6:128381117	CTAGATAGTTCCTGAATATTTTAATGAGC,<br>CTTTTTGTTTGGAGACAGGG	385	CTAGATAGTTCCTGAATATTTTAATGAGC,<br>CTTTTTGTTTGGAGACAGGG	385
chr6:128381118	CTAGATAGTTCCTGAATATTTTAATGAGC,<br>CTTTTTGTTTGGAGACAGGG	385	CTAGATAGTTCCTGAATATTTTAATGAGC,<br>CTTTTTGTTTGGAGACAGGG	385
chr6:128381143	CTAGATAGTTCCTGAATATTTTAATGAGC,<br>CTTTTTGTTTGGAGACAGGG	385	CTAGATAGTTCCTGAATATTTTAATGAGC,<br>CTTTTTGTTTGGAGACAGGG	385
chr6:128381163	CTAGATAGTTCCTGAATATTTTAATGAGC,<br>CTTTTTGTTTGGAGACAGGG	385	CTAGATAGTTCCTGAATATTTTAATGAGC,<br>CTTTTTGTTTGGAGACAGGG	385
chr6:128381165	CTAGATAGTTCCTGAATATTTTAATGAGC,<br>CTTTTTGTTTGGAGACAGGG	385	CTAGATAGTTCCTGAATATTTTAATGAGC,<br>CTTTTTGTTTGGAGACAGGG	385

chr6:128381178	CTAGATAGTTCCTGAATATTTTAATGAGC, CTTTTTGTTTGGAGACAGGG	385	CTAGATAGTTCCTGAATATTTTAATGAGC, CTTTTTGTTTGGAGACAGGG	385
chr6: 44916092	TGTTGGTTGAATGGATGTGA, CATACATAGGAAATGCATGGAG	449	TGTTGGTTGAATGGATGTGA, CATACATAGGAAATGCATGGAG	449
chr6:44916093	TGTTGGTTGAATGGATGTGA, CATACATAGGAAATGCATGGAG	449	TGTTGGTTGAATGGATGTGA, CATACATAGGAAATGCATGGAG	449
chr6:44916100	TGTTGGTTGAATGGATGTGA, CATACATAGGAAATGCATGGAG	449	TGTTGGTTGAATGGATGTGA, CATACATAGGAAATGCATGGAG	449
chr6:44916227	TGTTGGTTGAATGGATGTGA, CATACATAGGAAATGCATGGAG	449	TGTTGGTTGAATGGATGTGA, CATACATAGGAAATGCATGGAG	449
chr6:44916228	TGTTGGTTGAATGGATGTGA, CATACATAGGAAATGCATGGAG	449	TGTTGGTTGAATGGATGTGA, CATACATAGGAAATGCATGGAG	449
chr6:44916244	TGTTGGTTGAATGGATGTGA, CATACATAGGAAATGCATGGAG	449	TGTTGGTTGAATGGATGTGA, CATACATAGGAAATGCATGGAG	449
chr6:44916261	TGTTGGTTGAATGGATGTGA, CATACATAGGAAATGCATGGAG	449	TGTTGGTTGAATGGATGTGA, CATACATAGGAAATGCATGGAG	449
chr1:111948134	GCAGGTGTCTGTCAGGATTC, AATGATACCAGTCAGGAATAAAGTC	537	GCAGGTGTCTGTCAGGATTC, AATGATACCAGTCAGGAATAAAGTC	537
chr1:111948172	GCAGGTGTCTGTCAGGATTC, AATGATACCAGTCAGGAATAAAGTC	537	GCAGGTGTCTGTCAGGATTC, AATGATACCAGTCAGGAATAAAGTC	537
chr1:111948199	GCAGGTGTCTGTCAGGATTC, AATGATACCAGTCAGGAATAAAGTC	537	GCAGGTGTCTGTCAGGATTC, AATGATACCAGTCAGGAATAAAGTC	537
chr6:146462770	GAGACAGTCATCCAGGCCAG, CGTCTTAAGCATGTATTCATTACTCTG	376	GAGACAGTCATCCAGGCCAG, CGTCTTAAGCATGTATTCATTACTCTG	376
chr6:146462771	GAGACAGTCATCCAGGCCAG, CGTCTTAAGCATGTATTCATTACTCTG	376	GAGACAGTCATCCAGGCCAG, CGTCTTAAGCATGTATTCATTACTCTG	376
chr6:146462792	GAGACAGTCATCCAGGCCAG, CGTCTTAAGCATGTATTCATTACTCTG	376	GAGACAGTCATCCAGGCCAG, CGTCTTAAGCATGTATTCATTACTCTG	376
chr6:146462804	GAGACAGTCATCCAGGCCAG, CGTCTTAAGCATGTATTCATTACTCTG	376	GAGACAGTCATCCAGGCCAG, CGTCTTAAGCATGTATTCATTACTCTG	376
chr6:146462864	GAGACAGTCATCCAGGCCAG, CGTCTTAAGCATGTATTCATTACTCTG	376	GAGACAGTCATCCAGGCCAG, CGTCTTAAGCATGTATTCATTACTCTG	376
chr6:146462894	GAGACAGTCATCCAGGCCAG, CGTCTTAAGCATGTATTCATTACTCTG	376	GAGACAGTCATCCAGGCCAG, CGTCTTAAGCATGTATTCATTACTCTG	376
chr6:146462926	GAGACAGTCATCCAGGCCAG, CGTCTTAAGCATGTATTCATTACTCTG	376	GAGACAGTCATCCAGGCCAG, CGTCTTAAGCATGTATTCATTACTCTG	376
chr6:146462943	GAGACAGTCATCCAGGCCAG, CGTCTTAAGCATGTATTCATTACTCTG	376	GAGACAGTCATCCAGGCCAG, CGTCTTAAGCATGTATTCATTACTCTG	376
chr1:4728531	TGCACCTCTCCTTACACCAG, CTGACCTGTGTGTGGAGGAC	551	TGCACCTCTCCTTACACCAG, CTGACCTGTGTGTGGAGGAC	551
chr1:4728555	TGCACCTCTCCTTACACCAG, CTGACCTGTGTGTGGAGGAC	551	TGCACCTCTCCTTACACCAG, CTGACCTGTGTGTGGAGGAC	551
chr1:4728570	TGCACCTCTCCTTACACCAG, CTGACCTGTGTGTGGAGGAC	551	TGCACCTCTCCTTACACCAG, CTGACCTGTGTGTGGAGGAC	551
chr1:4728583	TGCACCTCTCCTTACACCAG,	551	TGCACCTCTCCTTACACCAG,	551

	CTGACCTGTGTGTGGAGGAC		CTGACCTGTGTGTGGAGGAC	
chr1:4728597	TGCACCTCTCCTTACACCAG,<br>CTGACCTGTGTGTGGAGGAC	551	TGCACCTCTCCTTACACCAG,<br>CTGACCTGTGTGTGGAGGAC	551
chr1:4728627	TGCACCTCTCCTTACACCAG,<br>CTGACCTGTGTGTGGAGGAC	551	TGCACCTCTCCTTACACCAG,<br>CTGACCTGTGTGTGGAGGAC	551
chr1:4728628	TGCACCTCTCCTTACACCAG,<br>CTGACCTGTGTGTGGAGGAC	551	TGCACCTCTCCTTACACCAG,<br>CTGACCTGTGTGTGGAGGAC	551
chr1:4728737	TGCACCTCTCCTTACACCAG,<br>CTGACCTGTGTGTGGAGGAC	551	TGCACCTCTCCTTACACCAG,<br>CTGACCTGTGTGTGGAGGAC	551
chr1:4728923	CACGTGATGACCCATCTTCG,<br>TGCCTTGATGGTAGACTGAAATG	419	CACGTGATGACCCATCTTCG,<br>TGCCTTGATGGTAGACTGAAATG	419
chr1:4729000	CACGTGATGACCCATCTTCG,<br>TGCCTTGATGGTAGACTGAAATG	419	CACGTGATGACCCATCTTCG,<br>TGCCTTGATGGTAGACTGAAATG	419
chr1:4729050	CACGTGATGACCCATCTTCG,<br>TGCCTTGATGGTAGACTGAAATG	419	CACGTGATGACCCATCTTCG,<br>TGCCTTGATGGTAGACTGAAATG	419
chr1:4729089	CACGTGATGACCCATCTTCG,<br>TGCCTTGATGGTAGACTGAAATG	419	CACGTGATGACCCATCTTCG,<br>TGCCTTGATGGTAGACTGAAATG	419
chr1:4729090	CACGTGATGACCCATCTTCG,<br>TGCCTTGATGGTAGACTGAAATG	419	CACGTGATGACCCATCTTCG,<br>TGCCTTGATGGTAGACTGAAATG	419
chr1:4729110	CACGTGATGACCCATCTTCG,<br>TGCCTTGATGGTAGACTGAAATG	419	CACGTGATGACCCATCTTCG,<br>TGCCTTGATGGTAGACTGAAATG	419
chr1:4729136	CACGTGATGACCCATCTTCG,<br>TGCCTTGATGGTAGACTGAAATG	419	CACGTGATGACCCATCTTCG,<br>TGCCTTGATGGTAGACTGAAATG	419
chr16:43522348	GGCATTAGCCTTCACTGGAATAC,<br>TATTTTCTTGAGCCTGACTGCTG	507	GGCATTAGCCTTCACTGGAATAC,<br>TATTTTCTTGAGCCTGACTGCTG	507
chr16:43522351	GGCATTAGCCTTCACTGGAATAC,<br>TATTTTCTTGAGCCTGACTGCTG	507	GGCATTAGCCTTCACTGGAATAC,<br>TATTTTCTTGAGCCTGACTGCTG	507
chr16:43522380	GGCATTAGCCTTCACTGGAATAC,<br>TATTTTCTTGAGCCTGACTGCTG	507	GGCATTAGCCTTCACTGGAATAC,<br>TATTTTCTTGAGCCTGACTGCTG	507
chr16:43522387	GGCATTAGCCTTCACTGGAATAC,<br>TATTTTCTTGAGCCTGACTGCTG	507	GGCATTAGCCTTCACTGGAATAC,<br>TATTTTCTTGAGCCTGACTGCTG	507
chr16:43522391	GGCATTAGCCTTCACTGGAATAC,<br>TATTTTCTTGAGCCTGACTGCTG	507	GGCATTAGCCTTCACTGGAATAC,<br>TATTTTCTTGAGCCTGACTGCTG	507
chr16:43522467	GGCATTAGCCTTCACTGGAATAC,<br>TATTTTCTTGAGCCTGACTGCTG	507	GGCATTAGCCTTCACTGGAATAC,<br>TATTTTCTTGAGCCTGACTGCTG	507
chr16:43522470	GGCATTAGCCTTCACTGGAATAC,<br>TATTTTCTTGAGCCTGACTGCTG	507	GGCATTAGCCTTCACTGGAATAC,<br>TATTTTCTTGAGCCTGACTGCTG	507
chr16:43522496	GGCATTAGCCTTCACTGGAATAC,<br>TATTTTCTTGAGCCTGACTGCTG	507	GGCATTAGCCTTCACTGGAATAC,<br>TATTTTCTTGAGCCTGACTGCTG	507
chr16:43522534	GGCATTAGCCTTCACTGGAATAC,<br>TATTTTCTTGAGCCTGACTGCTG	507	GGCATTAGCCTTCACTGGAATAC,<br>TATTTTCTTGAGCCTGACTGCTG	507
