# Supplementary material for: RNA Editome in Rhesus Macaque Shaped by Purifying Selection
Source: PLoS Genet. 2014 Apr 10;10(4):e1004274. doi: 10.1371/journal.pgen.1004274 (PMC3983040; doi:10.1371/journal.pgen.1004274)
Supplement: Table S3 — Known human RNA-editing sites in coding regions. The percentage of “G” reads, together with the number of total reads (“A” and “G”) in each tissue sample is shown. *Yes: included in final list; DNA Depth: failed to pass inclusion criteria for read coverage; Filter: failed to pass the filter; #Ce: cerebellum, Te: testis, Pr: prefrontal cortex, Ki: kidney, Lu: lung, Mu: muscle, He: heart. (PDF) [file pgen.1004274.s015.pdf]

Table S3. Known human recoding RNA editing sites

Gene Name	hg18	rheMac2	Type	Filter*	DNA	RNA#						
						Ce	Te	Pr	Ki	Lu	Mu	He
GRIA2 (GluR2)	4:158477325	5:149561914	A→G	Yes	0 (93)	0.97 (30)	N/A	0.99 (159)	N/A	N/A	N/A	N/A
GRIA2 (GluR2)	4:158500744	5:149585132	A→G	Yes	0 (50)	0.72 (60)	N/A	0.75 (221)	N/A	1 (1)	N/A	N/A
IGFBP7	4:57670991	5:72269698	A→G	Yes	0 (10)	0.58 (24)	0.66 (100)	0.54 (35)	0.48 (302)	0.61 (182)	0.61 (64)	0.43 (79)
IGFBP7	4:57671043	5:72269646	A→G	DNA Depth	0 (3)	0 (9)	0 (12)	0 (5)	0.01 (111)	0.07 (53)	0.125 (8)	0 (40)
CYFIP2	5:156669386	6:153684514	A→G	Yes	0 (108)	0.79 (107)	0.1 (40)	0.74 (248)	0.02 (114)	0.04 (148)	0 (2)	0 (8)
GRIA4 (GluR4)	11:105309904	14:104538230	A→G	Basic Filter	0 (56)	0.57 (14)	N/A	1 (3)	N/A	N/A	N/A	N/A
KCNA1	12:4892003	11:5028364	A→G	Yes	0 (69)	0.33 (12)	N/A	0.4 (35)	N/A	N/A	N/A	N/A
BLCAP	20:35580947	10:26945949	A→G	Yes	0 (36)	0.09 (260)	0.08 (237)	0.06 (379)	0.08 (205)	0.03 (148)	0 (467)	0.05 (132)
BLCAP	20:35580977	10:26945919	A→G	Yes	0 (32)	0.14 (252)	0.1 (221)	0.13 (349)	0.12 (202)	0.13 (136)	0.01 (391)	0.08 (121)
BLCAP	20:35580986	10:26945910	A→G	Yes	0 (38)	0.18 (236)	0.20 (211)	0.19 (335)	0.22 (193)	0.20 (124)	0.02 (365)	0.2 (115)
GRIK1 (GluR5)	21:29875621	3:17076121	A→G	Basic Filter	0 (64)	0.67 (48)	N/A	0.76 (33)	N/A	N/A	N/A	N/A
GRAI3 (GluR3)	X:122426643	X:121683789	A→G	Basic Filter	0.01 (40)	0.92 (12)	N/A	0.97 (64)	N/A	1 (2)	N/A	N/A
GABRA3	X:151108975	X:150275345	A→G	Yes	0.03 (27)	0.71 (21)	0.08 (12)	0.93 (102)	N/A	N/A	1 (1)	N/A
FLNA	X:153233144	X:152404069	A→G	Yes	0 (54)	0.33 (49)	0.13 (175)	0.25 (68)	0.16 (102)	0.46 (473)	0.19 (193)	0.17 (96)
