# Supplementary material for: RNA Editome in Rhesus Macaque Shaped by Purifying Selection
Source: PLoS Genet. 2014 Apr 10;10(4):e1004274. doi: 10.1371/journal.pgen.1004274 (PMC3983040; doi:10.1371/journal.pgen.1004274)
Supplement: Table S4 — Statistics of public RNA-Seq data integrated in this study. $PFC: prefrontal cortex; &C: chimpanzee, H: human, R: rhesus macaque; #M: million reads; *Samples for cross-species comparison in Figure 5A ; @Samples for intra-species editing level comparison in Figure 3B ; %Samples for calculating intra-population CV values in Figures 3D & S7. (PDF) [file pgen.1004274.s016.pdf]

Table S4. Statistics of public RNA-Seq data integrated in this study.

Tissue	Description	Total Reads	Unique Reads		SRA
**PFC[$] (H[&])[*]**	76bp, single-end, Strand-nonspecific	24.33 M	14.37 M	59.08%	SRR306841
**Cerebellum (H)[*]**	76bp, single-end, Strand-nonspecific	46.76 M	25.68 M	54.92%	SRR306845, SRR306846
**Heart (H)[*]**	76 bp, single-end, Strand-nonspecific	30.90 M	16.07 M	52.01%	SRR306848, SRR306849
**Kidney(H)[*]**	76 bp, single-end, Strand-nonspecific	31.39 M	16.80 M	53.54%	SRR306853
**Testis (H)[*]**	76 bp, single-end, Strand-nonspecific	32.44 M	19.18 M	59.10%	SRR306858
**PFC (C)[*]**	101 bp, paired-end, Strand-nonspecific	44.47 M	16.65 M	37.44%	SRR306815
**Cerebellum (C)[*]**	76 bp, single-end, Strand-nonspecific	19.38 M	10.79 M	55.68%	SRR306818
**Heart (C)[*]**	76 bp, single-end, Strand-nonspecific	43.06 M	20.11 M	46.70%	SRR306820
**Kidney (C)[*]**	76 bp, single-end, Strand-nonspecific	34.17 M	23.03 M	67.40%	SRR306822
**Testis (C)[*]**	76 bp, single-end, Strand-nonspecific	26.75 M	13.10 M	48.99%	SRR306825
**Brain (R)[@]**	40 bp, paired-end, Strand-nonspecific	70.13 M	52.63 M	75.04%	SRX196317
**Heart (R)[@]**	40 bp, paired-end, Strand-nonspecific	70.50 M	46.29 M	65.67%	SRX196319
**Kidney (R)[@]**	40 bp, paired-end, Strand-nonspecific	63.78 M	44.24 M	69.36%	SRX196320
**Lung (R)[@]**	40 bp, paired-end, Strand-nonspecific	76.59 M	62.46 M	81.55%	SRX196322
**Muscle (R)[@]**	40 bp, paired-end, Strand-nonspecific	229.93 M	45.74 M	19.89%	SRX196323
**Testis (R)[@]**	40 bp, paired-end, Strand-nonspecific	56.98 M	43.90 M	77.04%	SRX196325
**Brain (R)[@]**	80 bp, paired-end, Strand-nonspecific	215.34 M	162.48 M	75.45%	SRX196326

**Heart (R)**@	80 bp, paired-end, Strand-nonspecific	218.39 M	158.66 M	72.65%	SRX196328
**Kidney (R)**@	80 bp, paired-end, Strand-nonspecific	217.28 M	147.47 M	67.87%	SRX196329
**Lung (R)**@	80 bp, paired-end, Strand-nonspecific	225.47 M	174.92 M	77.58%	SRX196331
**Muscle (R)**@	80 bp, paired-end, Strand-nonspecific	229.93 M	130.90 M	56.93%	SRX196332
**Testis (R)**@	80 bp, paired-end, Strand-nonspecific	230.88 M	177.40 M	76.83%	SRX196334
**Brain (R)**@	40 bp, paired-end, Strand-nonspecific	52.97 M	41.03 M	77.45%	SRX196335
**Heart (R)**@	40 bp, paired-end, Strand-nonspecific	72.20 M	50.24 M	69.58%	SRX196337
**Kidney (R)**@	40 bp, paired-end, Strand-nonspecific	80.78 M	43.48 M	53.82%	SRX196338
**Lung (R)**@	40 bp, paired-end, Strand-nonspecific	64.80 M	52.50 M	81.02%	SRX196340
**Muscle (R)**@	40 bp, paired-end, Strand-nonspecific	68.54 M	53.19 M	77.60%	SRX196341
**Testis (R)**@	40 bp, paired-end, Strand-nonspecific	55.61 M	44.75 M	80.48%	SRX196343
**Brain (R)**	100 bp, paired-end, Strand-nonspecific	40.92 M	16.32 M	39.89 %	SRX066572
**Brain (R)**@	76 bp, single-end, Strand-nonspecific	19.07 M	9.74 M	51.06%	SRX081922
**Brain (R)**@	76 bp, single-end, Strand-nonspecific	22.55 M	11.07 M	49.09%	SRX081923
**Brain (R)**@	101 bp, paired-end, Strand-nonspecific	42.92 M	21.35 M	49.74 %	SRX081924
**Cerebellum (R)**@	76 bp, single-end, Strand-nonspecific	25.53 M	12.21 M	47.82 %	SRX081925
**Cerebellum (R)**@	76 bp, single-end, Strand-nonspecific	21.14 M	10.94 M	51.73 %	SRX081926
**Heart (R)**@	76 bp, single-end, Strand-nonspecific	28.64 M	9.95 M	34.8 %	SRX081927
**Heart (R)**@	76 bp, single-end, Strand-nonspecific	20.82 M	8.85 M	42.51%	SRX081928
