# Supplementary material for: RNA Editome in Rhesus Macaque Shaped by Purifying Selection
Source: PLoS Genet. 2014 Apr 10;10(4):e1004274. doi: 10.1371/journal.pgen.1004274 (PMC3983040; doi:10.1371/journal.pgen.1004274)
Supplement: Table S5 — P-values of one-tail Wilcoxon tests for four classes of motif. *M: matched motif; 5′ M: 5′ matched; 3′ M: 3′ Matched; N: Not matched. (PDF) [file pgen.1004274.s017.pdf]

Table S5. P-values of one-tail Wilcoxon tests for four classes of motif

Tissue	M *vs* 5' M*	M *vs* 3' M	M *vs* N	5' M *vs* 3'M	5' M *vs* N	3' M *vs* N
**Muscle**	6.04E-02	3.53E-02	1.81E-07	4.43E-01	3.36E-04	3.50E-04
**Heart**	3.16E-01	1.09E-03	1.47E-08	1.37E-02	1.36E-06	2.48E-03
**Lung**	1.14E-03	1.25E-09	1.89E-13	8.26E-03	2.95E-06	4.16E-03
**Kidney**	1.18E-01	5.24E-05	4.33E-07	1.33E-02	2.10E-04	4.58E-02
**Testis**	2.23E-03	5.18E-07	3.59E-16	4.31E-02	2.84E-08	2.76E-05
**Prefrontal Cortex**	3.26E-08	1.66E-21	8.34E-27	3.32E-04	3.96E-10	1.75E-04
**Cerebellum**	1.53E-19	3.76E-50	1.29E-57	8.07E-07	3.81E-17	3.12E-06
